# Ancient Origin of the New Developmental Superfamily DANGER

**DOI:** 10.1371/journal.pone.0000204

**Published:** 2007-02-14

**Authors:** Nikolas Nikolaidis, Dimitra Chalkia, D. Neil Watkins, Roxanne K. Barrow, Solomon H. Snyder, Damian B. van Rossum, Randen L. Patterson

**Affiliations:** 1 Biology Department, Pennsylvania State University, University Park, Pennsylvania, United States of America; 2 The Sidney Kimmel Cancer Institute, Johns Hopkins University, School of Medicine, Baltimore, Maryland, United States of America; 3 The Solomon H. Snyder Department of Neuroscience, Johns Hopkins University, School of Medicine, Baltimore, Maryland, United States of America; 4 Department of Pharmacology and Molecular Science, Johns Hopkins University, Baltimore, Maryland, United States of America; 5 Department of Psychiatry and Behavioral Sciences, Johns Hopkins University, Baltimore, Maryland, United States of America; The Wellcome Trust Sanger Institute, United Kingdom

## Abstract

Developmental proteins play a pivotal role in the origin of animal complexity and diversity. We report here the identification of a highly divergent developmental protein superfamily (DANGER), which originated before the emergence of animals (∼850 million years ago) and experienced major expansion-contraction events during metazoan evolution. Sequence analysis demonstrates that DANGER proteins diverged via multiple mechanisms, including amino acid substitution, intron gain and/or loss, and recombination. Divergence for DANGER proteins is substantially greater than for the prototypic member of the superfamily (Mab-21 family) and other developmental protein families (e.g., WNT proteins). DANGER proteins are widely expressed and display species-dependent tissue expression patterns, with many members having roles in development. DANGER1A, which regulates the inositol trisphosphate receptor, promotes the differentiation and outgrowth of neuronal processes. Regulation of development may be a universal function of DANGER family members. This family provides a model system to investigate how rapid protein divergence contributes to morphological complexity.

## Introduction

A central question in evolutionary developmental biology is how genetic diversity and complexity have contributed to the origin and the remarkable diversification of the animal body plan. Development of the animal body plan is controlled by large gene regulatory networks, which consist of transcription factors, their target cis-regulatory modules, and signaling molecules [1], [2]. As opposed to the early stages of body plan formation which involve specification of developing components and their spatial patterning, the later stages of development involve cellular differentiation [2]. Genes that promote cellular differentiation display high levels of sequence divergence and have contributed to morphological complexity [2], [3]. Therefore, identifying new genes involved in cell differentiation and characterizing their evolutionary history is fundamentally important towards understanding the origin of animal complexity and diversity.

We recently discovered the inositol 1,4,5-triphosphate receptor (IP_3_R) binding protein DANGER, which is an allosteric regulator of IP_3_R Ca^2+^ dependence [4]. The human (*Homo sapiens*; Hs) DANGER protein consists of 547 amino acids and is predicted to contain a small portion of the Male abnormal 21 (Mab-21) domain [4]. The Mab-21 domain was first reported in the nematode *Caenorhabditis elegans* [5] and has been defined as a domain of 360 amino acids. Proteins containing the Mab-21 domain are members of a small and highly conserved family with single genes in invertebrate and two genes in vertebrate species [6], [7]. Members of the MAB-21 family control cellular differentiation in *C. elegans*, *Danio rerio*, and *Mus musculus* [5]–[10]. When MAB-21 members are genetically deleted, gross morphological changes in developing animals ensue [5]–[10]. The Gestalt Domain Detection Algorithm (GDDA) is a “seeding” algorithm which can identify highly divergent domains [11], [12]. GDDA analysis predicts that DANGER contains almost 90% of the Mab-21 domain sequence (Figure S1).

## Results and Discussion

### Identification and phylogenetic analysis of the DANGER proteins

**Figure 1:**
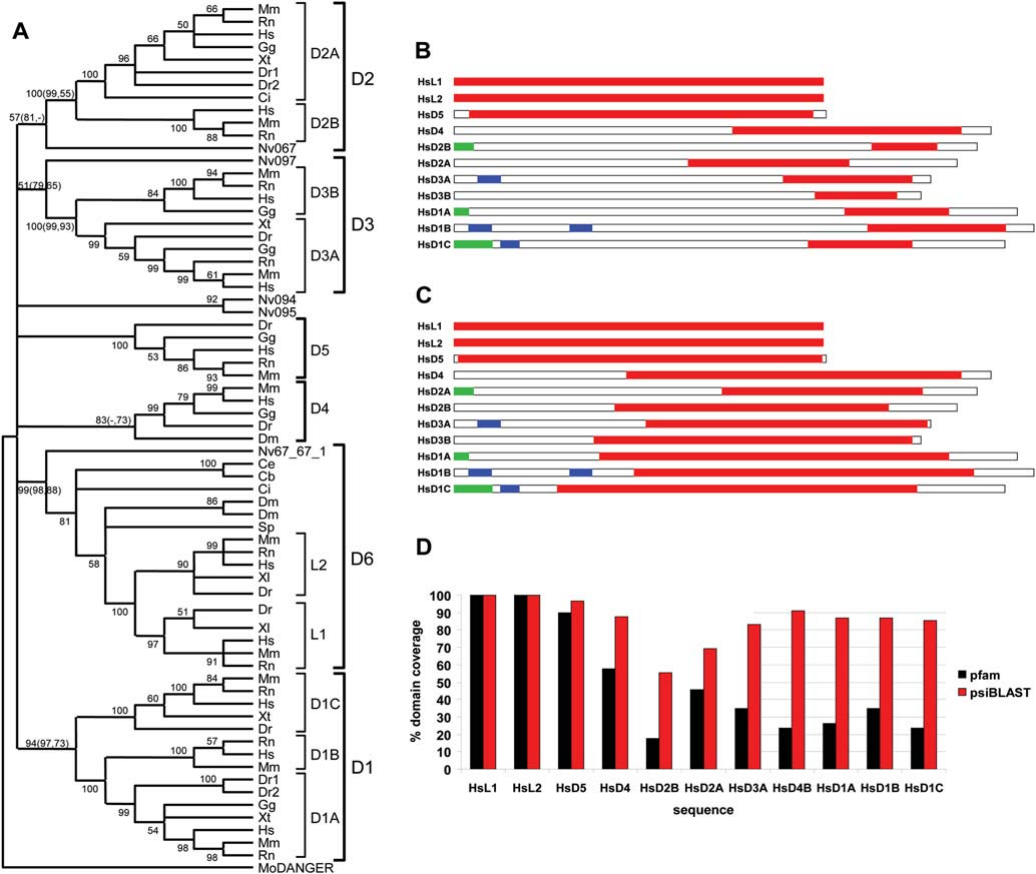
DANGER proteins originated early in metazoan evolution and encode a highly divergent MAB-21 domain. (A) Neighbor-joining (NJ) consensus tree of the DANGER superfamily defines orthologous relationships among DANGER sequences (D1–D6) from vertebrates (Dr, *Danio rerio*; Xl, *Xenopus laevis*; Xt, *Xenopus tropicalis*; Gg, *Gallus gallus*; Rn, *Rattus norvegicus*; Mm, *Mus musculus*; Hs, *Homo sapiens*), invertebrates (Ci, *Ciona intestinalis*; Sp, *Strongylocentrotus purpuratus*; Dm, *Drosophila melanogaster*; Ce, *Caenorhabditis elegans*; Cb, *Caenorhabditis briggsae*, Nv, *Nematostella vectensis*), and the choanoflagellate (Mo, *Monosiga ovata*). Numbers at branches are bootstrap values from the NJ analysis. Bootstrap and quartet puzzling support values from maximum parsimony (MP) and maximum likelihood (TREE-PUZZLE) analyses, respectively, are given in parentheses. (B) Domain prediction analysis using the Pfam Mab-21 profile, containing four sequences, identifies a partial Mab-21 domain (showed as red boxes) in the human DANGER (HsD1-5) proteins. Predicted signal peptides and transmembrane regions are depicted by green and blue boxes, respectively. (C) Domain prediction analysis, using a Mab-21 profile generated by psi-BLAST containing 63 animal sequences, results in Mab-21 domain extension in all human DANGER proteins. (D) Quantification of Mab-21 domain coverage as predicted by the Pfam and the psiBLAST-generated Mab-21 profiles in all human DANGER proteins. Similar Mab-21 domain extension is observed between orthologous DANGER sequences from other species.

We used the HsMAB21L1 and HsDANGER proteins as queries to search several species databases for similar protein sequences. This analysis revealed the presence of multiple metazoan proteins, denoted as DANGER (D) proteins (Table S1). Phylogenetic analyses of these sequences classify them into six families (D1–D6; Figure 1A). In our analysis the previously reported [6] highly conserved MAB-21 protein family is extended, and herein is denoted as D6. A unique DANGER sequence from the unicellular choanoflagellate species *Monosiga ovata* assumes an outgroup position (Figure 1A and Figure S2). The sea anemone *Nematostella vectensis* (Anthozoa, Cnidaria) is a phylogenetically basal animal which is composed of only 11 cell types [13]. Notably, this species contains multiple DANGER sequences which cluster with different DANGER families, indicating that the DANGER superfamily was expanded and already diversified in the cnidarian-bilaterian ancestor (Figure 1A and Figure S2). *C. elegans* and *D. melanogaster* genomes likely lost many DANGER genes, suggesting a contraction of the DANGER superfamily in these protostomes; an evolutionary pattern also documented for the developmentally important WNT superfamily [14]. In deuterostomes (e.g. echinoderms and chordates), additional gene duplication events led to the emergence of new DANGER families.

With the exception of some anthozoan sequences, the orthologous DANGER groups are well defined. In contrast, relationships among paralogous groups remain ambiguous even when different protein regions, sequence datasets, and phylogenetic methods (Neighbor-Joining, Maximum Parsimony, and Maximum Likelihood) are used (Figure 1A and Figure S2). Genetic distances produced by several independent methods (data not shown) and the multiple sequence alignment (Figure S3) further demonstrate the divergence of paralogous DANGER sequences. This pattern of evolution resembles that of the WNT superfamily, in which the relationships among paralogous groups are also unclear [15], [16].

### Domain analysis of the DANGER proteins

Reverse position specific (rps)-BLAST similarity searches (Figure 1B) reveal that most DANGER sequences encode only a C-terminal portion of the Mab-21 domain. However, the Mab-21 domain consensus sequence (profile) in the Conserved Domain Database (CDD) is composed of only four sequences from the highly conserved D6 family. Therefore, we generated a new Mab-21 profile utilizing all D1–6 animal sequences (table S1), except the anthozoan sequences (see Materials and Methods section). This analysis predicts that all DANGER proteins contain more than 55% of the Mab-21 domain and most of them score higher than 70% (Figure 1C, 1D, and Figure S4, S5).

### Evolution of the Mab-21 domain sequence

**Figure 2:**
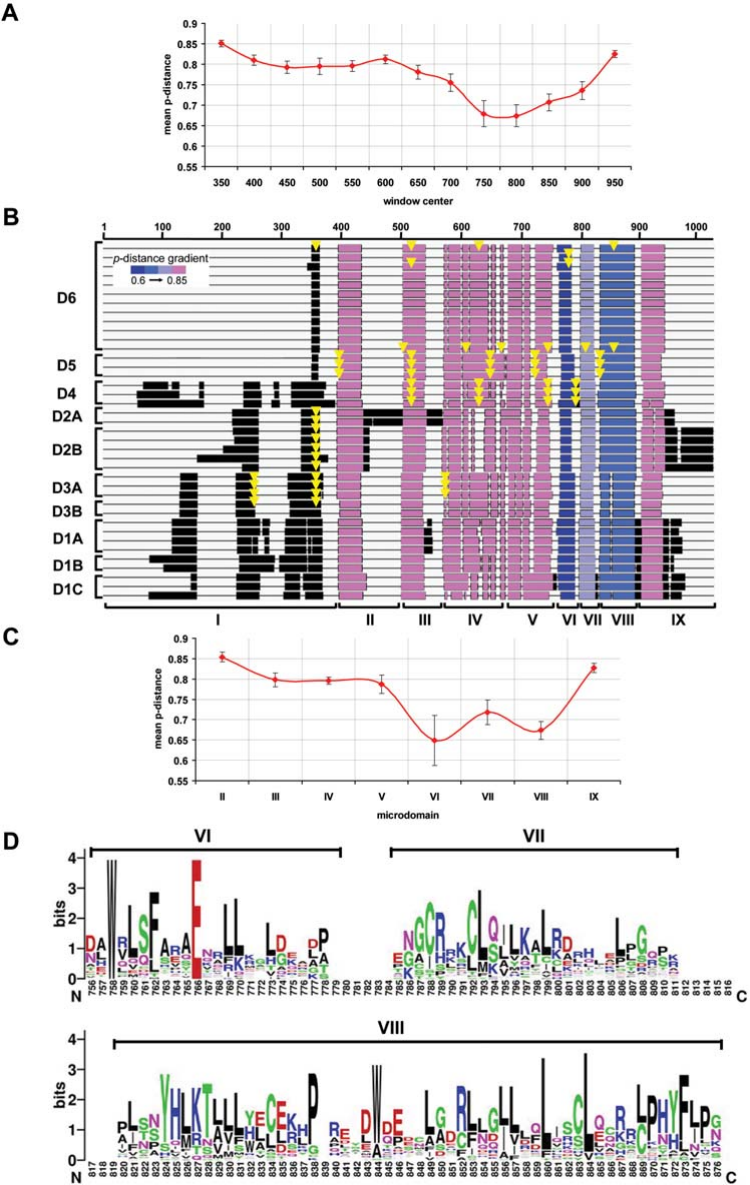
Patterns of sequence evolution along the Mab-21 domain. (A) Graphical representation of the *p*-distances calculated along the multiple sequence alignment of representative DANGER proteins with a sliding window of 100 amino acids and a step of 50 amino acids. Bars represent standard errors of mean *p*-distance values. (B) Multiple sequence alignment of representative DANGER sequences (D1–D6) in block format. Microdomains II to IX are colored according to the mean value of the proportion of amino acid differences (*p*-distance) among all sequences. Intron positions are mapped as yellow arrows. (B) Graphical representation of the *p*-distances for each microdomain. Bars represent standard errors of mean *p*-distance values. (D) Pattern of sequence conservation (logo) along the three most conserved Mab-21 microdomains (VI–VIII).

To identify functionally important regions in the Mab-21 domain sequence, we calculated the *p*-distances across the multiple sequence alignment (MSA) of representative DANGER proteins (Figure 2A). This analysis shows the Mab-21 domain sequence is highly divergent among the paralogous DANGER sequences. In addition, the observed sharp changes in the *p*-distances along the MSA suggest that the Mab-21 domain can be roughly divided into two regions: the highly diverged N-terminus (positions in MSA 300–750) and the more conserved C-terminus (positions in MSA 751–900) (Figure 2A). Taking into account the presence of long insertions and/or deletions (indels) along the MSA we divided the Mab-21 domain into nine microdomains (Figure 2B) and calculated the *p*-distances for each microdomain (Figure 2C). The most conserved regions of the Mab-21 domain are microdomains VI–VIII (Figure 2 and Figure S6, S7). We hypothesize that microdomains VI–VIII represent conserved functional units of the DANGER proteins, while microdomain I may provide temporal and/or spatial specificity.

Microdomain I sequences are highly diverged and can be unambiguously aligned only among orthologous sequences (Figure S3). This microdomain in D1A–C, D2B, and D3A is predicted to encode signal peptides and/or transmembrane regions (see Materials and Methods section) (Figure 1B and 1C). Our phylogenetic analysis suggests that the common ancestor of vertebrates contained all three DANGER gene-lineages encoding localization signals (Figure 1A and Figure S2). Coupling the aforementioned observations with the presence of introns at the C-terminus of microdomain I sequences (Figure 2B), we hypothesize that DANGER sequences have acquired microdomain I through exon-shuffling [17] early in vertebrate evolution. This acquisition likely enabled some DANGER proteins to function in different cellular compartments or even to be secreted [18]–[25].

**Figure 3:**
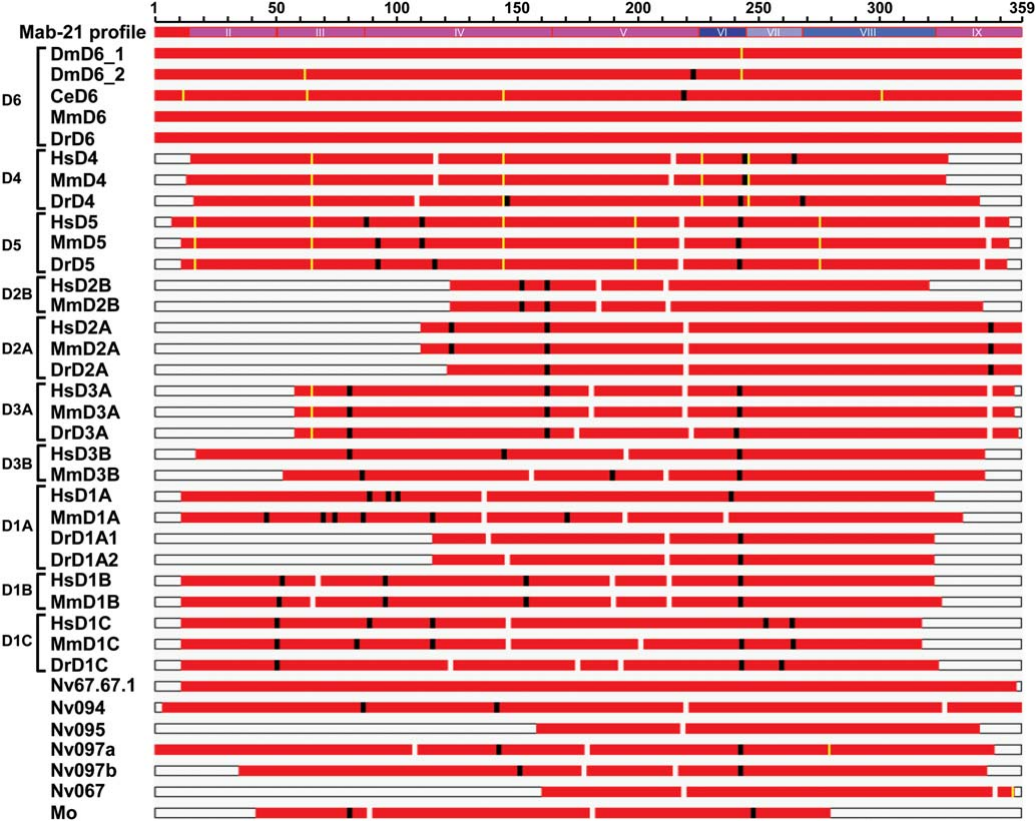
Evolutionary patterns of the Mab-21 domain sequence. Rps-BLAST pairwise alignments between the psi-BLAST generated Mab-21 profile and DANGER sequences. Comparison of insertions (in profile sequence; black boxes) and deletions (in profile sequence; white boxes), and correspondence of indels (>3 amino acids) with intron positions (yellow boxes). For comparison, all DANGER sequences are mapped onto the Mab-21 profile sequence according to pairwise alignment coordinates. The microdomains II–VIII are also shown. Species abbreviations are as in Figure 1A.

To gain insight into the evolutionary processes that shaped the DANGER sequences, we examined the location of the indels within the Mab-21 domain sequence. For this purpose we utilized the pairwise alignments between the psi-BLAST-generated Mab-21 profile and each DANGER sequence. In order to compare these alignments, all DANGER sequences were mapped onto the Mab-21 profile sequence according to the pairwise alignment coordinates. This analysis reveals several conserved indels in both orthologous and paralogous groups (Figure 3 and Figure S8, S9). Indels conserved among orthologous sequences suggest that these occurred in the ancestral sequence of a particular group. Conservation of indels between paralogous sequences, from anthozoa to mammals, suggests that these indels occurred in the common ancestor of the DANGER sequences very early in metazoan evolution (Figure S9, S10). Several conserved indels tend to coincide with intron positions (Figure 3 and Figure S8, S9). Taking into account that frequent intron gain and/or loss occurred during DANGER evolution (Figure S2), we speculate that intron mobility could have been responsible for the fragmentation of the Mab-21 domain sequence. This speculation is supported by similar observations within the Wnt domain sequence (data not shown).

Intron gain and/or loss cannot explain the presence of all observed indels. These additional indels could have resulted from DNA polymerase slippage [26]. However, this is unlikely, as indels identified were usually longer than nine nucleotides (3 amino acids). Such long indels have been identified only in non-coding regions and have been attributed to recombination events followed by unequal cross-over [26]. Thus, the indels in the Mab-21 domain sequence could have been the result of recombination. To test this hypothesis, we used comparative genomic analyses and found that in many cases DANGER genes are in conserved synteny among all species studied (Figure S10). In addition, the genomic regions flanking DANGER genes contain a high number of transposable elements (TEs), which are conserved among paralogous but not orthologous sequences (Figure S11, S12). The latter observation indicates that within *H. sapiens*, *M. musculus*, and *D. rerio* genomes the TEs flanking DANGER genes have been multiplied in each genome independently through different mechanisms including recombination (Figure S11 and S12) [27]–[29]. Thus, we hypothesize that recombination among TEs could provide an alternative explanation for the presence of indels along the Mab-21 domain sequence. Three findings support this notion. (A) The HsD3B is a candidate gene for the Smith-Magenis syndrome, which involves chromosome deletion through recombination among repeats [30], [31]. (B) The HsD4 is flanked by numerous repeats similar to these flanking the breakpoint cluster region (BCR) gene (Figure S11). The human BCR gene is located at the site of the translocation breakpoint found in chronic myeloid leukemia [32], which like Smith-Magenis region is a “hot-spot” for recombination. (C) The mRNA sequences of D1C, D3A, D4 and D5 genes in human and mouse contain TEs (table S2). These TEs within DANGER genes are likely the result of recent independent transpositions, since orthologous genetic regions contain different classes of repeats (table S2). Overall, these observations indicate that TEs have contributed to the evolution of DANGER gene sequences and support the notion that recombination events among TEs probably contributed to the genetic variation of DANGER sequences during vertebrate evolution.

In the morphologically simple animal *N. vectensis*, DANGER gene sequences encode either a complete or a partial Mab-21 domain (Figure S5 and S8). In addition, the genetic distances among all *N. vectensis* DANGER sequences are rather high (Figure S2). Furthermore, these genes reside in genomic regions that contain many TEs (data not shown). The above observations suggest that both complete and partial Mab-21 domains likely existed before the emergence of bilateria and that recombination among TEs could have been responsible for the increased variation among anthozoan DANGER paralogs.

### Function of DANGER proteins in development

D6 proteins have been implicated in development and cellular differentiation. During *C. elegans* development, the Mab-21 protein is required for the choice of alternate cell fates and the formation of sensory organs in the male nematode tail [5]. Mutations of the *mab-21* gene affect movement, body shape, and fertility [5]. In *D. rerio* D6 genes were found to be highly expressed in the differentiating eye, the midbrain, and the neural tube [6]. In *Xenopus laevis* D6 proteins are required for the completion of gastrulation and neuronal development [23]. In *M. musculus* the expression of D6 genes is important for the development of the embryonic brain, eye, limbs, and neural crest derivatives. In particular, D6 deficient mice show defects in eye formation (absence of lens), notochord and neural tube development, organogenesis, and axial turning, resulting in death at mid-gestational stage [9], [10], [25]. We searched the PUBMED database to retrieve entries associated with the remaining DANGER proteins and found many members within high-throughput studies. In these reports, DANGER proteins from all six families are described within large developmental gene and protein networks [18], [20]–[22], [24]. Although, these studies do not provide a specific function for each DANGER protein, they suggest that DANGER proteins seem to be involved in a variety of developmental processes.

### Expression of DANGER genes

**Figure 4:**
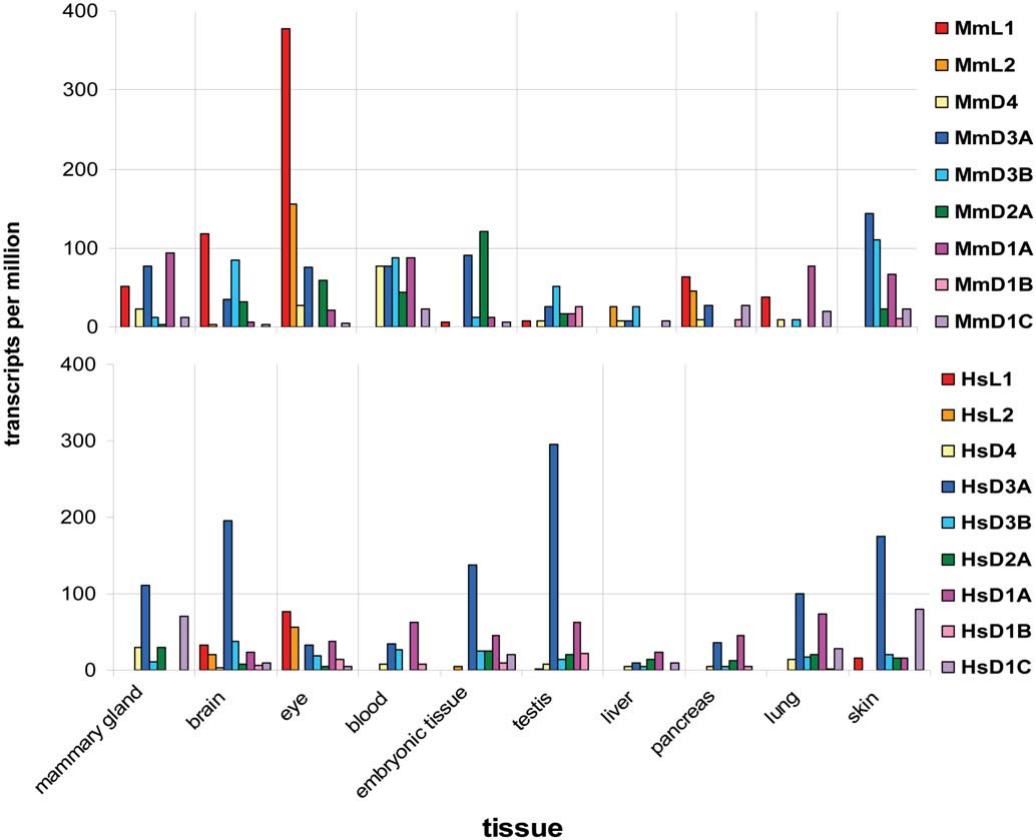
Analysis of expressed sequence tags (ESTs) show that mouse and human DANGER sequences exhibit different transcriptional patterns. Upper: Plot depicting the expression levels of the mouse DANGER sequences in different tissues. Lower: Plot depicting the expression levels of the human DANGER sequences in different tissues. (Additional information and the Unigene identification numbers for each gene used in this analysis can be found in Figure S13).

To identify the expression profile of the DANGER genes over different tissues and developmental stages, we utilized the expression (EST) data from the UniGene database for human and mouse paralogs (Figure S13). We found that DANGER sequences are differentially expressed in several tissues at various developmental stages (Figure S13) suggesting functional divergence between paralogous DANGER sequences. In addition, we compared the expression levels among orthologous DANGER pairs in comparable human and mouse tissues (Figure 4). In all cases the observed differences between the orthologous pairs are statistically significant (chi-square test, α = 0.01), suggesting that DANGER genes exhibit species specific expression levels.

We then tested the expression of the human D1A gene by northern analysis (Figure S14) and found it to be widely expressed agreeing with the EST analysis (Figure S13). We next performed immunohistochemistry on mouse embryos at embryonic age E13.5 (13.5 days post conception) and observed that D1A primarily expressed in the spinal cord. In contrast, mouse embryos at embryonic age E18 showed expression throughout the body (Figure S14). In addition, we examined adult mouse sections and determined that D1A is highly expressed in a number of terminally differentiated cells such as myotubes in skeletal muscle and crypt cells in the small intestine (Figure S14).

### DANGER1A is involved in cell differentiation

**Figure 5:**
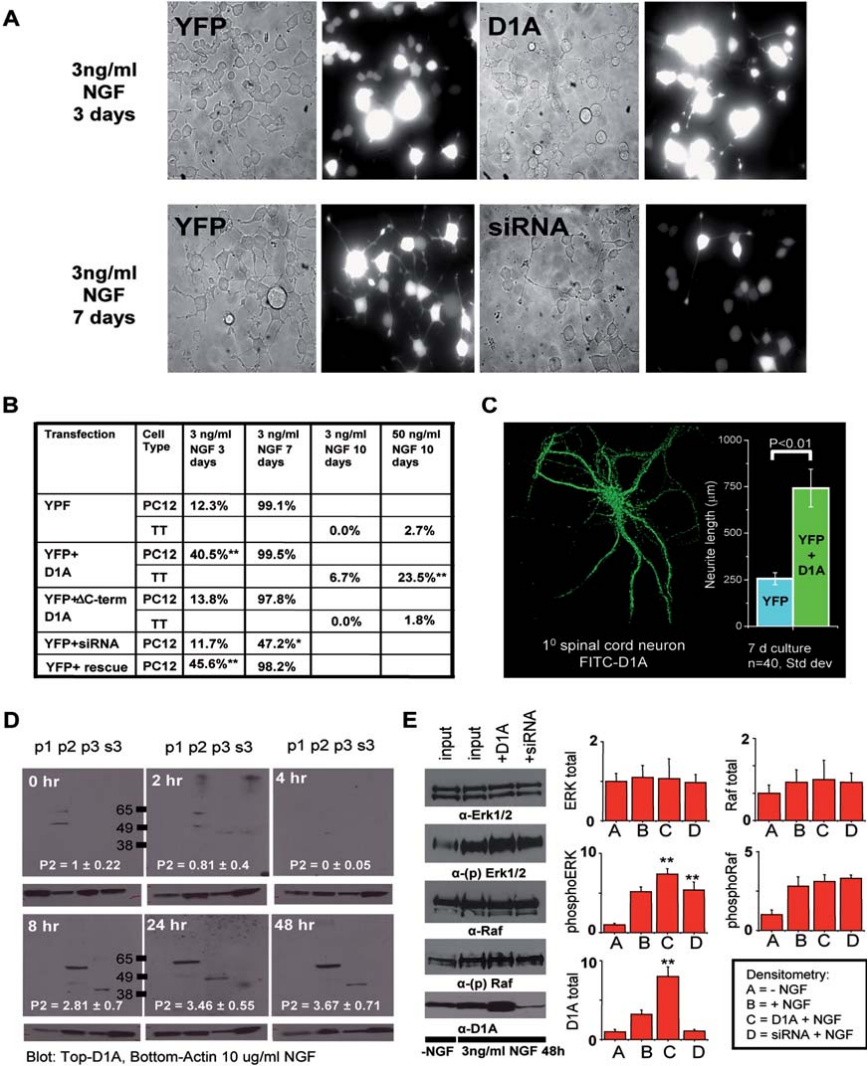
DANGER1A increases neurite outgrowth in response to NGF signaling. (A) Top: 40× Phase picture and 485 nm emission of PC12 cells transfected with YFP-alone or YFP+MYC-tagged D1A stimulated with NGF (3 ng/ml 3 days). PC12 cells expressing D1A have increased neurite outgrowth. Bottom: Phase picture and 485 nm emission of PC12 cells transfected with YFP-alone or YFP+siRNA against rat D1A stimulated with NGF (3 ng/ml 7 days). PC12 cells depleted of endogenous D1A using siRNA have decreased neurite outgrowth. (B) Quantification of neurite outgrowth in PC12 and TT cells. (100 cells counted in 3 independent experiments) (C) Left: Immunofluorescent staining of mouse primary spinal cord neurons with Alexa488-conjugated DANGER1A antibody. Inset: Quantification of spinal cord neurite length in neurons overexpressing YFP alone or YFP+D1A. (D) Western analysis of D1A expression in the p1 (nuclear), p2 (heavy ER and mitochondria), p3(light ER and vesicles), or s3 (cytosol) cell fractions over a time course of 48 h stimulation with NFG (10 ng/ml). These individual blots were developed simultaneously on the same film. The numbers inside the blots quantify the change in D1A distribution over time course (Error: SEM; p<0.01 n = 4). (E) Right: Western analysis of the MAP-kinase proteins ERK1/2, phospho-ERK1/2, Raf, phospho-Raf, and D1A levels in PC12 cells transfected with either YFP, YFP+D1A, or YFP+siRNA D1A. Cells were treated with vehicle (water), or 3 ng/ml NGF for 48 hours. Left: Quantification of changes in total and phospho-ERK1/2 and total D1A by scanning densitometry. All values are expressed as fold-change vs. control; ERK-(Error: SEM; p<0.05 n = 3); D1A-(Error: SEM; p<0.01 n = 3)

As D1A is expressed in terminally differentiated neurons (Figure S14), we used PC12 rat adrenal gland pheochromocytoma cells as a model system for cellular differentiation. This cell line can be induced to differentiate by application of nerve growth factor (NGF). Differentiated PC12 cells can be defined as possessing neurite extensions ≥2.5 times the length of the cell body [33]. PC12 cells overexpressing D1A display a ∼3-fold increase in neurite length in response to low concentrations of NGF at early time points (Figure 5A and 5B). Inversely, PC12 cells depleted of D1A using siRNA display lower levels of differentiation at late time-points (∼50% reduction at 7 days).

We have shown that the most conserved regions of the Mab-21 domain are microdomains VI–VIII (Figure 2 and Figure S6, S7) and we have predicted that these domains may represent functional units within the Mab-21 domain. To test this prediction we engineered a deletion in D1A (amino acids 440–553; microdomains VIII–IX) and we assessed neurite outgrowth in PC12 cells. We found that ^Δ^C-termD1A is unable to promote NGF-induced neurite outgrowth in PC12 cells (^Δ^C-termD1A; Figure 5B), supporting our original prediction.

In addition to PC12 cells that respond to NGF, we used the human thyroid-derived neuroendocrine tumor (TT) cells which are defective in NGF-signaling, although they express cell-surface tyrosine kinase A (Trk-A) NGF receptors and can manifest neurites following transfection with constitutively active serine/threonine protein kinase Raf [34]. While TT cells express the full-length transcript of D1A as demonstrated by RT-PCR (data not shown), these cells do not express the full-length D1A protein (Figure S15) [4]. Therefore, we examined the influence of rescued-overexpression of full-length D1A on neurite extension in TT cells (Figure 5B and Figure S15). D1A overexpression induced neurite outgrowth in a small population of TT cells at very low levels of NGF (3 ng/ml), and provided a 10-fold increase in neurite extension when cells were treated with 50 ng/ml NGF. By contrast, NGF-stimulation (3 ng/ml 10 days) of TT cells overexpressing YFP-alone (control) did not cause neurite extension, while higher concentrations of NGF (50 ng/ml) induced neurite outgrowth in a small but reproducible population of control cells (∼2%). As p75 has a low affinity for NGF, we presume that D1A is activated predominately by Trk-A receptors at the concentrations utilized.

Finally, to obtain physiological relevance of the above results, we overexpressed D1A and assessed neurite length in primary spinal cord neurons. In 7 day cultures, we observed a ∼2.5 fold increase in neurite outgrowth (Figure 5C), signifying the ability of D1A to participate in cell differentiation pathways.

### DANGER1A functions in the NGF pathway

We next examined whether endogenous D1A expression and localization could be influenced by NGF treatment. In PC12 cells, we observed a biphasic response of D1A expression and localization, with increased expression 24 h post-treatment (Figure 5D). We observe a transient depletion of D1A at 4 hr post-NGF treatment with increasing expression after 8 hrs (n = 4). Following, we examined whether D1A expression could affect two NGF-linked kinases, Raf and the mitogen-activated protein kinase (ERK1/2) (Figure 5E). In differentiated PC12 cells, overexpression or siRNA-depletion of D1A did not affect Raf protein levels or phosphorylation at the time-points tested. Conversely, in PC12 cells overexpressing D1A the phosphorylation levels of ERK1/2 were increased, while protein levels were unaltered. Compared to NGF-stimulated PC12 control cells, siRNA-depletion of D1A did not affect the levels of ERK1/2 phosphorylation. However, full depletion of D1A via siRNA was not possible as NGF-treatment alone augments D1A expression. These data fit well with our recent evidence that D1A can influence IP_3_R mediated Ca^2+^ activity, which is an intermediary of Trk-A receptor activation and ERK1/2 activity [35]. Our functional studies, together with previous reports [5]–[10], [18], [20]–[25] demonstrate that DANGER proteins have the capacity to function in developmental processes.

The phylogenetic grouping, similar domain content, expression profiles, and general activity in developmental pathways demonstrate that DANGER proteins comprise a new developmental superfamily. This superfamily likely remained undiscovered due to its high sequence divergence (mean *p*-distance among paralogs is 0.756±0.009), which far exceeds the divergence among the well studied WNT proteins (mean *p*-distance of WNT domains 0.561±0.016).

**Figure 6:**
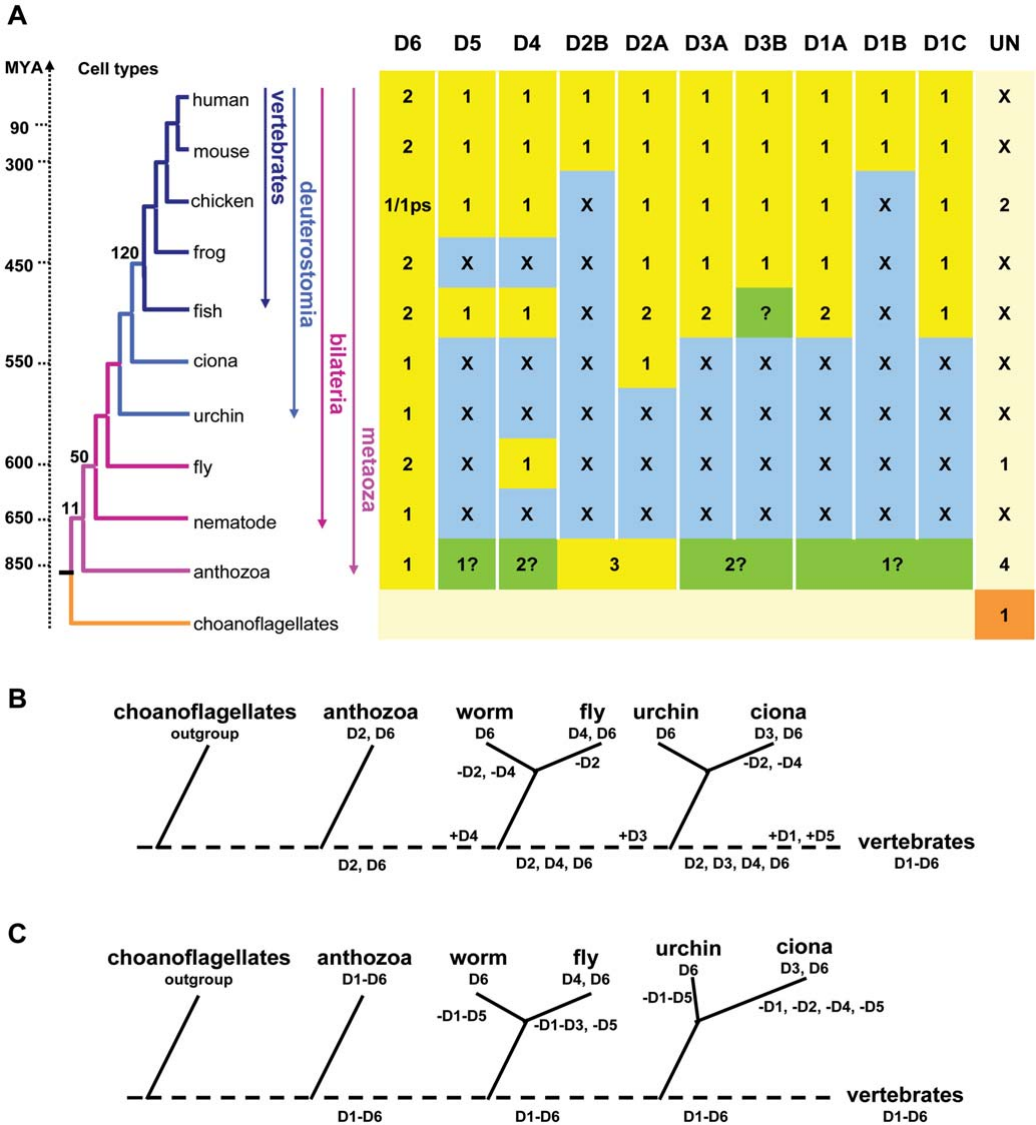
Distribution and evolution of DANGER genes in metazoa. (A) The tree on the left summarizes the phylogenetic relationships of the informative species used. Numbers at nodes represent cell types adopted from [13]. Yellow boxes indicate the presence of a DANGER gene in a particular taxon. Numbers correspond to the number of genes found in each taxon. “X” indicates the absence of a DANGER gene in a particular taxon (blue boxes). Uncertain orthologous relationships are indicated by question marks (green boxes). “UN” indicates the unclassified DANGER proteins or the ones that could not be unconditionally assigned to a specific group. The choanoflagellate sequence from *M. ovata* assumes outgroup position in all phylogenetic analyses. Divergence times in million years ago (MYA) were taken from references [58], [59]. (B–C) Two alternative evolutionary scenarios explain the evolution of DANGER families in metazoa. Both scenarios presume that the genome sequence of the vertebrate ancestor encoded at least six DANGER lineages, namely D1–D6. (B) According to the first scenario, anthozoa contain two (D2, D6) of the six vertebrate DANGER lineages, while the remaining DANGER lineages have evolved by repeated cycles of gene birth (+) and death (−). (C) In the second scenario anthozoa species contain sequences orthologous to all vertebrate DANGER groups. This scenario presupposes that in ecdysozoa, hemichordates, and urochordates four DANGER groups were lost. Dotted lines correspond to ancestral stages. The order of events is presented according to the species tree.

Our findings demonstrate that the newly discovered superfamily of DANGER proteins is involved in developmental processes and is of ancient origin. This superfamily has continually been expanded and contracted during metazoan evolution (Figure 6A). The expression and function of DANGER proteins in cells which can terminally differentiate suggest that DANGERs may be part of the genetic toolkit responsible for the emergence of different cell types during animal evolution (Figure 6A). Likely, DANGER represents part of the eukaryotic genome that existed before the emergence of multicellular animals [36]. This is supported by the existence of at least one DANGER homolog in choanoflagellates and the apparent absence of DANGER sequences in other eukaryotic kingdoms. The *N. vectensis* genome encodes at least 14 proteins containing the Mab-21 domain, suggesting an ancient or a species-specific expansion of the DANGER superfamily. In contrast, in protostome, hemichordate, and urochordate genomes this family has experienced a remarkable contraction (Figure 6A).

From our data we cannot conclude how many DANGER gene-lineages existed in the bilaterian ancestor (Figure 6B and 6C). The most conservative evolutionary scenario suggests that at least two DANGER gene-lineages existed in the common ancestor of anthozoa and bilateria. According to this scenario the DANGER repertoire found in invertebrate and vertebrate species is the result of multiple gene birth and death events (Figure 6B) [37]. Alternatively, the anthozoan DANGER gene-lineages could have persisted during metazoan evolution resulting in the vertebrate DANGER repertoire (Figure 6C). The latter suggests the presence of ancient genetic complexity in anthozoa and loss of genes in invertebrates as it has been previously suggested [14].

To determine which microdomains have conserved functions or represent evolutionary innovations would require functional characterization of the DANGER proteins in both basal and crown animals. Elucidating the relationship between microdomain evolution and functional divergence would provide insights on how genetic and cellular complexity contributes to morphological diversity. In conclusion, this evolutionary study of DANGER genes offers a view into the workshop of a busy evolutionary tinker.

## Materials and Methods

### Data collection

Similarity searches were performed at the NCBI non redundant database (http://www.ncbi.nlm.nih.gov/BLAST/) using the HsMAB21L1 and HsD1A protein sequences as queries. psi-BLAST [38], and tBLASTn [39] programs were used with default parameters, except that filter was used for the lookup table only. The NCBI fungal and protists protein databases were scanned for fruitfly MAB-21 related proteins using psi-BLAST program with default parameters, except that filter was used for the lookup table only. The genome and proteome of *Xenopus tropicalis* (http://genome.jgi-psf.org/Xentr4/Xentr4.home.html) (v. 4.1) were searched for DANGER sequences using tBLASTn and BLASTp programs respectively (default parameters). Complete genomic sequences, segments of unfinished high throughput genomic sequences, and scaffolds were used for gene prediction utilizing the GenomeScan web server (http://genes.mit.edu/genomescan.html) [40]. The proteomes of *Rattus norvegicus*, *Danio rerio*, and *Ciona intestinalis* at Ensembl database (http://www.ensembl.org/) were searched for DANGER proteins using BLASTp program (default parameters). The proteome of *Nematostella vectensis* (http://genome.jgi-psf.org/Nemve1/Nemve1.home.html) (v. 1.0) was searched for DANGER sequences using BLASTp program (default parameters).

BLAST searches were conducted by using high expected E-values (*E* = 10) to ensure that most sequences homologous to the hD1A and hMAB21L1 would be retrieved. The resulted BLAST hits were sorted according to the E-value and all pairwise alignments were manually inspected. An initial phylogenetic analysis was performed in order to determine which sequences would be retained. Referenced (supported by cDNA sequences) or predicted amino acid sequences were aligned using ClustalX program [41]. Pairwise and multiple alignments were performed using BLOSUM protein weight series matrices, with default gap opening and gap extension penalties. Distance-based (*p*-distance) phylogenetic analysis was performed using the neighbor-joining (NJ) algorithm [42] as implemented in MEGA3 program [43]. Only sequences that unambiguously clustered within a particular DANGER family were retained. Accession numbers of all sequences used in this study are listed in table S1. Although, we cannot formally exclude the possibility that the structure of some genes may have been mispredicted, most human and mouse DANGER gene structures are supported by cDNA sequences.

Annotated genomic regions for the human, mouse, zebrafish, fly and nematode DANGER sequences were collected from the NCBI and Ensembl databases.

### Multiple sequence alignments

Referenced or predicted amino acid sequences were aligned using ClustalX program [41]. Pairwise and multiple alignments were performed using BLOSUM protein weight series matrices, with default gap opening and gap extension penalties. The multiple sequence alignments were manually edited. The alignment used to generate Figure 1 is shown in Figure S3. We need to note here that the accuracy of the alignments especially at the N-and C-termini could be affected by retained introns and missing exons.

### Phylogenetic analysis

Distance-based phylogenetic analyses were performed using the neighbor-joining (NJ) algorithm [42] as implemented in MEGA3 program [43]. *p*-distances (Figure S2) and poisson correction distances (Figure 1A) following the gamma distribution (α = 6.46) were used to construct the phylogenetic trees. The alpha parameter for rate variation among sites was estimated by the maximum-likelihood method as implemented in the TREE PUZZLE program [44]. The accuracy of the reconstructed trees was examined by the bootstrap test with 1,000 replications.

Unweighted maximum-parsimony (MP) reconstructions were performed using the protpars program of PHYLIP v.3.65 package. The MP analysis was performed with a heuristic search of 100 bootstrap replicates (datasets) and randomization of sequence order. MP analysis provided a single most parsimonious tree per dataset requiring a total of 17,300–18,300 steps. A consensus tree was constructed with the program consense, included in PHYLIP, using the extended majority rule criterion.

Maximum likelihood (ML) analyses were performed with TreePuzzle. The ML analysis was performed by using the quartet puzzling tree search procedure, with 25,000 puzzling steps. We used the Jones-Taylor-Thornton (JTT) model of substitution, with the frequencies of amino acids being estimated from the data set, and rate heterogeneity across sites modeled in eight rate categories. Quartet puzzling is only an approximation of the ML method [45] and is not immune to artifacts [46]. In addition, ML estimation with bootstrap application is computationally impracticable in the present case because of the size of the data set. For this reason we performed ML analysis as the method is implemented in PHYLIP v.3.65 by analyzing one dataset and using JTT model of substitution, gamma-distributed distances with five categories of HMM rates, global rearrangements of branching order, and randomization of sequence order. The resultant tree was essentially the same as the NJ tree, with respect to the major branching patterns (data not shown).

MP and ML analyses were performed on the LION-XM PC cluster run by the High Performance Computing Group of Penn State's Academic Services and Emerging Technologies (http://gears.aset.psu.edu/hpc/systems/lionxm/).

The phylogenetic relationships among paralogous DANGER sequences and for some anthozoan sequences remain unclear (low bootstrap support and differences in topologies using different methods). Several approaches were used to resolve these relationships. First, different regions of the proteins were used for phylogenetic reconstruction (i.e. only the conserved C-terminus regions of the protein sequences). Second, pairwise deletion of gaps was used to maximize the number of sites used for phylogenetic reconstruction. And third, different datasets containing only representative sequences of the different DANGER groups were used (for example, only vertebrate sequences). These different approaches gave essentially identical results in the phylogenetic analyses.

### *p*-distance calculations

Mean *p*-distances for sliding window and microdomain analyses were calculated using MEGA3 (complete deletion of gaps). Standard errors for *p*-distances were calculated using 500 bootstrap replications.

### GDDA analysis

GDDA analysis was conducted using our software as previously described [11], [12]. Briefly, GDDA modifies the original target sequence by inserting a proportion of the domain sequence (10–50%) (“seed”) at every amino acid position of the target sequence. The modified sequences are searched by rps-BLAST against the “seed” domain sequence and the percentages of coverage are plotted against each amino acid position. The modification of the original sequence increases the sensitivity of rps-BLAST search by providing a “constant” initiation sequence, the “seed”. This procedure allows BLAST to extent (i.e. ‘filling in the gaps’ hence gestalt) the alignment even between highly divergent sequences.

### Domain architecture analysis

The Mab-21 domain used in our domain architecture analysis has originally been defined in the Pfam database [47], and was imported to the Conserved Domain Database (CDD) at NCBI (http://www.ncbi.nlm.nih.gov/Structure/cdd/cdd.shtml) [48]. Domain architecture analysis of all referenced or predicted DANGER protein sequences was performed by using the executable local version of the rps-BLAST program (BLAST suite version: 2.2.14) and its accompanied CDD database. All searches were performed on the LION-XM PC cluster. The CDD position specific scoring matrix (PSSM) for Mab-21 domain was formatted as database using the formatrpsdb program (BLAST utility in the BLAST suite) and parameters set as described in ftp://ftp.ncbi.nih.gov/pub/mmdb/cdd/README file. This formatted PSSM was used as one entry database and all collected proteins as queries. Small size databases are shown to allow more sensitive searches, which are computationally inexpensive [49]. Results were filtered by setting an *E*-value threshold at 0.001 and stored as both pairwise alignments (-m option set at 0), or tabular results (-m option set at 8) [49]. Quantification of the Mab-21 domain coverage was performed by using the tabular results and applying the following formula:
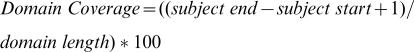
Subject start and end correspond to the Mab-21 domain coordinates on the pairwise alignments. The amino acid length of the Mab-21 domain PSSM consensus sequence equals to 360.

Mab-21 domain PSSM was built in CDD by using only four highly conserved MAB-21 protein sequences (*C. elegans* MAB-21, *D. melanogaster* MAB21a and MAB21b, and *M. musculus* MAB21L1; data from CDD FASTA files as provided at ftp://ftp.ncbi.nih.gov/pub/mmdb/cdd/). We built a new Mab-21 domain PSSM by utilizing the stand alone version of psi-BLAST. All D1–6 animal sequences (table S1), except the anthozoan sequences, were formatted as database using the formatdb BLAST utility with default parameters. HsMAB21L1 was used as query in an iterated psi-BLAST search using all D1–6 animal sequences (table S1), except the anthozoan sequences, as database. A new Mab-21 PSSM was built during the psi-BLAST searches utilizing the –C option of the stand alone psi-BLAST version. This psi-BLAST-generated Mab-21 PSSM was formatted for rps-BLAST searches and used as database for Mab-21 domain predictions. rps-BLAST searches were performed as in the case of original CDD Mab-21 PSSM. Quantification of the Mab-21 domain coverage was performed using formula (1) by modifying the size of the consensus psi-BLAST-generated Mab-21 domain sequence to 359.

Signal peptide sequences were predicted using the SignalP v. 3.0 server (http://www.cbs.dtu.dk/services/SignalP/) [50]. Transmembrane regions were predicted using the TMHMM v. 2.0 server (http://www.cbs.dtu.dk/services/TMHMM-2.0/) [51].

### Conserved synteny

The flanking genes of each DANGER gene were identified from the already annotated genome sequences (NCBI and Ensembl databases). In cases in which the flanking genes were not fully annotated similarity searches were performed using BLASTP (with *E* value threshold equal to 10^−10^) to identify homologs in other species.

### Genomic alignments

Approximately 25 Kbp upstream and downstream of each DANGER gene were retrieved from NCBI and Ensembl databases. Alignments among DANGER genomic sequences were performed using the BLASTZ [52] tool as implemented in the zPicture web tool (http://zpicture.dcode.org) [53]. BLASTZ generates sequence alignments between a reference sequence (first) and one or more other sequences. BLASTZ is a local alignment tool and identifies matches independent of their linear organization in the input sequences. Thus, zPicture identifies aligned regions independent of their location and orientation in the second sequence and maps these regions onto the reference sequence. In zPicture, BLASTZ alignments are visualized as standard percent identity plots (pip) [54].

### Transposable elements identification and annotation

To identify and annotate the transposable elements in the genomic regions of DANGER sequences the repeatmasker tool (default parameters) was employed (www.repeatmasker.org).

### Other computational tools

Graphs were generated with Microsoft Excel program. Statical tests were performed using MINITAB student 12 program. Domain architecture visualization was performed using the structure drawing tool (http://warta.bio.psu.edu/cgi-bin/Tools/StrDraw.pl). MSAs in block format were generated using WAVIS server (http://wavis.img.cas.cz) [55]. Sequence conservation patterns (logos) were generated using the WebLogo server (http://weblogo.berkeley.edu/) [56].

### Cell lines

PC12 cells were cultured in Dulbecco's Minimal Essential Medium supplemented with 10% horse serum, 5% fetal bovine serum, 2 mM L-glutamine and 1% penicillin-streptomycin. TT cells were obtained from ATCC and were cultured in RPMI 1640 supplemented with 15% fetal bovine serum, 1 mM glutamine and 1% penicillin-streptomycin. Embryonic spinal cord neurons were cultured and plated as previously described [57].

### RNA Sequences for DANGER1A

Two siRNA sequences were used for DANGER1A deletion, each with similar efficacy.

5′ aagaatgccccagcgctcatt 3′ Human

5′ aatacgagtttgaccttgctt 3′ Rat

### Expression protocols

Transfection of PC12, primary mouse spinal cord neurons, and TT cells with either 100 nM siRNA duplex and 1 µg YFP, or 1 µg DANGER1A and 1 µg YFP, was performed using Lipofectamine 2000 (Invitrogen), according to the manufacturer's specifications.

### Antibodies and reagents

Plasmids were from the following sources: EYFP, Matchmaker© and Myc-CMV vector cDNA from CLONETECH; and human M5 muscarinic receptor cDNA from L. Birnbaumer (NIH). Human Northern Blot from CLONTECH (Palo Alto, CA). Carbachol, Protein A agarose, GST agarose, Sigma (St. Louis, MO). siRNA duplex was from Qiagen (Valencia, CA). TrkA, p75, phosphor-TrkA, and phospho-p75 antibodies from Upstate Biotech (Charlottesville, VA). Antibodies were used as to the manufacturers instructions.

### Subcellular fractionation

After inducing differentiation by NGF, cells were harvested by gently scraping plates with a cell scraper, and were washed once with cold PBS. The washed pellet was subjected to one freeze-thaw cycle in liquid nitrogen. Pellets were resuspended in 1 ml Buffer A (250 mM sucrose, 10 mM Tris-HCl [pH 7.5], 1 mM EGTA, 1mM PMSF, one protease inhibitor pill). Cells were then homogenized on ice using a 1 ml glass Dounce homogenizer with a tight fitting pestle until ∼95% of cells were disrupted as judged by Trypan blue staining. Crude lysates were centrifuged at 1,000× g for 15 min at 4°C to remove nuclei and unbroken cells. The supernatant was collected and the pellet (P1) discarded. The low speed supernatant was then subjected to 10,000× g centrifugation for 15 min, which yielded the 10,000× g pellet (P2). The supernatant from the P2 pellet was centrifuged at 15,000× g to completely rid the supernatant of any remaining mitochondria. Finally the 15,000× g supernatant was separated into cytosol (S3) and light membrane (P3) fractions by centrifugation at 100,000× g for 1 h. The 100,000× g supernatant was collected as the S3 fraction and the pellet was resuspended in 40–70 µl of Buffer A. P2 was washed twice by resuspending cells in 100 µl Buffer A and pelleting (10,000× g for 15 min). After the final wash, P2 was resuspended in 50–100 µl Buffer A. All fractionations were repeated a minimum of four times with essentially identical results. Subcellular fractions were characterized using nucleoporin (P1- nuclear), cytochrome c oxidase (P2- mitochondria, heavy ER), heme oxygenase 2 (P3- light ER), and lactate dehydrogenase (S3- cytosol).

### Immunofluorescence

Primary spinal cord neurons were cultured on coverslips for 10 days then fixed with 0.4% paraformaldehyde in 3× PBS for 30 min and washed three times in 3× PBS for 10 min. Coverslips were quenched in 50 mM NH_4_Cl for 15 min and washed three times in 2× PBS for 10 min. Cells were permeabilized in saponin solution (3× PBS, 1% bovine serum albumin, 1% goat serum, and 0.075% w/v saponin) for 1 hr. Coverslips were washed three times in 3× PBS for 10 min and subsequently inverted on 250 µl anti-DANGER1a conjugated to Alexa 488© fluorophores from Molecular Probes in a wet chamber overnight at 4°C. Coverslips were washed three times in 3× PBS for 10 and mounted onto slides with Slo-fade©. Fluorescence signals were detected with a Zeiss LSM410 confocal laser scanning microscope, using a 63x/NA 1.4 objective.

### Immunohistochemistry

Formalin fixed, paraffin embedded 5 µM sections were dewaxed, rehydrated and subjected to antigen retrieval by incubation in 10mM sodium citrate pH 6.0 at 98°C for 20 mins. Sections were then immunostained using the Vectastain^TM^ ABC system according to the manufacturer instructions using affinity purified anti-DANGER antibody described above at a dilution of 1/50.

## Supporting Information

Figure S1 GDDA analysis of the mouse DANGER1A protein reveals the presence of an almost complete Mab-21 domain. (A) rps-BLAST similarity search of the MmD1A protein with E-value threshold equal to 0.01 and no filter for low complexity regions does not predict any domain on the query (MmD1A) sequence. (B) GDDA analysis with 10–50% “seeds” of the N-terminus of the Mab-21 domain consensus sequence, as defined in the Conserved Domain Database, identifies almost 90% of the Mab-21 domain sequence in MmD1A.
(0.07 MB PDF)

Figure S2 Phylogenetic relationships of the DANGER superfamily including all identified anthozoan (*Nv, Nematostella vectensis*) sequences. The tree was constructed with the NJ method using *p*-distances for 206 amino acid sites after elimination of alignment gaps. The *p*-distances are known to give a higher resolution of branching pattern because of the smaller standard errors. Numbers at branches represent bootstrap values. Species in red fonts denote the presence of introns in their corresponding DANGER coding sequence, while species in black fonts denote the absence of introns in their corresponding DANGER coding sequence.
(0.02 MB PDF)

Figure S3 Multiple sequence alignment of the DANGER superfamily used to generate the phylogenetic tree of Fig. 1A.
(0.69 MB PDF)

Figure S4 Plots of the informative parameters for the rps-BLAST pairwise alignments between the Mab-21 domain profile (Pfam or psi-BLAST-generated) and the human DANGER proteins. (A) Mab-21 domain coverage, (B) sequence identity, (C) sequence similarity, (D) proportion of gaps, and (E) alignment bit score. Use of the psi-BLAST-generated Mab-21 profile results in increased domain coverage and alignment bit score; sequence identity and sequence similarity are increased for DANGER 5 and 6 groups, while for the remaining DANGER groups are decreased. The proportion of gaps in groups DANGER 1–3, and 5 is also increased.
(0.06 MB PDF)

Figure S5 Plots of the informative parameters for the rps-BLAST pairwise alignments between Mab-21 domain profiles (Pfam or psi-BLAST-generated) and choanoflagellate, anthozoan, arthropod, echinoderm, and urochordate DANGER proteins. (A) Mab-21 domain coverage, (B) sequence identity, (C) sequence similarity, (D) proportion of gaps, and (E) alignment bit score.
(0.07 MB PDF)

Figure S6 Graphical representation of mean *p*-distances in the DANGER superfamily. (A) The graph depicts the mean *p*-distances per DANGER family within each Mab-21 micro-domain (II–IX). (B) The graph shows the mean *p*-distances per Mab-21 micro-domain for each DANGER family. Bars represent standard errors.
(0.07 MB PDF)

Figure S7 Pattern of sequence conservation (logo) along the less conserved Mab-21 micro-domains. (A) micro-domain II. (B) micro-domain III. (C) micro-domain IV. (D) micro-domain V. (E) micro-domain IX. Y axis represents the amount of information present at every amino acid position in the sequence, measured in bits.
(1.78 MB PDF)

Figure S8 Comparison of pairwise alignments between the psi-BLAST-generated Mab-21 profile and the anthozoan DANGER sequences reveals conservation of insertions (black boxes) and deletions (white boxes), and correspondence of indels (>3 amino acids) with intron positions (yellow boxes). For clarity, all sequences are mapped onto the Mab-21 profile sequence according to the pairwise alignment coordinates. Also, the pairwise alignments for *D. melanogaster* and *S. purpuratus* proteins are shown. Nv, *N. vectensis*; DmD4, CG7194; Dm4, CG15865; Sp2, XP_794693.
(0.01 MB PDF)

Figure S9 The region 230–258 of the block formatted pairwise alignments shown in Figure 3 is magnified to demonstrate the positional conservation of insertions (magenta fonts) among the DANGER proteins. The amino acids in yellow bold fonts correspond to exon boundaries at the nucleotide level. Hs, *H. sapiens*; Dm, *D. melanogaster*; Ce, *C. elegans*; Nv, *N. vectensis*.
(0.13 MB PDF)

Figure S10 DANGER genes are in conserved synteny among vertebrates (D1–D5) or metazoa (D6). Genes are depicted as pentagon arrows to show transcription orientation. (A) D6 family members are in conserved synteny among all metazoan taxa used in this study, except for *D. melanogaster*. In particular, the MAB21L1 (L1) genes are located in an intron of the neurobeachin (NBEA) gene and the MAB21L2 (L2) genes are located in an intron of the LPS-responsive vesicle trafficking, beach and anchor containing (LRBA) gene. In data not shown, phylogenetic analysis of the NBEA and LRBA genes from vertebrates, and invertebrates suggests that these genes are homologous and have been duplicated in vertebrates like the L1 and L2 genes. These data suggest that the L1 and L2 genes are products of *en block* duplication in vertebrates. The coding sequence of D6 family members from *N. vectensis*, and vertebrates is not interrupted by introns, while the orthologous sequences from ecdysozoa (*D. melanogaster* and *C. elegans*), *C. intestinalis* and *S. purpuratus* contain introns (see Fig. 3A, 4A, and Figure S2). In the *D. melanogaster* genome the D6 gene is duplicated and both copies are located on chromosome X, region 5D1-D2, approximately 800 kb downstream of the fly neurobeachin homolog (chromosome X, region 4F3-4F3). This duplication seems to have occurred in all insects, since *Anopheles gambiae*, *Apis mellifera*, *Tribolium castaneum*, and *Bombyx mori* all contain two copies of the D6 gene (data not shown). (B) D1A genes are in conserved synteny among vertebrates. *D. rerio* genome contains two copies of the D1A gene. (C) D1B genes are in conserved synteny among mammals. Our exhaustive similarity searches did not reveal D1B orthologs from other vertebrate species; here we show that the *X. tropicalis* syntenic region bears NUP133 gene in the syntenic position of the mammalian D1B gene. (D) D1C genes are in conserved synteny among warm-blooded animals. (E) D4 family members are in conserved synteny among vertebrates. (F) D5 family members are in conserved synteny among vertebrates. (G) D2A genes are in conserved synteny among vertebrates. D. rerio genome contains two copies of the D2A gene. (H) D3A genes are in conserved synteny among tetrapods. (I) D3B genes are in conserved synteny among tetrapods. (J) D2B genes are in conserved synteny among mammals. Our exhaustive similarity searches did not reveal D2B orthologs in non-mammalian vertebrates. Gene abbreviations: GSTO1, glutathione S-transferase omega 1; GSTO2, glutathione S-transferase omega 2; TMEM127, transmembrane protein 127; WDR39, WD repeat domain 39; ASCC3L1, activating signal cointegrator 1 complex subunit 3-like 1; NCAPH, non-SMC condensin I complex, subunit H; TMC7, transmembrane channel-like gene 7; COQ7, coenzyme Q7 homolog, ubiquinone (yeast); SYT17, synaptotagmin XVII; DDX43, DEAD (Asp-Glu-Ala-Asp) box polypeptide 43; MTO1, mitochondrial translation optimization 1 homolog (*S. cerevisiae*); EEF1A1, eukaryotic translation elongation factor 1 alpha 1; ATP1A1, ATPase type 1A1; FGF12, fibroblast growth factor 12; HRASLS, HRAS-like suppressor; ATP13A5, ATPase type 13A5; ATP13A4, ATPase type 13A4; ATF4, activating transcription factor 4 (tax-responsive enhancer element B67); LLGL1, lethal giant larvae homolog 1 (*Drosophila*); FLII, flightless I homolog (*Drosophila*); TOP3A, topoisomerase (DNA) III alpha; SMCR8, Smith-Magenis syndrome chromosome region, candidate 8; FGF11, fibroblast growth factor 11.
(0.07 MB PDF)

Figure S11 zPicture visualization of representative alignments among DANGER genomic sequences. zPicture uses the local alignments tool BLASTZ to generate sequence alignments between a reference sequence (the first one) and one or more sequences. BLASTZ identifies matches independent of their linear organization in the input sequences and zPicture maps these alignments onto the reference (first) sequence. Local alignments are visualized as standard percent identity plots. (A) Visualizations of pairwise alignments among *H. sapiens* (h) DANGER paralogs. Note: When all sequences are masked for transposable elements (TEs) no similarity is found, except between HsD1A–HsD1B coding sequences. (B) Visualizations of pairwise alignments among *M. musculus* (m) DANGER paralogs. Note: When all sequences are masked for TEs no similarity is found, except between MmD1A–MmD1B coding sequences. (C) Visualizations of pairwise alignments among D. rerio (z) DANGER paralogs. Note: When all sequences are masked for TEs no similarity is found, except between DrD1A1–DrD1A2 coding sequences. (D) Upper: visualizations of pairwise alignments among D1A orthologous sequences from *H. sapiens*, *M. musculus*, and *D. rerio*. Lower: visualizations of pairwise alignments among D1A orthologous sequences from *H. sapiens*, *M. musculus*, and *D. rerio* after masking of TEs. (E) Upper: visualizations of pairwise alignments among MAB21L1 (L1) orthologous sequences from *H. sapiens*, *M. musculus*, and *D. rerio*. Lower: visualizations of pairwise alignments among L1 orthologous sequences from *H. sapiens*, *M. musculus*, and *D. rerio* after masking of TEs. (F) Upper: visualization of pairwise alignments between L1 and L2 paralogous sequences. Lower: Visualizations of pairwise alignments between L1 and L2 paralogous sequences, after masking of TEs. (G) Visualizations of pairwise alignments between HsD2B and HsD2A genomic regions. (H) Visualizations of pairwise alignments between HsD3A and HsBCR genomic regions. (I) Visualizations of pairwise alignments between HsD3B and HsBCR genomic regions. (J) Visualization of pairwise alignments between the genomic sequences of HsD1C and HsD1B using the zPicture web tool. (K) Upper: Visualization of pairwise alignments between the genomic sequences of HsD3A and HsD3B. Lower: Visualization of pairwise alignments between the genomic sequences of HsD3A and HsD3B after masking the transposable elements (TEs). (L) Visualization of pairwise alignments between the genomic sequences of HsD4 and HsBCR. DANGER genes are shown in blue boxes (exons) and blue lines (introns). Arrows show transcriptional orientation. Green boxes denote TEs.
(0.18 MB PDF)

Figure S12 Repeat content of the genomic regions flanking the DANGER genes (approximately 25 Kbp upstream and downstream of each DANGER gene were analyzed). (A) Average number of different repetitive element families in zebrafish, mouse, and human DANGER genomic regions. (B) Average number of different repetitive element families in each DANGER family. (C) Repeat content in the *D. rerio* DANGER genomic regions. (D) Repeat content in the mouse DANGER genomic regions. (E) Repeat content in the human DANGER genomic regions. DNA, DNA trasnposons; LINE, long-interspersed nucleotide elements; LC, low complexity regions; LTR, retrovirus-like elements with Long Terminal Repeats; SR, simple repeats; SINE, short-interspersed nucleotide elements; UN, unclassified repeats.
(0.09 MB PDF)

Figure S13 Analysis of expressed sequence tags (ESTs) show that mouse and human DANGER sequences exhibit different transcriptional patterns. (A) Total expression levels (sum of different tissues) for all *M. musculus* DANGER genes. (B) Expression of *M. musculus* DANGER genes in different tissues showed as transcripts per million. (C) Expression of *M. musculus* DANGER genes in different developmental stages. (D) Total expression levels (sum of different tissues) for *H. sapiens* DANGER genes. (E) Expression of *H. sapiens* DANGER genes in different tissues. (F) Expression of *H. sapiens* DANGER genes in different health states. EST data were collected from the UniGene database of NCBI (http://www.ncbi.nlm.nih.gov/entrez/query.fcgi?db = unigene). The UniGene identification number for each sequence is given below. For mouse: MmL1, Mm.384353; MmL2, Mm.389466; MmD5, Mm.32900; MmD4, Mm.101559; MmD3A, Mm.307163; MmD3B, Mm.339760; MmD2A, Mm.155887; MmD2B, Mm.280165; MmD1A, Mm.29457; MmD1B, Mm.57559; MmD1C, Mm.323386; and for human: HsD1A, Hs.523252; HsD1B, Hs.65009; HsD1C, Hs.530899; HsD4, Hs.14577; HsD3A, Hs.148677; HsD3B, Hs.551967; HsD2A, Hs.151443; HsL1, Hs.584776; HsL2, Hs.584852. Tissues and or stages are plotted if they contained more than 80,000 EST sequences. The presence of many ESTs demonstrates that DANGER sequences are expressed and thus DANGERs do not probably represent pseudogenes. The different expression patterns suggest putative functional divergence among paralogous DANGER genes supporting our phylogenetic analyses, which predicts that the different DANGER groups evolve following the model of divergent evolution.
(0.07 MB PDF)

Figure S14 DANGER1A is expressed in the brain and a variety of terminally differentiated tissues. (A) Random primers of HsD1A probed against a multiple tissue Northern Blot. (B–D) Immunohistochemistry on sagittal sections of E13.5 whole mouse, P0 brain and adult brain stained with polyclonal antibody against D1A, respectively. (E) Top: Horizontal brain section depicting the strong D1A staining in the CA1 of the hippocampus (arrow), and the lack of D1A staining in the dente gyrus (arrowhead). Bottom: Horizontal brain section depicting the strong D1A staining in the cerebellum. (F) Clockwise from left: anti-D1A staining in mouse pancreas, peripheral nerve, small intestine, cerebral cortex, skeletal muscle, and adrenal gland. The antibody used is specific to DANGER1a and is described in van Rossum et al. 2006 (Reference number 4 in main text). Antibody reactivity is indicated by the brown staining, while cells absent in DANGER expression are violet in color.
(1.22 MB PDF)

Figure S15 (A) Left: Western analysis of (20 µg) thyroid tumor (TT) cell or Brain lysates blotted with mouse monoclonal antibody against p75. Middle Left: Western analysis of (20 µg) PC12 or TT cell lysates blotted with polyclonal antibody against TrkA. Middle Right: Western analysis of (20 µg) TT cell lysate +/−50 ng/ml NGF for 48 h, blotted with monoclonal antibody against anti phospho-TrkA. GAPDH was used as a loading control. Right: Western analysis of (20 µg) PC12 cell or TT cell lysates blotted with polyclonal antibody against DANGER1A. PC12 cell express a major band (59 kDa) corresponding to the full-length protein and a second band (41 kDa), which is presumed to be a cleavage/breakdown product of D1A protein, since both bands are siRNA-sensitive. TT cells express only the 41 kDa band. Actin was used as a loading control. (B) YFP or YFP+DANGER1A transfected TT cells visualized by epifluorescent microscopy. Arrow denotes neurite growth.
(0.18 MB PDF)

Table S1 List of sequences used in this study
(0.01 MB PDF)

Table S2 Repetitive elements found in DANGER mRNA sequences. Numbers correspond to the mRNA coordinates.
(0.01 MB PDF)
