# Supplementary material for: Ancient Origin of the New Developmental Superfamily DANGER
Source: PLoS One. 2007 Feb 14;2(2):e204. doi: 10.1371/journal.pone.0000204 (PMC1784063; doi:10.1371/journal.pone.0000204)
Supplement: Figure S1 — GDDA analysis of the mouse DANGER1A protein reveals the presence of an almost complete Mab-21 domain. (A) rps-BLAST similarity search of the MmD1A protein with E-value threshold equal to 0.01 and no filter for low complexity regions does not predict any domain on the query (MmD1A) sequence. (B) GDDA analysis with 10–50% “seeds” of the N-terminus of the Mab-21 domain consensus sequence, as defined in the Conserved Domain Database, identifies almost 90% of the Mab-21 domain sequence in MmD1A. (0.07 MB PDF) [file pone.0000204.s001.pdf]

A Query: DANGER1A mouse

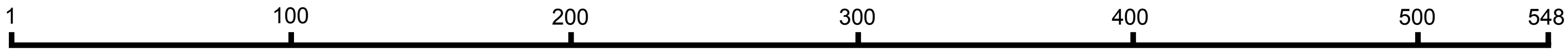

No conserved domain has been identified on this query sequence (rps-BLAST, E=0.01, no-filter)

B Predicted domain: GDDA

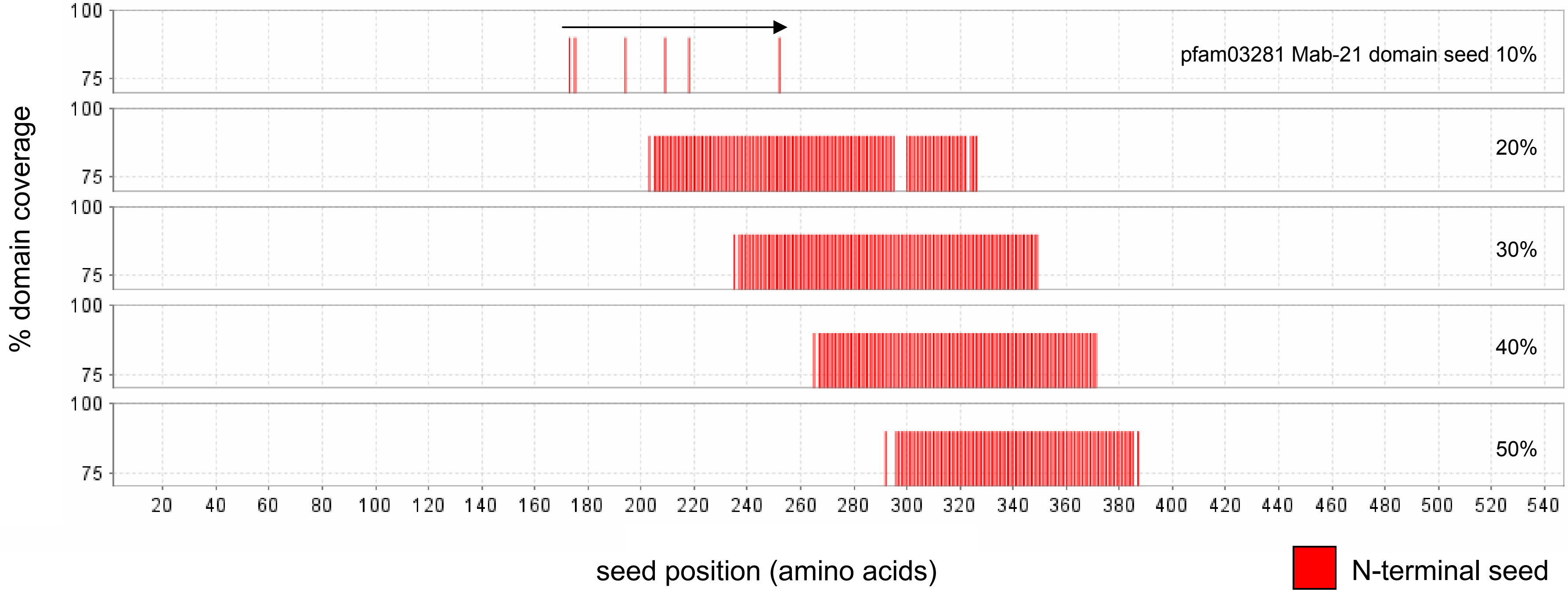

Nikolaidis et al. fig. S1
