# Supplementary figures and images for: Ancient Origin of the New Developmental Superfamily DANGER
Source: PLoS One. 2007 Feb 14;2(2):e204. doi: 10.1371/journal.pone.0000204 (PMC1784063; doi:10.1371/journal.pone.0000204)

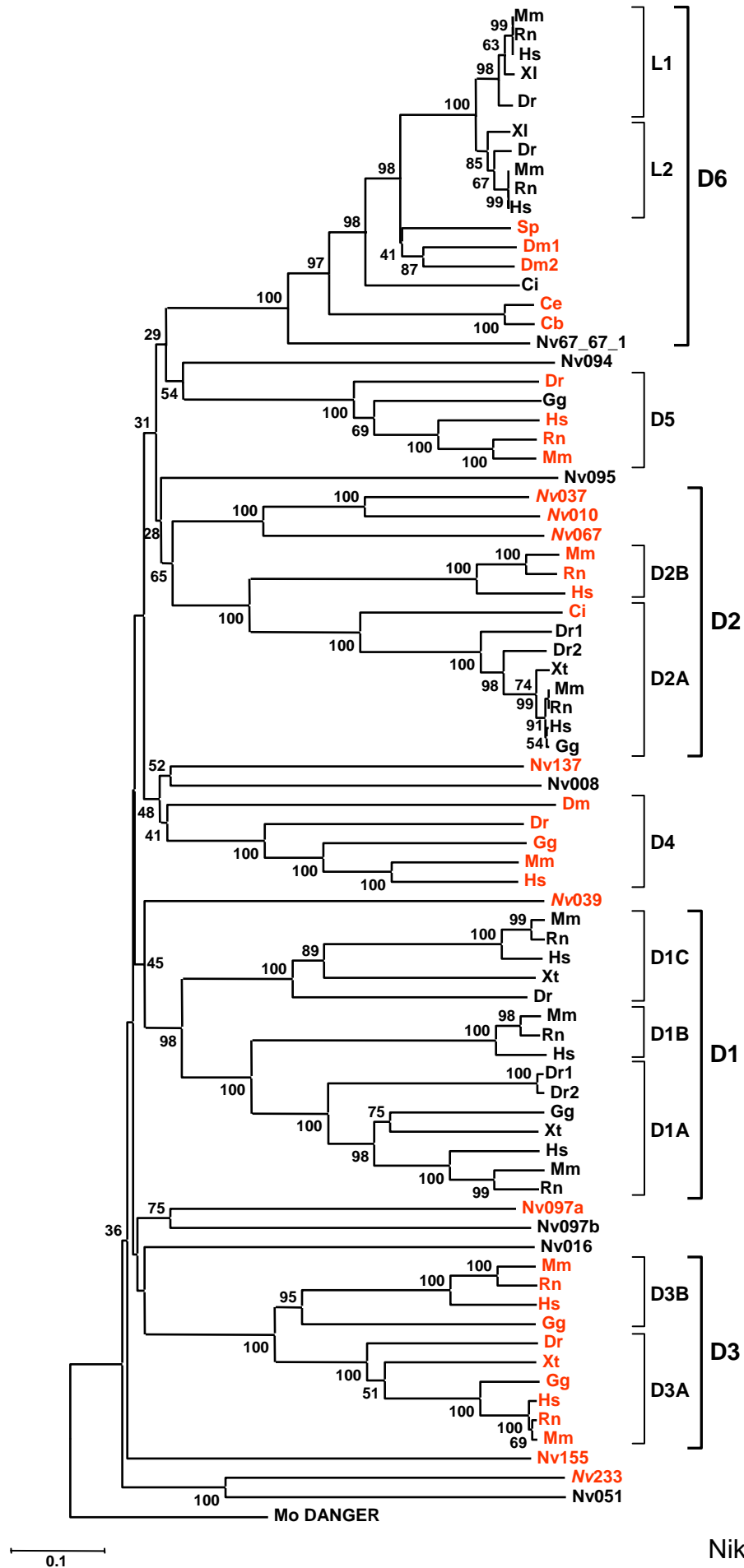

Supplement: Figure S2 — Phylogenetic relationships of the DANGER superfamily including all identified anthozoan (Nv, Nematostella vectensis) sequences. The tree was constructed with the NJ method using p-distances for 206 amino acid sites after elimination of alignment gaps. The p-distances are known to give a higher resolution of branching pattern because of the smaller standard errors. Numbers at branches represent bootstrap values. Species in red fonts denote the presence of introns in their corresponding DANGER coding sequence, while species in black fonts denote the absence of introns in their corresponding DANGER coding sequence. (0.02 MB PDF) [file pone.0000204.s002.pdf]

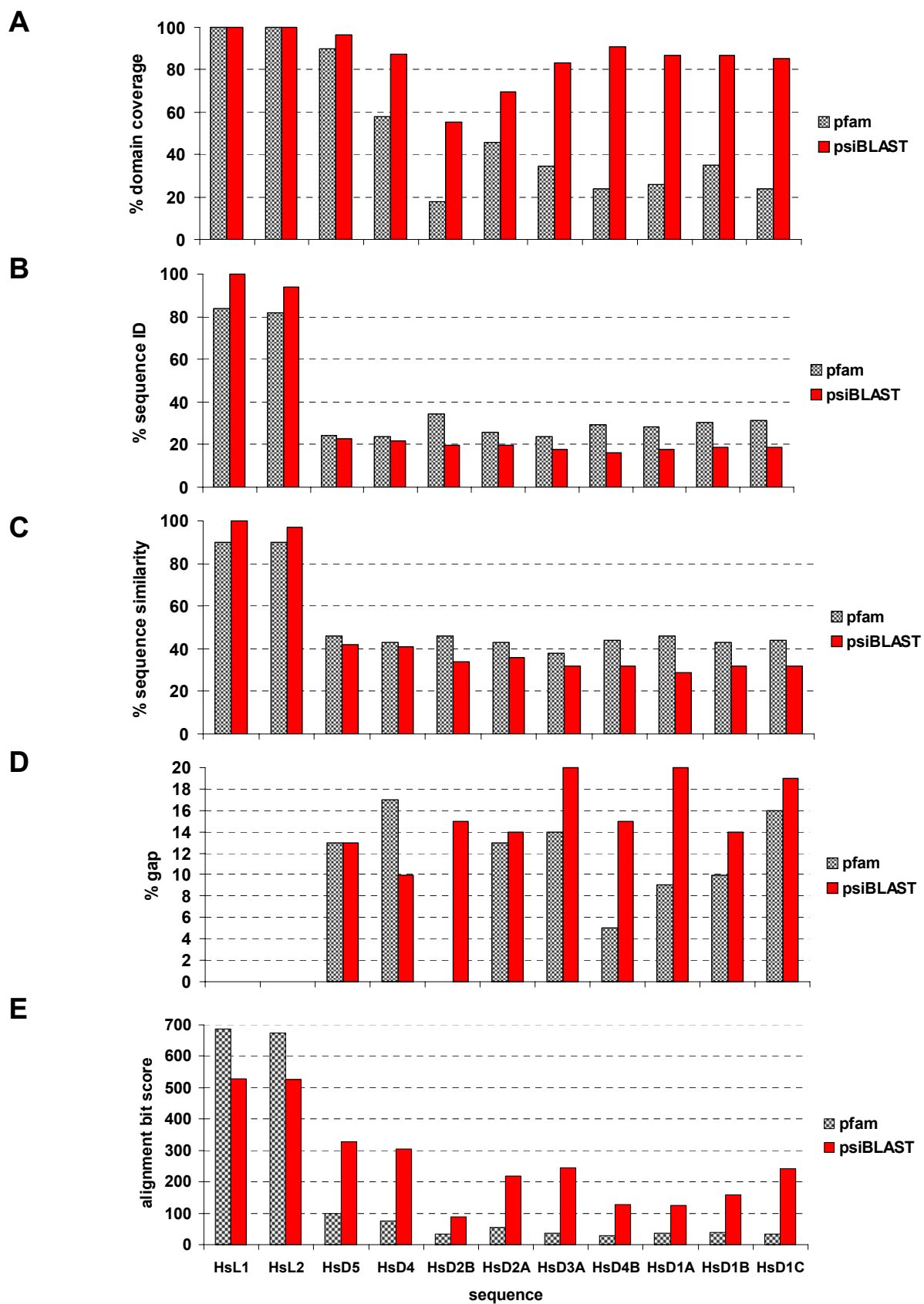

Supplement: Figure S4 — Plots of the informative parameters for the rps-BLAST pairwise alignments between the Mab-21 domain profile (Pfam or psi-BLAST-generated) and the human DANGER proteins. (A) Mab-21 domain coverage, (B) sequence identity, (C) sequence similarity, (D) proportion of gaps, and (E) alignment bit score. Use of the psi-BLAST-generated Mab-21 profile results in increased domain coverage and alignment bit score; sequence identity and sequence similarity are increased for DANGER 5 and 6 groups, while for the remaining DANGER groups are decreased. The proportion of gaps in groups DANGER 1–3, and 5 is also increased. (0.06 MB PDF) [file pone.0000204.s004.pdf]

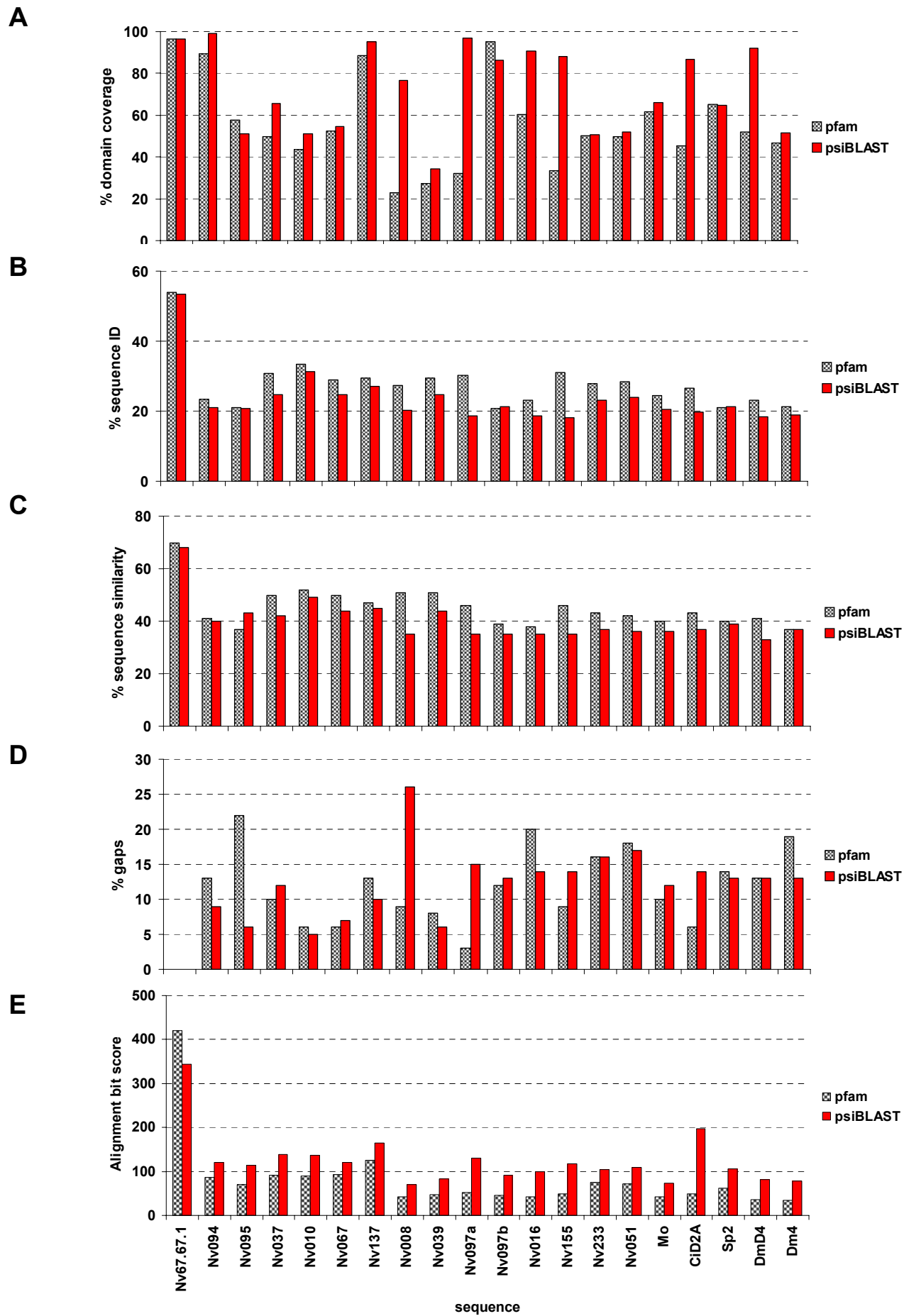

Supplement: Figure S5 — Plots of the informative parameters for the rps-BLAST pairwise alignments between Mab-21 domain profiles (Pfam or psi-BLAST-generated) and choanoflagellate, anthozoan, arthropod, echinoderm, and urochordate DANGER proteins. (A) Mab-21 domain coverage, (B) sequence identity, (C) sequence similarity, (D) proportion of gaps, and (E) alignment bit score. (0.07 MB PDF) [file pone.0000204.s005.pdf]

A

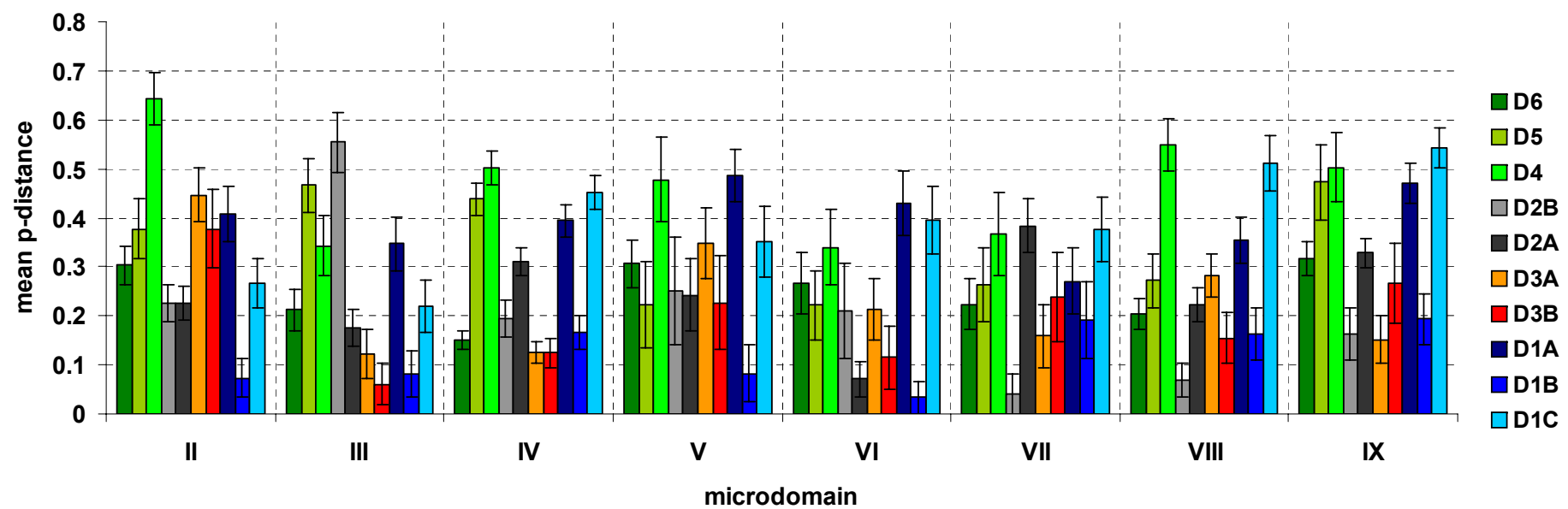

**B**

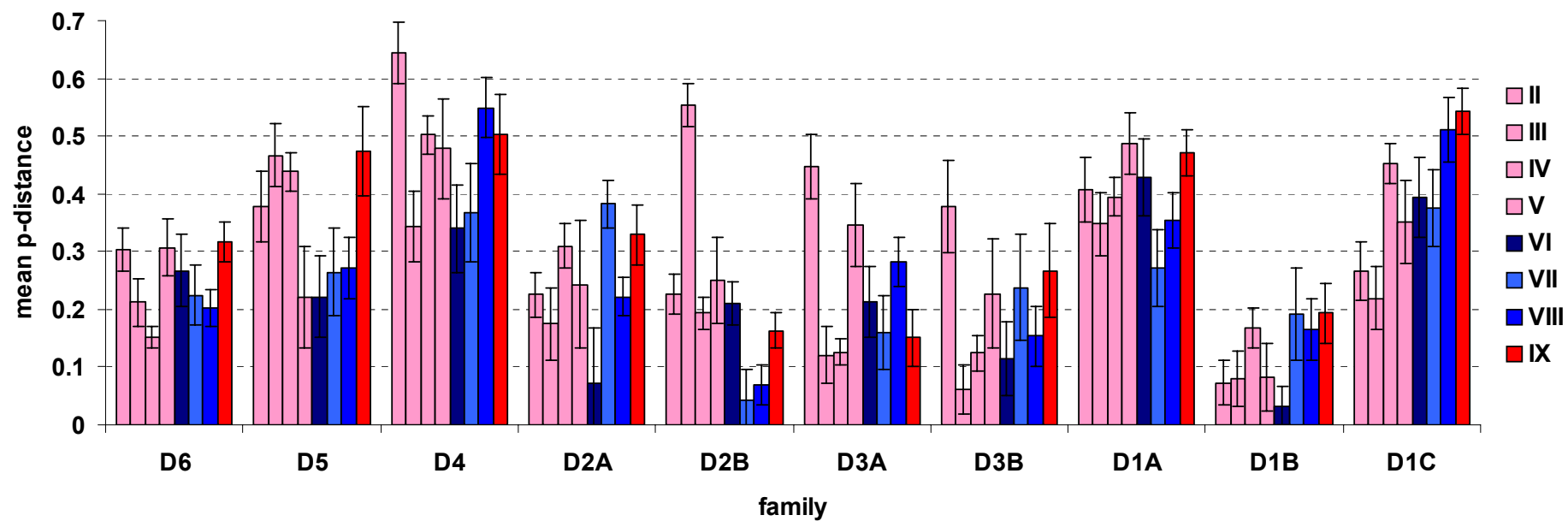

Supplement: Figure S6 — Graphical representation of mean p-distances in the DANGER superfamily. (A) The graph depicts the mean p-distances per DANGER family within each Mab-21 micro-domain (II–IX). (B) The graph shows the mean p-distances per Mab-21 micro-domain for each DANGER family. Bars represent standard errors. (0.07 MB PDF) [file pone.0000204.s006.pdf]

A

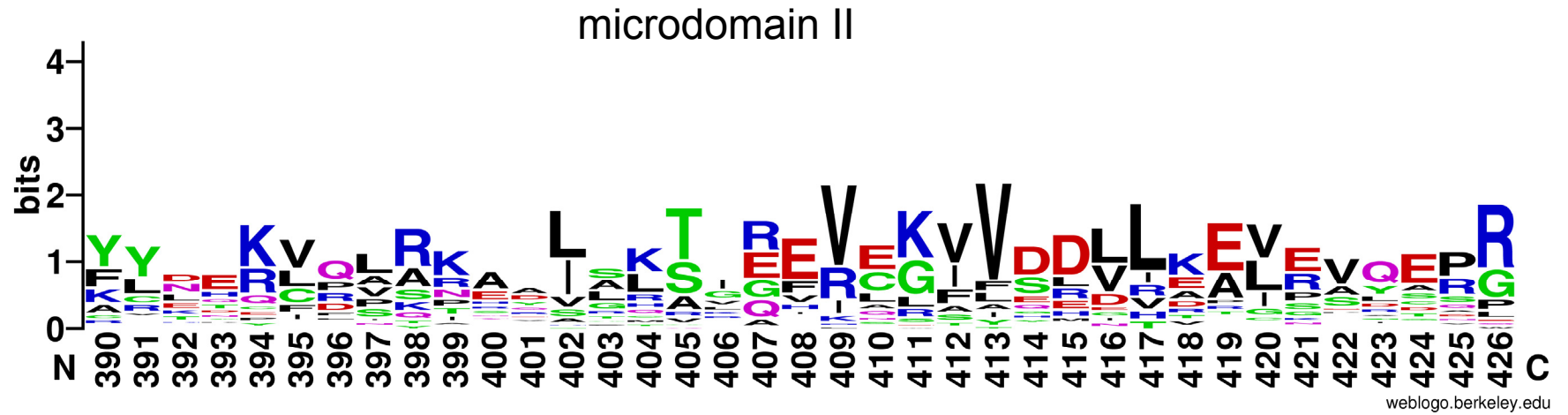

B

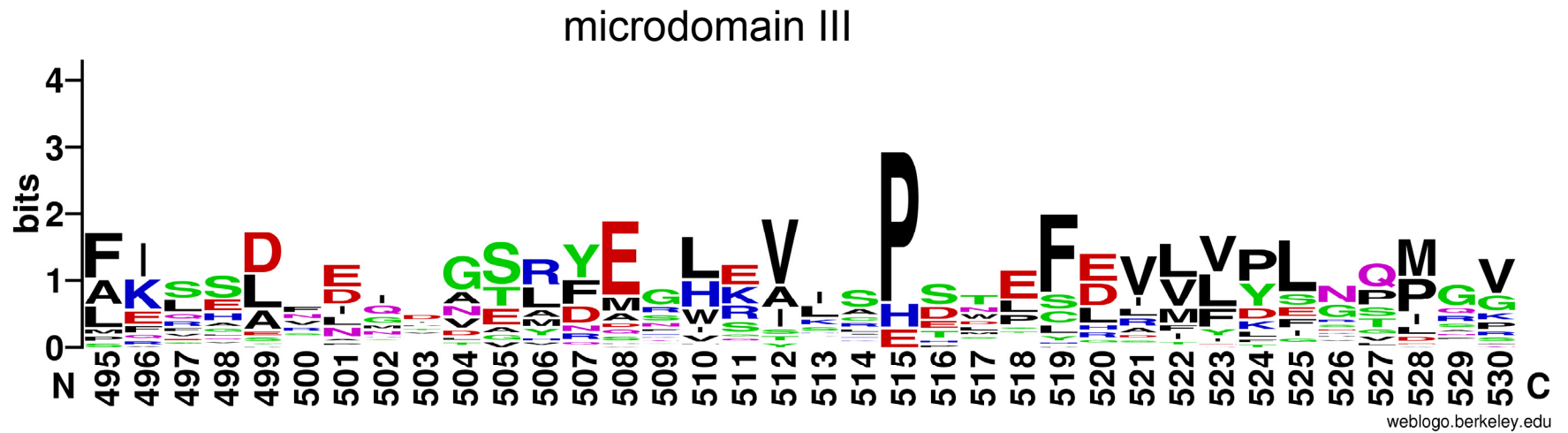

C

## microdomain IV

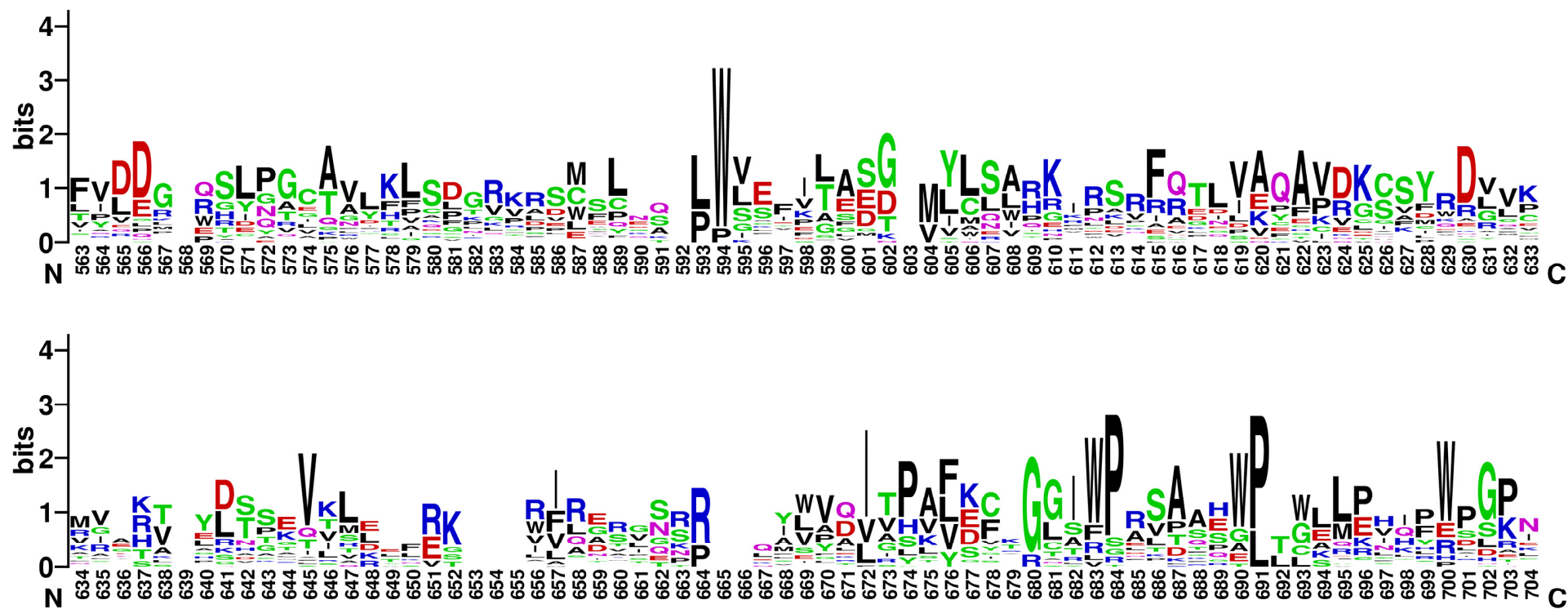

weblogo.berkeley.edu

D

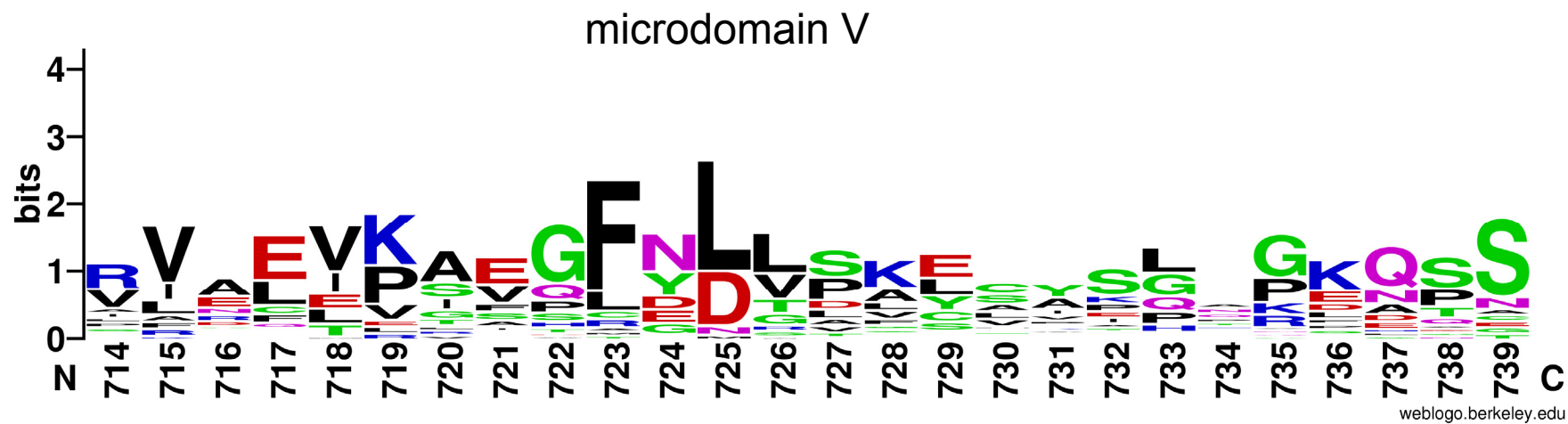

# E

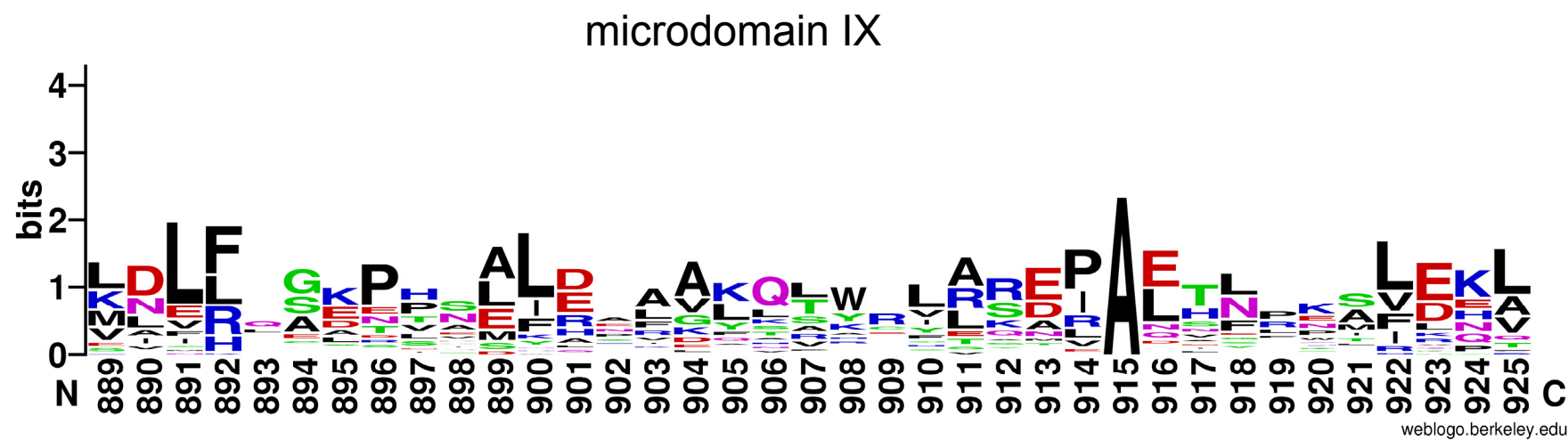

Supplement: Figure S7 — Pattern of sequence conservation (logo) along the less conserved Mab-21 micro-domains. (A) micro-domain II. (B) micro-domain III. (C) micro-domain IV. (D) micro-domain V. (E) micro-domain IX. Y axis represents the amount of information present at every amino acid position in the sequence, measured in bits. (1.78 MB PDF) [file pone.0000204.s007.pdf]

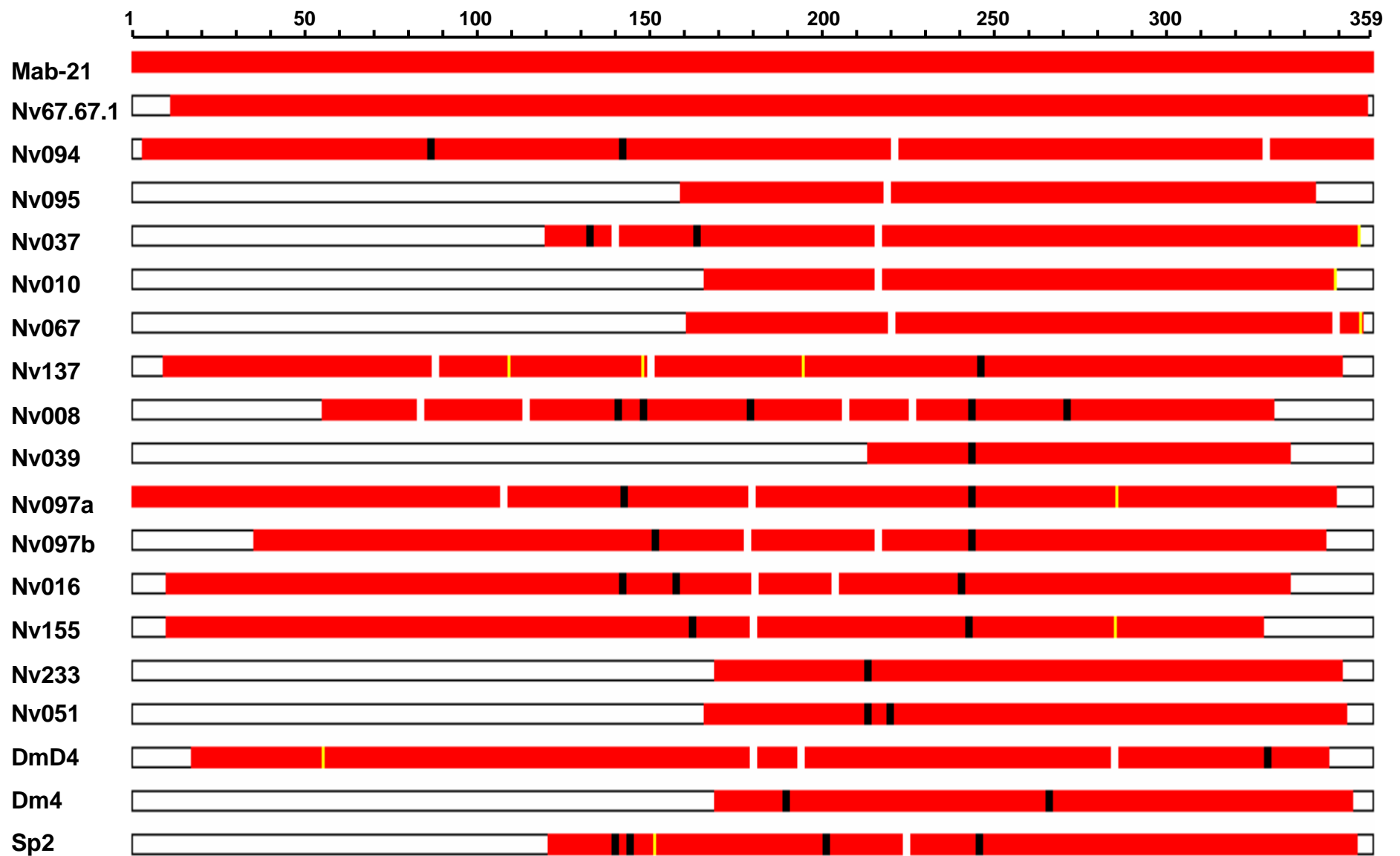

Supplement: Figure S8 — Comparison of pairwise alignments between the psi-BLAST-generated Mab-21 profile and the anthozoan DANGER sequences reveals conservation of insertions (black boxes) and deletions (white boxes), and correspondence of indels (>3 amino acids) with intron positions (yellow boxes). For clarity, all sequences are mapped onto the Mab-21 profile sequence according to the pairwise alignment coordinates. Also, the pairwise alignments for D. melanogaster and S. purpuratus proteins are shown. Nv, N. vectensis; DmD4, CG7194; Dm4, CG15865; Sp2, XP_794693. (0.01 MB PDF) [file pone.0000204.s008.pdf]

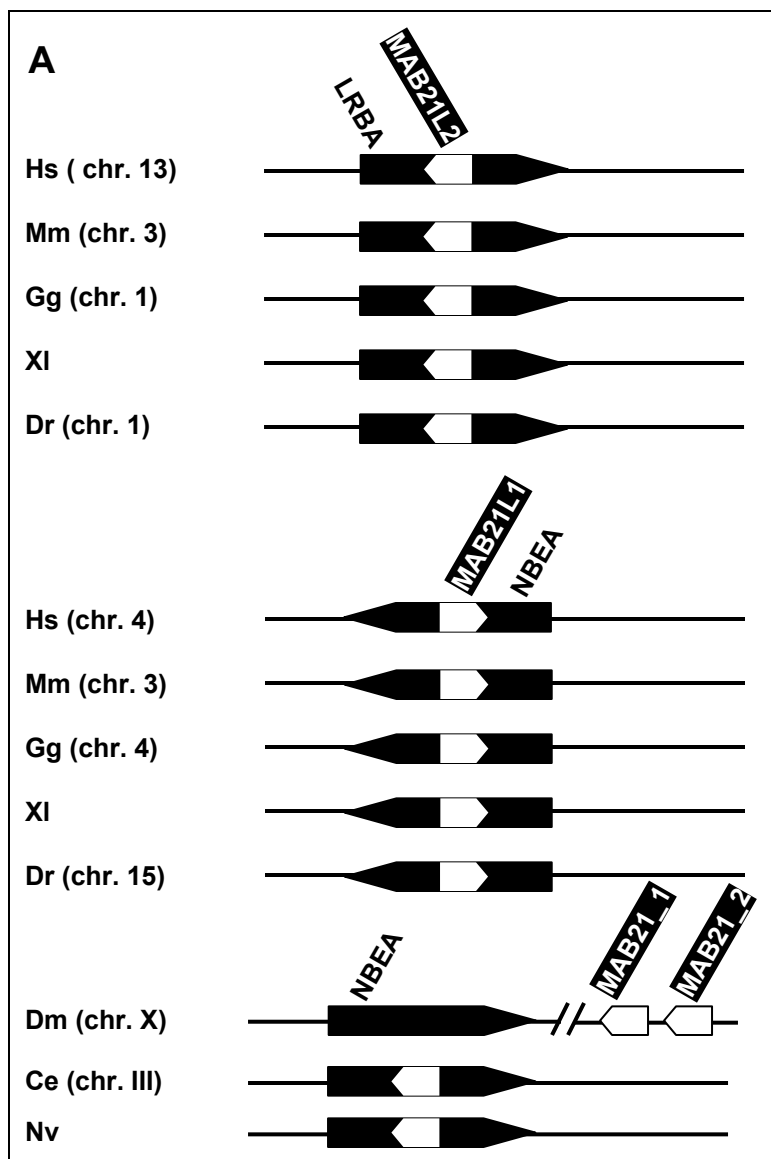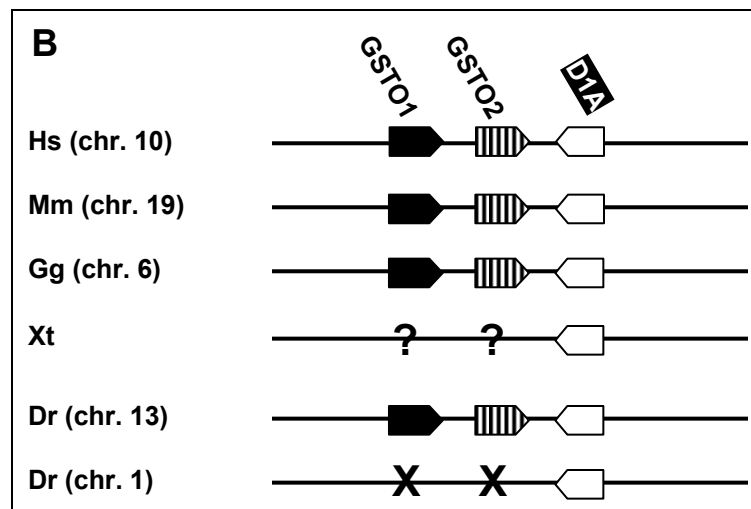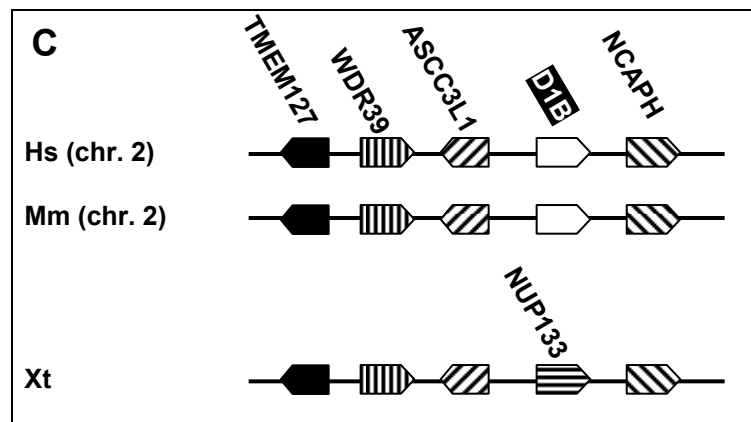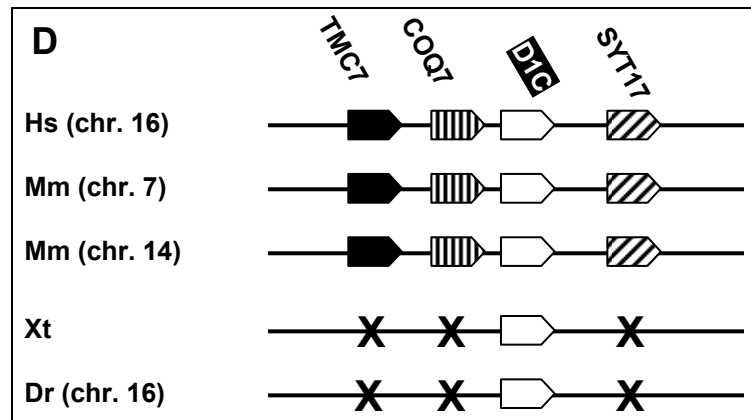

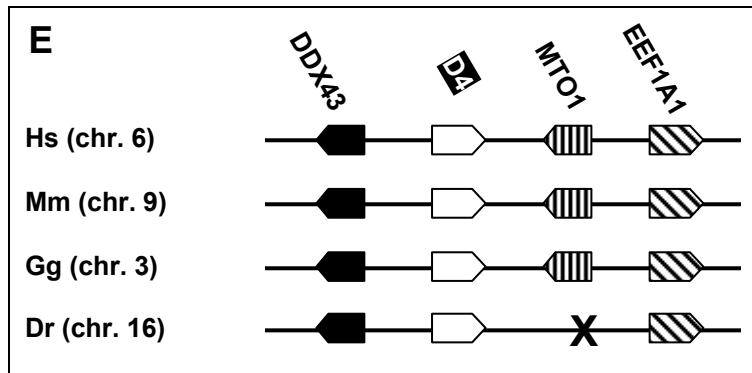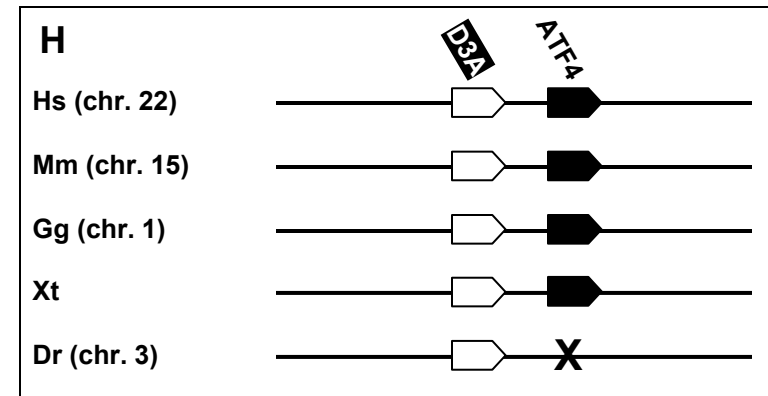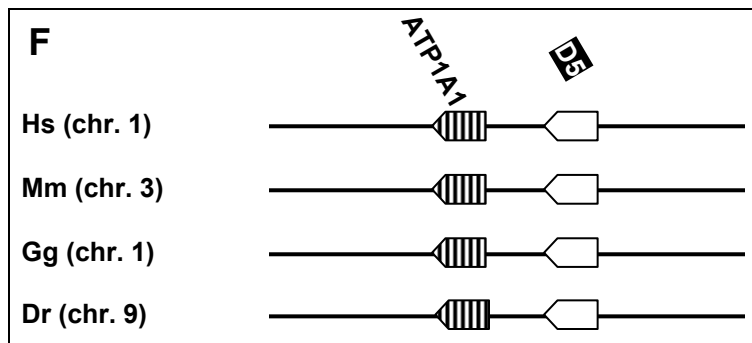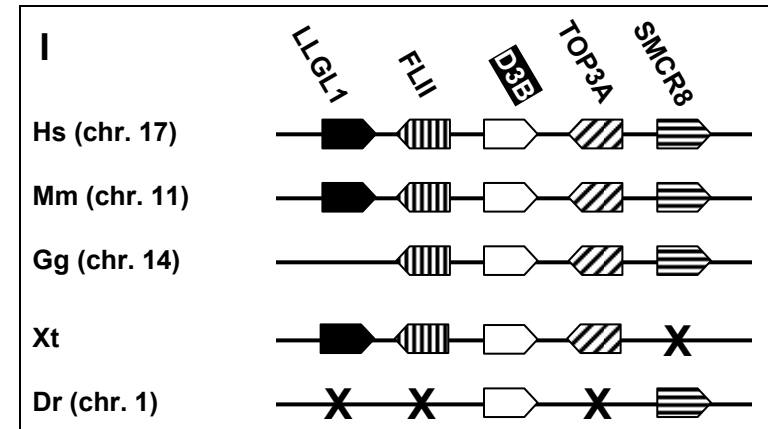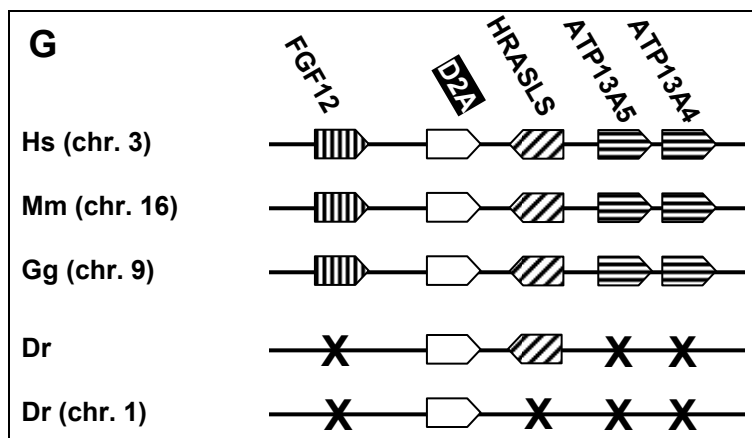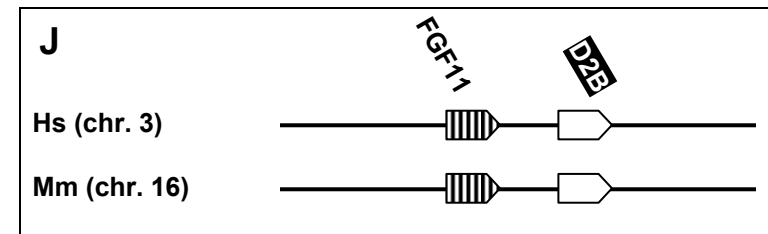

Supplement: Figure S10 — DANGER genes are in conserved synteny among vertebrates (D1–D5) or metazoa (D6). Genes are depicted as pentagon arrows to show transcription orientation. (A) D6 family members are in conserved synteny among all metazoan taxa used in this study, except for D. melanogaster. In particular, the MAB21L1 (L1) genes are located in an intron of the neurobeachin (NBEA) gene and the MAB21L2 (L2) genes are located in an intron of the LPS-responsive vesicle trafficking, beach and anchor containing (LRBA) gene. In data not shown, phylogenetic analysis of the NBEA and LRBA genes from vertebrates, and invertebrates suggests that these genes are homologous and have been duplicated in vertebrates like the L1 and L2 genes. These data suggest that the L1 and L2 genes are products of en block duplication in vertebrates. The coding sequence of D6 family members from N. vectensis, and vertebrates is not interrupted by introns, while the orthologous sequences from ecdysozoa (D. melanogaster and C. elegans), C. intestinalis and S. purpuratus contain introns (see Fig. 3A, 4A, and Figure S2). In the D. melanogaster genome the D6 gene is duplicated and both copies are located on chromosome X, region 5D1-D2, approximately 800 kb downstream of the fly neurobeachin homolog (chromosome X, region 4F3-4F3). This duplication seems to have occurred in all insects, since Anopheles gambiae, Apis mellifera, Tribolium castaneum, and Bombyx mori all contain two copies of the D6 gene (data not shown). (B) D1A genes are in conserved synteny among vertebrates. D. rerio genome contains two copies of the D1A gene. (C) D1B genes are in conserved synteny among mammals. Our exhaustive similarity searches did not reveal D1B orthologs from other vertebrate species; here we show that the X. tropicalis syntenic region bears NUP133 gene in the syntenic position of the mammalian D1B gene. (D) D1C genes are in conserved synteny among warm-blooded animals. (E) D4 family members are in conserved synteny among vertebrates. [file pone.0000204.s010.pdf]

A

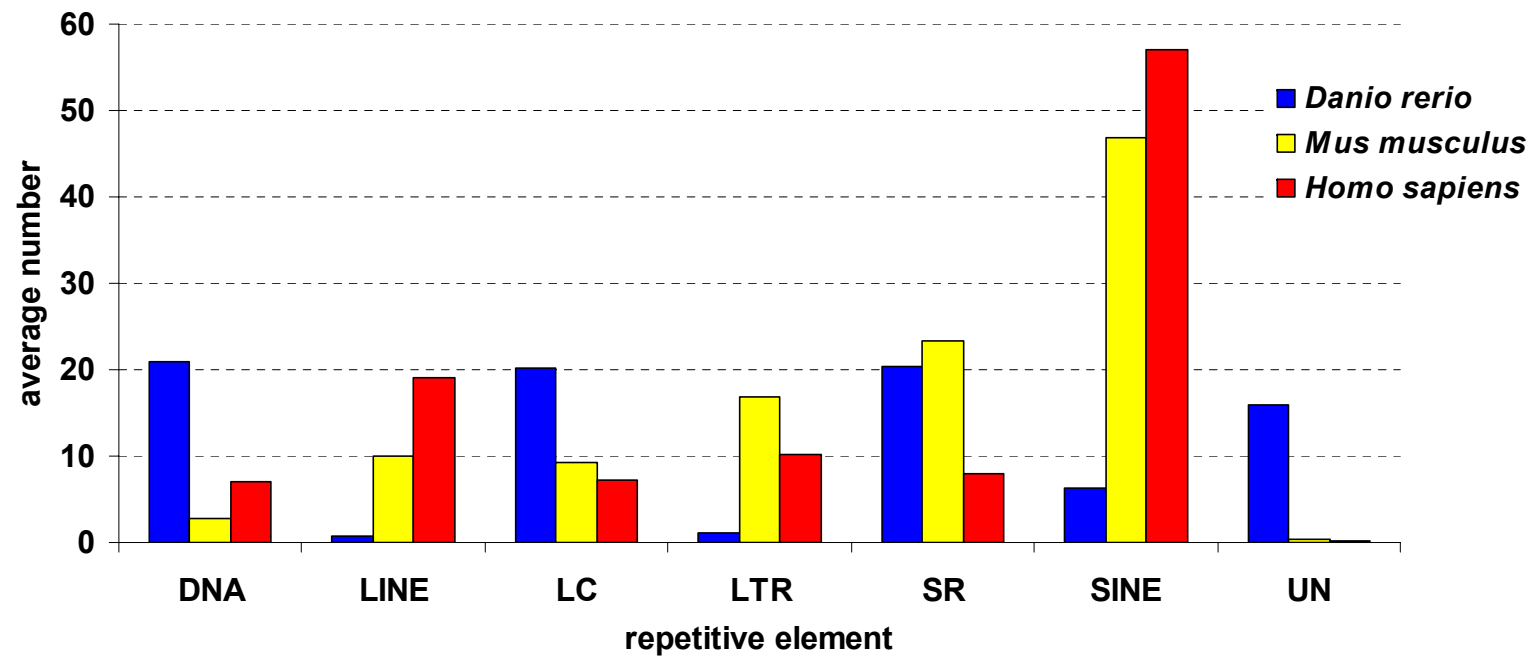

**B**

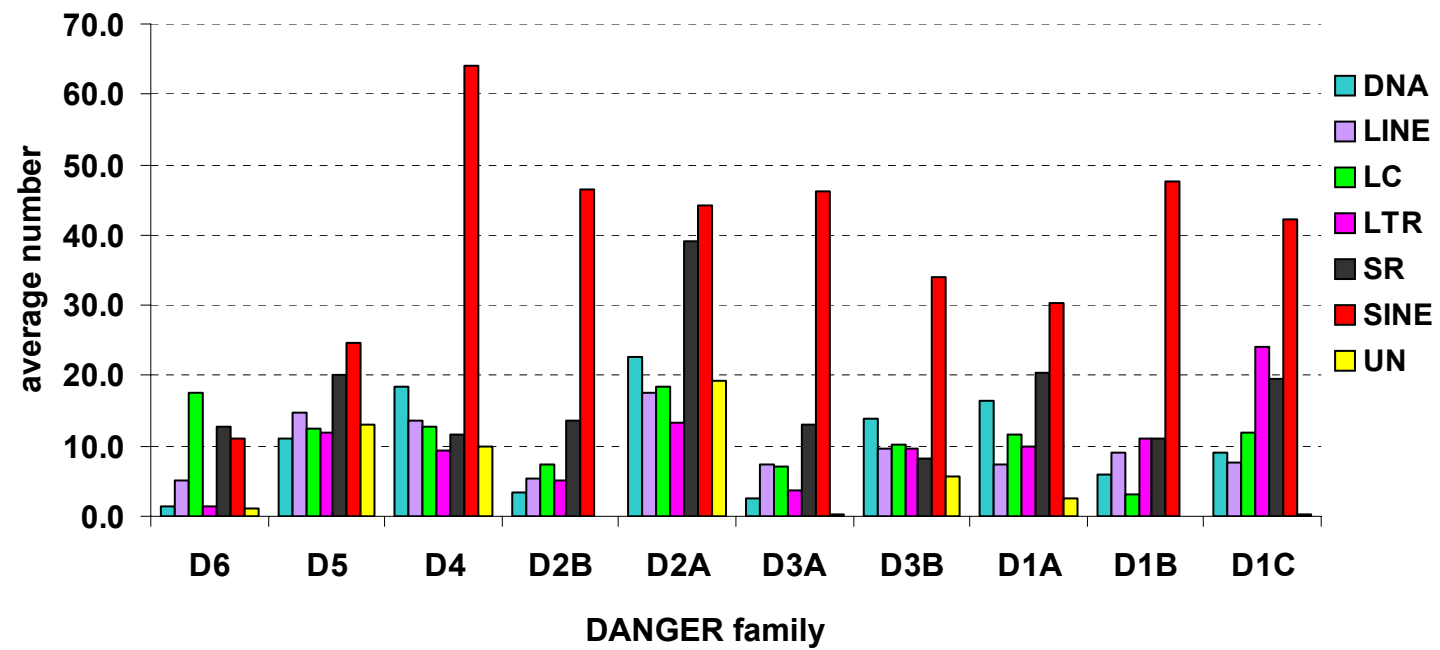

C

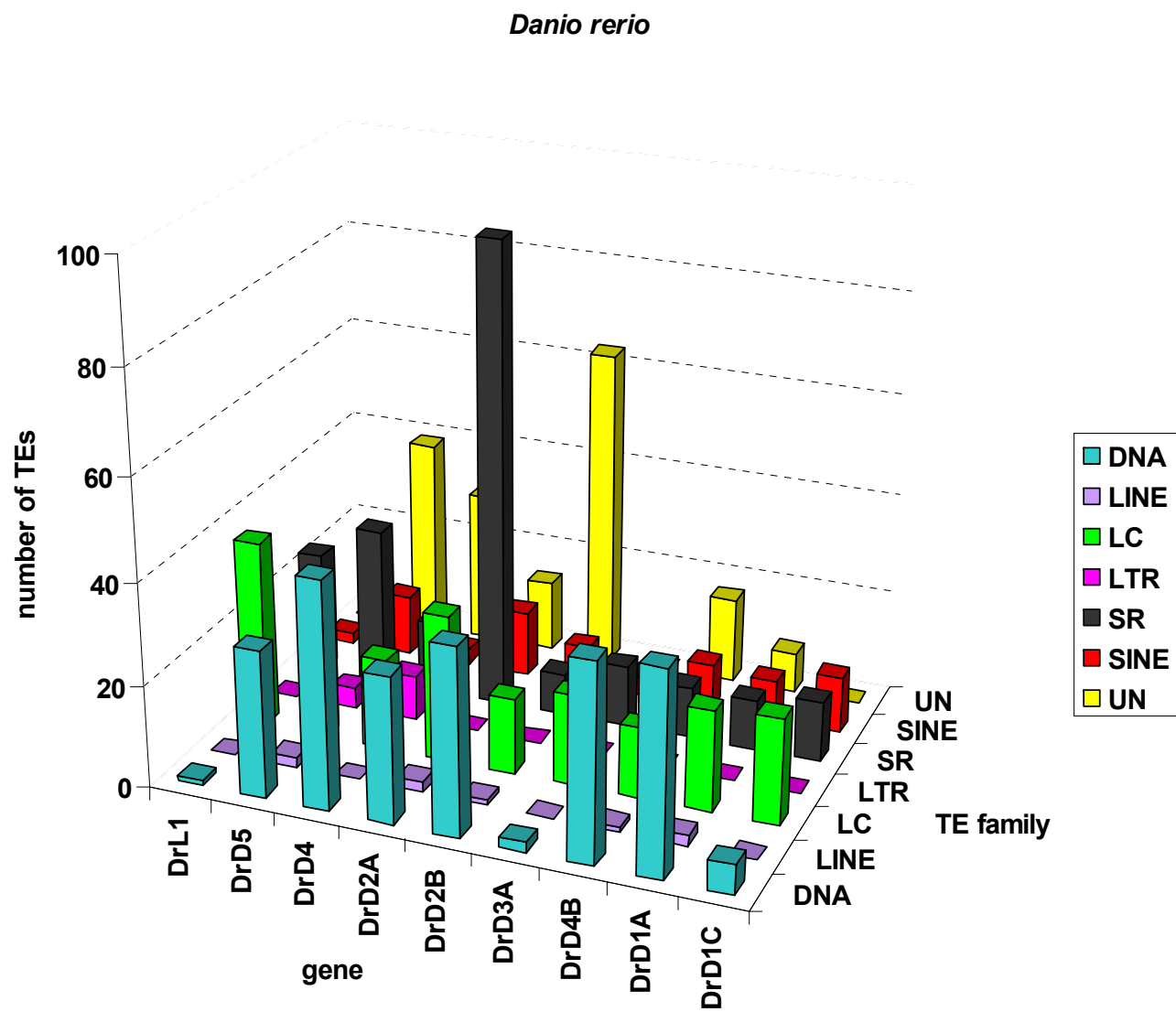

D

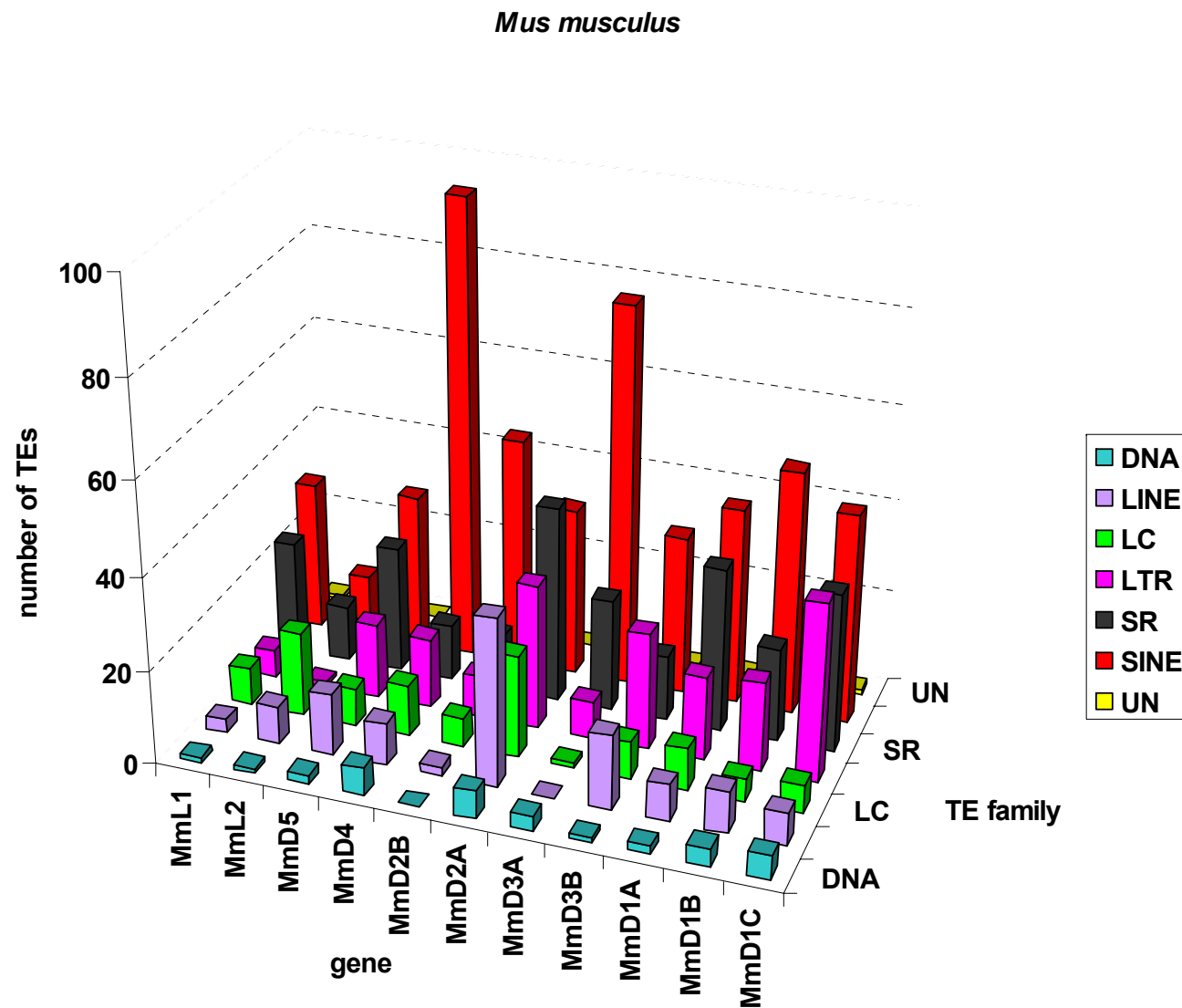

E

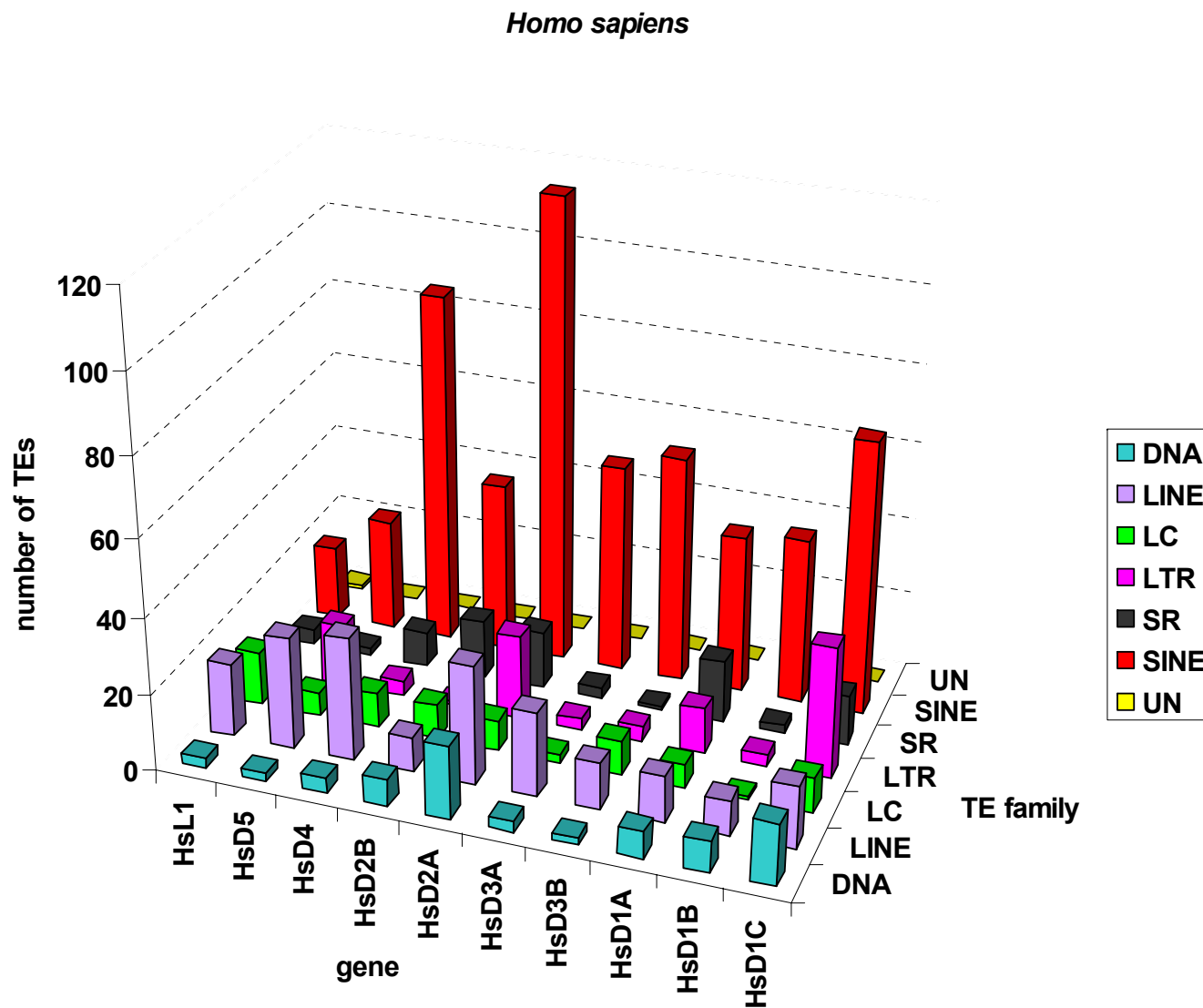

Supplement: Figure S12 — Repeat content of the genomic regions flanking the DANGER genes (approximately 25 Kbp upstream and downstream of each DANGER gene were analyzed). (A) Average number of different repetitive element families in zebrafish, mouse, and human DANGER genomic regions. (B) Average number of different repetitive element families in each DANGER family. (C) Repeat content in the D. rerio DANGER genomic regions. (D) Repeat content in the mouse DANGER genomic regions. (E) Repeat content in the human DANGER genomic regions. DNA, DNA trasnposons; LINE, long-interspersed nucleotide elements; LC, low complexity regions; LTR, retrovirus-like elements with Long Terminal Repeats; SR, simple repeats; SINE, short-interspersed nucleotide elements; UN, unclassified repeats. (0.09 MB PDF) [file pone.0000204.s012.pdf]

**A**

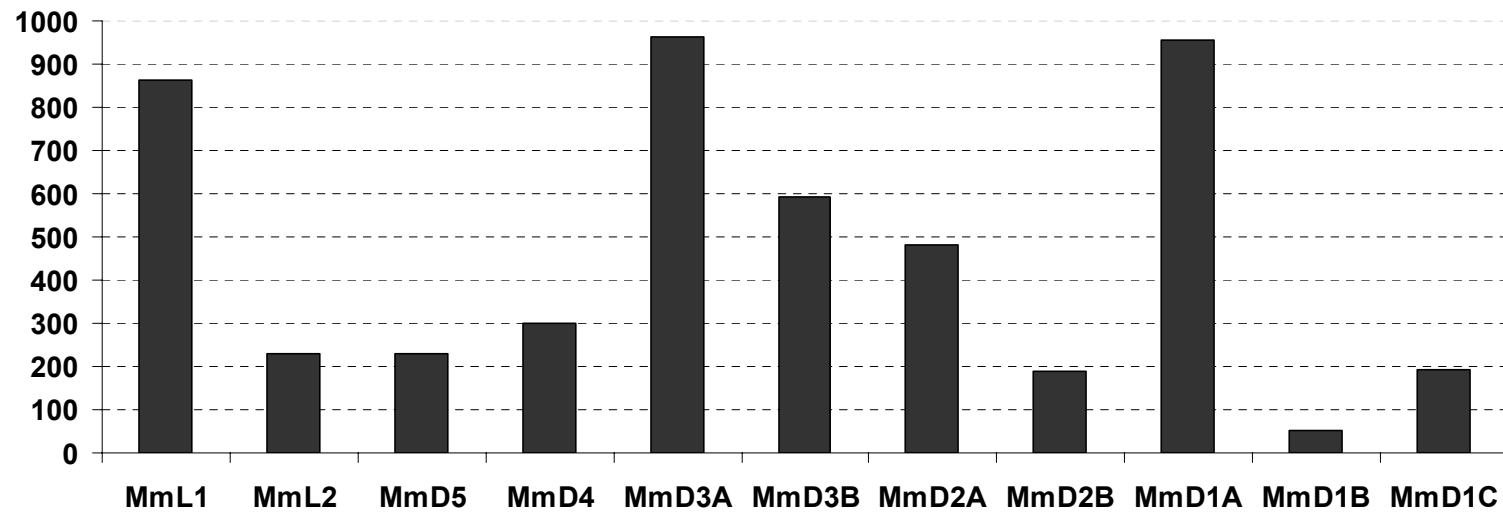

**B**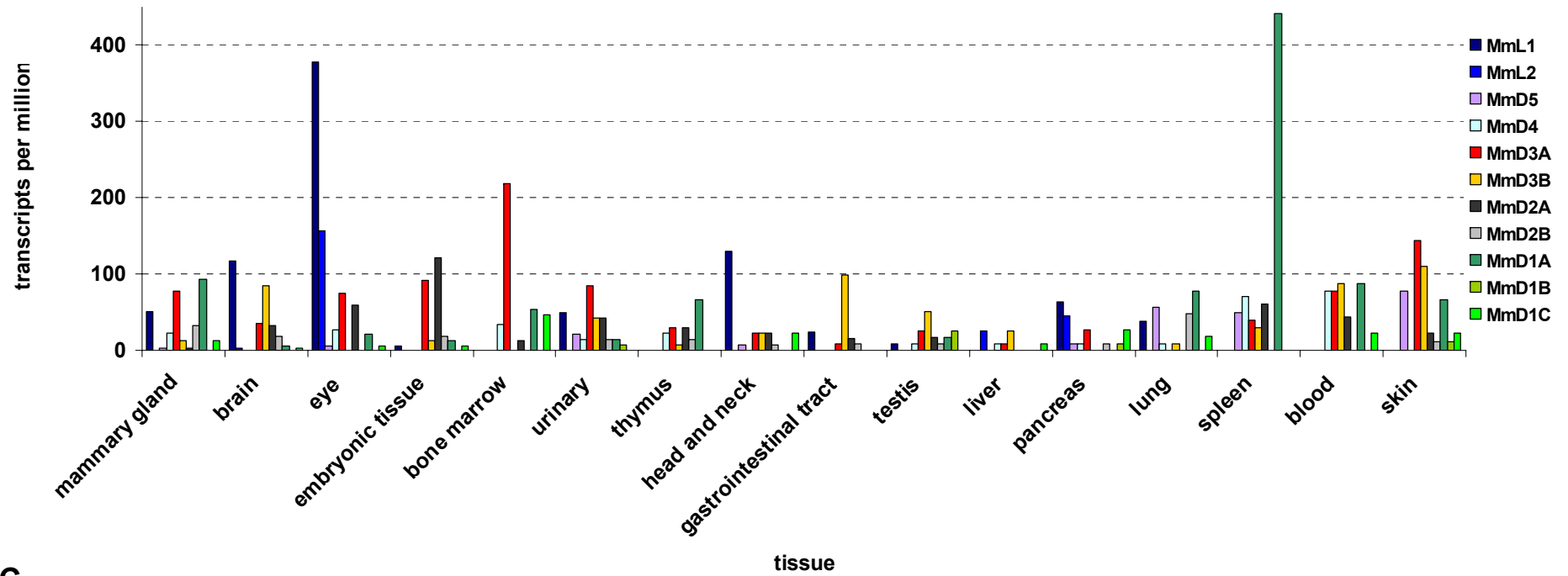**C**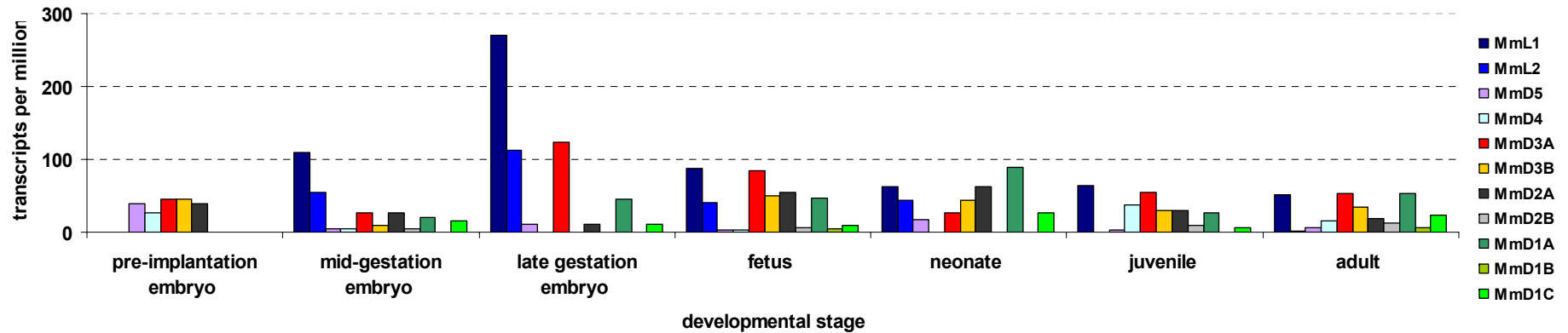

D

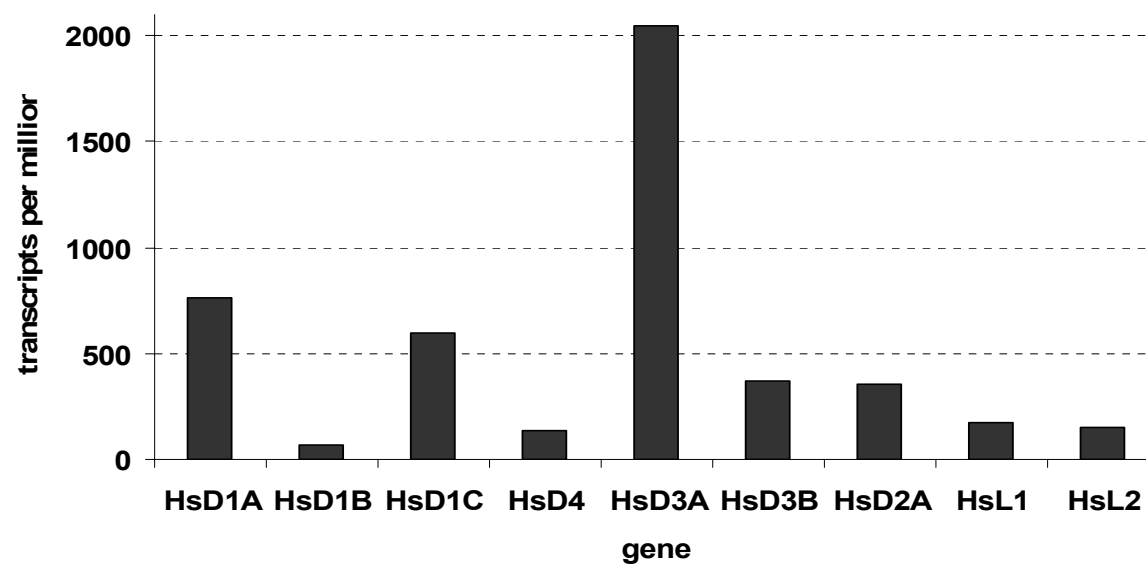

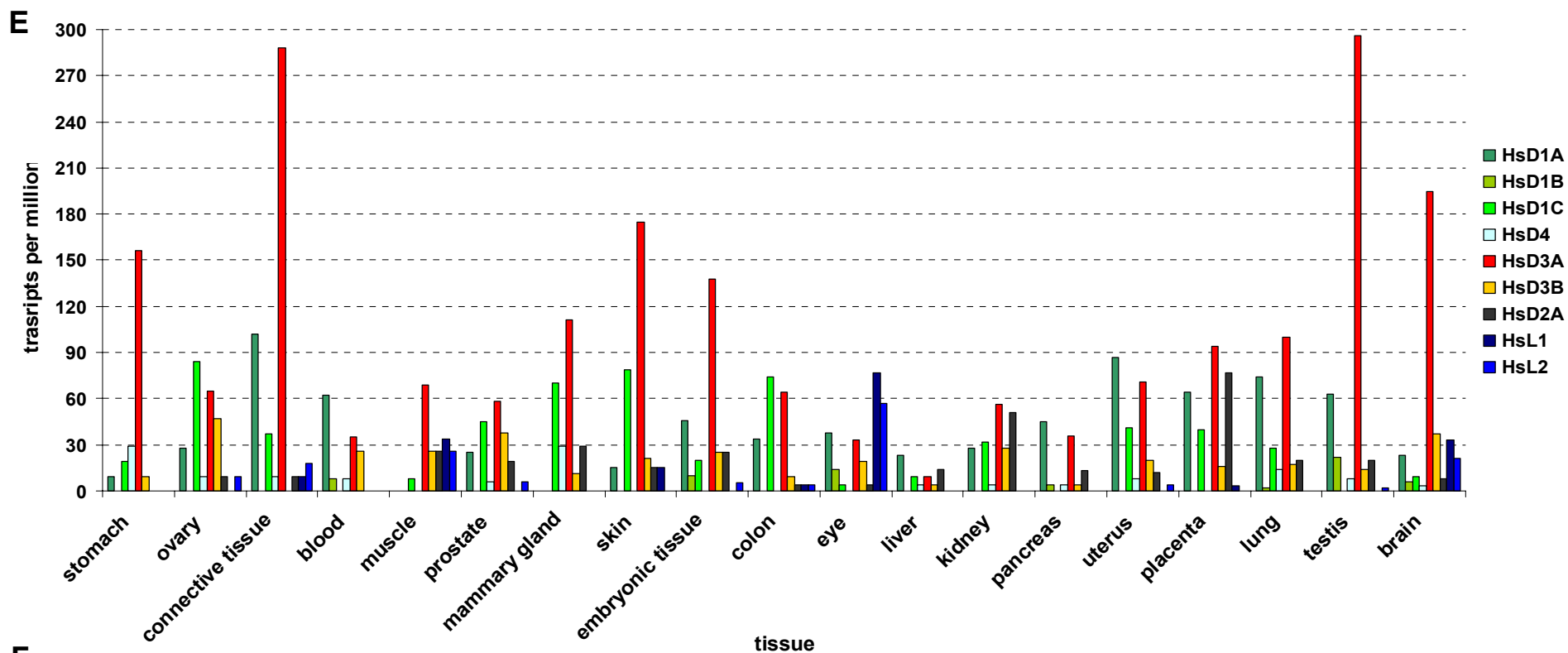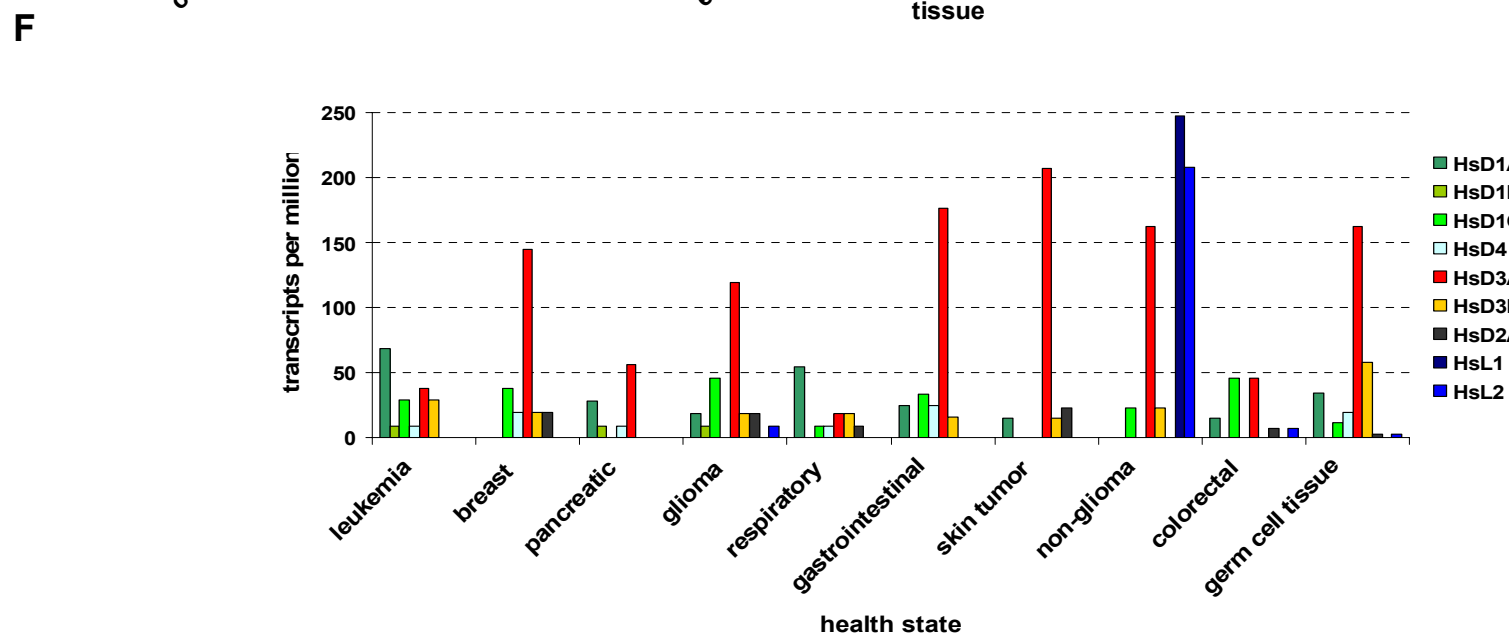

Supplement: Figure S13 — Analysis of expressed sequence tags (ESTs) show that mouse and human DANGER sequences exhibit different transcriptional patterns. (A) Total expression levels (sum of different tissues) for all M. musculus DANGER genes. (B) Expression of M. musculus DANGER genes in different tissues showed as transcripts per million. (C) Expression of M. musculus DANGER genes in different developmental stages. (D) Total expression levels (sum of different tissues) for H. sapiens DANGER genes. (E) Expression of H. sapiens DANGER genes in different tissues. (F) Expression of H. sapiens DANGER genes in different health states. EST data were collected from the UniGene database of NCBI (http://www.ncbi.nlm.nih.gov/entrez/query.fcgi?db = unigene). The UniGene identification number for each sequence is given below. For mouse: MmL1, Mm.384353; MmL2, Mm.389466; MmD5, Mm.32900; MmD4, Mm.101559; MmD3A, Mm.307163; MmD3B, Mm.339760; MmD2A, Mm.155887; MmD2B, Mm.280165; MmD1A, Mm.29457; MmD1B, Mm.57559; MmD1C, Mm.323386; and for human: HsD1A, Hs.523252; HsD1B, Hs.65009; HsD1C, Hs.530899; HsD4, Hs.14577; HsD3A, Hs.148677; HsD3B, Hs.551967; HsD2A, Hs.151443; HsL1, Hs.584776; HsL2, Hs.584852. Tissues and or stages are plotted if they contained more than 80,000 EST sequences. The presence of many ESTs demonstrates that DANGER sequences are expressed and thus DANGERs do not probably represent pseudogenes. The different expression patterns suggest putative functional divergence among paralogous DANGER genes supporting our phylogenetic analyses, which predicts that the different DANGER groups evolve following the model of divergent evolution. (0.07 MB PDF) [file pone.0000204.s013.pdf]

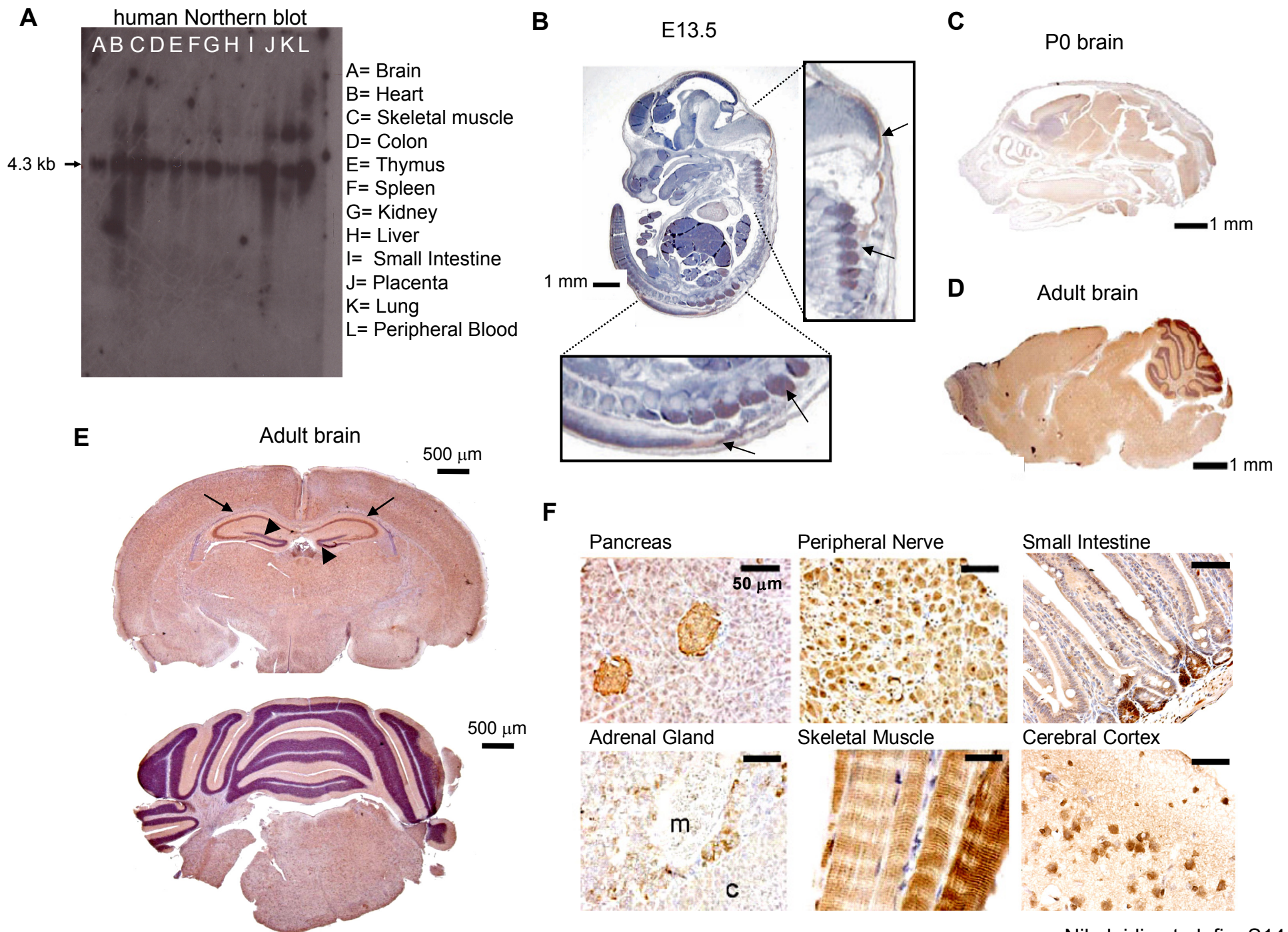

Supplement: Figure S14 — DANGER1A is expressed in the brain and a variety of terminally differentiated tissues. (A) Random primers of HsD1A probed against a multiple tissue Northern Blot. (B–D) Immunohistochemistry on sagittal sections of E13.5 whole mouse, P0 brain and adult brain stained with polyclonal antibody against D1A, respectively. (E) Top: Horizontal brain section depicting the strong D1A staining in the CA1 of the hippocampus (arrow), and the lack of D1A staining in the dente gyrus (arrowhead). Bottom: Horizontal brain section depicting the strong D1A staining in the cerebellum. (F) Clockwise from left: anti-D1A staining in mouse pancreas, peripheral nerve, small intestine, cerebral cortex, skeletal muscle, and adrenal gland. The antibody used is specific to DANGER1a and is described in van Rossum et al. 2006 (Reference number 4 in main text). Antibody reactivity is indicated by the brown staining, while cells absent in DANGER expression are violet in color. (1.22 MB PDF) [file pone.0000204.s014.pdf]

**A**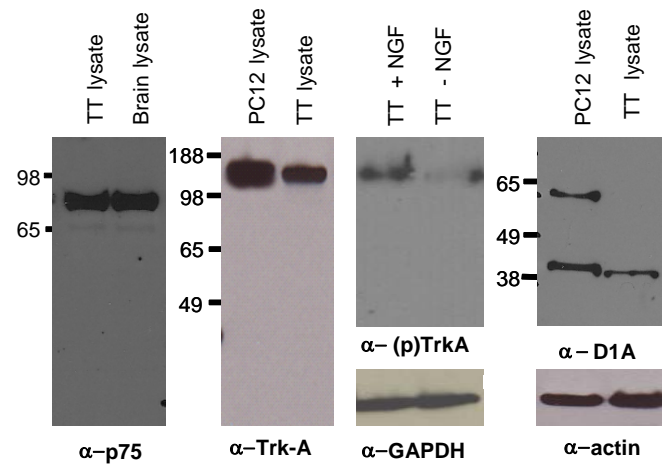**B**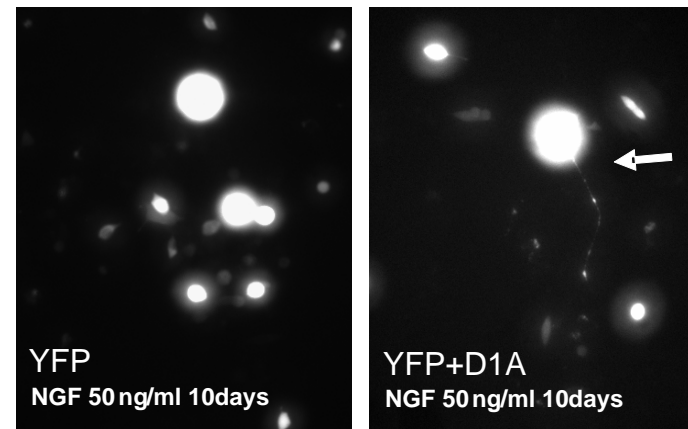

Supplement: Figure S15 — (A) Left: Western analysis of (20 µg) thyroid tumor (TT) cell or Brain lysates blotted with mouse monoclonal antibody against p75. Middle Left: Western analysis of (20 µg) PC12 or TT cell lysates blotted with polyclonal antibody against TrkA. Middle Right: Western analysis of (20 µg) TT cell lysate +/−50 ng/ml NGF for 48 h, blotted with monoclonal antibody against anti phospho-TrkA. GAPDH was used as a loading control. Right: Western analysis of (20 µg) PC12 cell or TT cell lysates blotted with polyclonal antibody against DANGER1A. PC12 cell express a major band (59 kDa) corresponding to the full-length protein and a second band (41 kDa), which is presumed to be a cleavage/breakdown product of D1A protein, since both bands are siRNA-sensitive. TT cells express only the 41 kDa band. Actin was used as a loading control. (B) YFP or YFP+DANGER1A transfected TT cells visualized by epifluorescent microscopy. Arrow denotes neurite growth. (0.18 MB PDF) [file pone.0000204.s015.pdf]
