# Supplementary material for: Ancient Origin of the New Developmental Superfamily DANGER
Source: PLoS One. 2007 Feb 14;2(2):e204. doi: 10.1371/journal.pone.0000204 (PMC1784063; doi:10.1371/journal.pone.0000204)
Supplement: Figure S3 — Multiple sequence alignment of the DANGER superfamily used to generate the phylogenetic tree of Fig. 1A. (0.69 MB PDF) [file pone.0000204.s003.pdf]

```
mouseD2B          ------------------------------------------------------------------------------------------------------------------------------------------------------
ratD2B            ------------------------------------------------------------------------------------------------------------------------------------------------------
humanD2B          ------------------------------------------------------------------------------------------------------------------------------------------------------
mouseD2A          ------------------------------------------------------------------------------------------------------------------------------------------------------
ratD2A            MASSICFACVLSAVKVGVDLYWLQSPLSAEDIWEGKHLLFGVIKDFPSAQRRIAVFLNSFRACQLKNLEPRRLAVVGDEEDQIPTPSWSCFLQPPAHSLCVLMNIERLEKQRGKGDSRLICILSNWARETRHQGCGDTNELQSTFKESPL
humanD2A          ------------------------------------------------------------------------------------------------------------------------------------------------------
chickenD2A        ------------------------------------------------------------------------------------------------------------------------------------------------------
frogD2A           ------------------------------------------------------------------------------------------------------------------------------------------------------
zebrafishD2A1     ------------------------------------------------------------------------------------------------------------------------------------------------------
zebrafishD2A2     ------------------------------------------------------------------------------------------------------------------------------------------------------
cionaD2A          ------------------------------------------------------------------------------------------------------------------------------------------------------
mouseD3A          --------------------------------------------------------------------------------------------------------------------------------MAGAGERKGKKDDNGIGTAIDF
ratD3A            --------------------------------------------------------------------------------------------------------------------------------MAGAGERKGKKDDNGIGTAIDF
humanD3A          --------------------------------------------------------------------------------------------------------------------------------MAGAGERKGKKDDNGIGTAIDF
chickenD3A        ------------------------------------MATWSRQAVLTLYRALLREGRGLRYTDRDFYLASIRREFRKNRGLQRLEDKERQLEKGQAFLQSKLGGLVADSWMSLKYLVPAVVKYESESEMAGAGPRKGKKDDNGIGTAIDF
frogD3A           --------------------------------------------------------------------------------------------------------------------------------MAGAGEKKGKKDDNGIGTAIDF
zebrafishD3A      -------------------------------------------------------------------------------------------------------------------------------MAGVNGDRKGKKDDNGLGTAIDF
mouseD3B          -------------------------------------------------------------------------------------------------------------------------------MAEFSQKQRKQSGSEGLGSVVDF
ratD3B            ------------------------------------------------------------MALVSCALRLERRPAYPAFLSGHLPPRASAFGSRGLDAPFLSGSSWASRVLLAASLGPCADARTQVTMAEFSQKQRKQHGGEGLGSVVDF
humanD3B          -------------------------------------------------------------------------------------------------------------------------------MAEFSQKRGKRRSDEGLGSMVDF
chickenD3B        -------------------------------------------------------------------------------------------------------------------------------MAEFTQQSCKRRDDGGLGSVLDL
mouseD1A          -------------------------------------------------------------------------------------------------------------------MAMELFRVCLVVVTAIINHPLLF-PRENATIPENE
ratD1A            -------------------------------------------------------------------------------------------------------------------MAMGLFRVCLVVVTAIINHPLLF-PRENATVPENE
humanD1A          -------------------------------------------------------------------------------------------------------------------MAMGLFRVCLVVVTAIINHPLLFP-RENATVPENE
chickenD1A        -------------------------MNSAELAGARLENCWAGSICFGAGLESSRSLQDRSTKERISPGLPALPRQTDKAFCNAGCTQPALVVKIAVTQLAVWVPKRDSCGSEGTAMPMGIFRVCLVVITAIVNHPLLFP-KENGTVLENT
frogD1A           -------------------------------------------------------------------------------------------------------------------MQFGIFKVCLVVVTAIVNHPLLFP-NENATLP--E
zebrafishD1A1     -------------------------------------------------------------------------------------------------------------------MQGAIARVCMVVVAAILNHPLLF-PNENTTVPEQD
zebrafishD1A2     -------------------------------------------------------------------------------------------------------------------MQGAIARVCMVVVAAILNHPLLF-PNENTTVPEQD
mouseD1B          -----------------------------------------------------------------------------MGKTLPEQDSVFSACGREPLGLAGHIWGPRWTDESDAEASMAVISLMFLAVMYVVHHPLMVSDRMDLDTLARS
ratD1B            ---------------------------------------------------------------------------------------------------------------------MAVISLLFLAVMYVVHHPLMVSDRMDLDTLARS
humanD1B          -----------------------------------------------------------------------------------------------------MTTDSYSPTSPDAEASMAVISLLFLAVMYVVHHPLMVSDRMDLDTLARS
mouseD1C          --------------------------------------------------------------------------------------------------------------------------------------------------MSVR
ratD1C            --------------------------------------------------------------------------------------------------------------------------------------------------MSVR
humanD1C          --------------------------------------------------------------------------------------------------------------------------------------------------MSVH
frogD1C           --------------------------------------------------------------------------------------------------------------------------------------------------MPV-
zebrafishD1C      ----------------------------------------------------------------------------MRGLLLPSAHAASVKEPNRVNFSLHDNFCSKWWKEHQLGLIFSIWTSGSAWASPDHSYRAEVDWRRSRR-RMSV
mouseD4           ------------------------------------------------------------------MEDPRRRTTAPRAKKPSAKRAPTQPSRTRAHAESCGPQRGAR------SRRAERDGDTT-------------------------
humanD4           ---------------------------------------------------------MQPWHGKAMQRASEAGATAPKASARNARGAPMDPTESPAAPEAALPKAGKFGPARKSGSRQKKSAPDTQ------------------------
chickenD4         ----------------------------------------------------------------------------------MAAAGGRNGKRSRAGG----------------------------------------------------
zebrafishD4       ----------------------------------------------------------MSSHRRPGCVSPTRPDEQPKAKARAKTSGKNEAQKKDKDVYVCESEAKLQETPSLKRGRSKKQKDDASKGDCKSLDNSESKSEEKHGDISKA
DmD4              ------------------------------------------------------------------------------------------------------------------------------------------------------
mouseD5           ------------------------------------------------------------------------------------------------------------------------------------------------------
ratD5             ------------------------------------------------------------------------------------------------------------------------------------------------------
humanD5           ------------------------------------------------------------------------------------------------------------------------------------------------------
chickenD5         ------------------------------------------------------------------------------------------------------------------------------------------------------
zebrafishD5       ------------------------------------------------------------------------------------------------------------------------------------------------------
mouseMAB21L2      ------------------------------------------------------------------------------------------------------------------------------------------------------
ratMAB21L2        ------------------------------------------------------------------------------------------------------------------------------------------------------
humanMAB21L2      ------------------------------------------------------------------------------------------------------------------------------------------------------
frogMAB21L2       ------------------------------------------------------------------------------------------------------------------------------------------------------
zebrafishMAB21L2  ------------------------------------------------------------------------------------------------------------------------------------------------------
mouseMAB21L1      ------------------------------------------------------------------------------------------------------------------------------------------------------
ratMAB21L1        ------------------------------------------------------------------------------------------------------------------------------------------------------
humanMAB21L1      ------------------------------------------------------------------------------------------------------------------------------------------------------
frogMAB21L1       ------------------------------------------------------------------------------------------------------------------------------------------------------
zebrafishMAB21L1  ------------------------------------------------------------------------------------------------------------------------------------------------------
urchin            ------------------------------------------------------------------------------------------------------------------------------------------------------
ciona             ------------------------------------------------------------------------------------------------------------------------------------------------------
fly1              ------------------------------------------------------------------------------------------------------------------------------------------------------
fly2              ------------------------------------------------------------------------------------------------------------------------------------------------------
C.elegans         ------------------------------------------------------------------------------------------------------------------------------------------------------
C.briggsae        ------------------------------------------------------------------------------------------------------------------------------------------------------
Nv67_67_1         ------------------------------------------------------------------------------------------------------------------------------------------------------
Nv094             ------------------------------------------------------------------------------------------------------------------------------------------------------
Nv095             ----------------------------------------------------------------------MQNQGFESSESEFLRRQDDISRKDKDRLYKTNFFSSRRGNAQGKENRGLVLSPGHSTSSLSIESERDHKDRKTRVDDESG
Nv097             -----------------------------------------------------------------MASPQDNTVLTNALRAFFEREVKITSGDKGMTKEMIDNHIKGHILEYFKDNSHISVRKLDYTGSFYEKLKTGAADEVDIMVILES
Nv067             ------------------------------------------------------------------------------------------------------------------------------------------------------
Mo                ------------------------------------------------------------------------------------------------------------------------------------------------------
ruler             1.......10........20........30........40........50........60........70........80........90.......100.......110.......120.......130.......140.......150
```

```
mouseD2B ----------------------------------------------------------------MASTVWGGAPWWGPPPPAPARPLTDIDFCSGAQLQELTQLIQ--------------------------------------------
ratD2B -----------------------------------------------------------------LSFSWGSTGWGDPPPPPAARPLTDIDFCSGAQLQELTQLIQ--------------------------------------------
humanD2B ----------------------------------------------------------------MASAVWGSAPWWGPPPPAPARPLTDIDFCSGAQLQELTQLIQ--------------------------------------------
mouseD2A -----------------------------------------------------------------MKMAAPTASKAASLGCNNKPAFPELDFRSGARVEELNKLIQ--------------------------------------------
ratD2A LIKSWRLKTITQHRLWILNVLQRGLNSPFTLSGVMRPSGQLCSFDHPPPLMGLQRPHFWALFGNHTAMDSELSHQPYTYQFMYEHSDLTLKNTRGPGTEVPMLLKFMVLCKSHCDSCKLAPCRVLCREGE--------------------
humanD2A -------------------------------------------------------------------MAAPTANKAASLGCNNKPAFPELDFRSGARVEELNKLIQ--------------------------------------------
chickenD2A -------------------------------------------------------------------MAAPLASKAGSAGTNSKPPFPELDFRSGARVEELNKLIQ--------------------------------------------
frogD2A -------------------------------------------------------------------MAAPIPTKPASVGGGSKPSSPELDFRSGARIEELNKLIQ--------------------------------------------
zebrafishD2A1 ------------------------------------------------MAAPAPSSRTGSVSSSLGNSPTATPGPGGLNNGHKLWCCHELDFRSGVKIEELNRLIH--------------------------------------------
zebrafishD2A2 ------MPGPVVPGDQVYLKVFRRKWNEPRREGPYRVVRATPTSIQVEGSTTWYHLNHCTRVPKEKVTAGDHQRETASPEKPTAESDETYTRRWNLRIHGIPEKDK--------------------------------------------
cionaD2A --------------------------------------------------------------------------------METKHGHQKLDFRSGCSLNRLENLIN--------------------------------------------
mouseD3A VLSNAR----------------------------------------------------------------LVLGVGGAAMLGIATLAVKRMYDRAISAPTS-------------------------------------------------
ratD3A VLSNAR----------------------------------------------------------------LVLGVGGAAMLGIATLAVKRMYDRAISAPTS-------------------------------------------------
humanD3A VLSNAR----------------------------------------------------------------LVLGVGGAAMLGIATLAVKRMYDRAISAPTS-------------------------------------------------
chickenD3A VLSNAR----------------------------------------------------------------LVLGVGGAAMLGIATLAVKRMYDRAISAPS--------------------------------------------------
frogD3A VLSNAR----------------------------------------------------------------LVLGVGGAAMLGIATLAVKRMYDRAISAPAS-------------------------------------------------
zebrafishD3A VLSNAK----------------------------------------------------------------LVLGVGGAAMLGIATLAVKRMYDRALSAPS--------------------------------------------------
mouseD3B LLANAR----------------------------------------------------------------LVLGVGGAAVLGIATLAVKRLIDRATSPPD--------------------------------------------------
ratD3B LLANAR----------------------------------------------------------------LVLGVGGAAVLGIATLAVKRLIDRATNPRD--------------------------------------------------
humanD3B LLANAR----------------------------------------------------------------LVLGVGGAAVLGIATLAVKRFIDRATSPRD--------------------------------------------------
chickenD3B LLANAR----------------------------------------------------------------LVLGVSGAAVLAIATLAVKRLIDRATSPREE-------------------------------------------------
mouseD1A EEIIR-------------------------------------------------------------------KMQEHQEKLRLEQLRLEEEVSRLEAEKEALRQVEEE-----QQQLEAHTAWDL-------------------------
ratD1A EEIIR-------------------------------------------------------------------KMQEHQEKLQMEQLRLEEEVSRLVAEKEALRQIEDEDQ---QQQLEAHTTWD--------------------------
humanD1A EEIIR-------------------------------------------------------------------KMQAHQEKLQLEQLRLEEEVARLAAEKEALEQVAEEG----RQQNETRVAWD--------------------------
chickenD1A EEIIQ-------------------------------------------------------------------KMKEREEILRLEKLRLEQEIEGQEAAQKVLEKAAKVV----EDVKEEKVQWD--------------------------
frogD1A EQIIQ-------------------------------------------------------------------KMKDREESLKAEQMHLEQMFSSAVTKEKREEEEASQSTEASEDEP-----WD--------------------------
zebrafishD1A1 EDLLA-------------------------------------------------------------------RMKEHQEKLEAEQKRLEQEISQN--ETSVIGDQ------------DGYG-WY--------------------------
zebrafishD1A2 EDLLA-------------------------------------------------------------------RMKEHQEKLEAEQKRLEQEISQN--ETSVIGDQ------------DGYG-WY--------------------------
mouseD1B RQLEK----------------------------------------------------------------------RMSEEMRQLEMEFEERSRAAEQKQKVENFWRGDTSSDQLVLGKKDMGWPFQAGGQDGG---------PLGWILGN
ratD1B RQLEK----------------------------------------------------------------------RMSEEMRQLEIEFEERSRAAEEKQKAENFWRGDTSNDQLVLGKKDMRWPFQASGQDGG---------PLGWMLGN
humanD1B RQLEK----------------------------------------------------------------------RMSEEMRLLEMEFEERKRAAEQRQKAENFWTGDTSSDQLVLGKKDMGWPFQADGQEG----------PLGWMLGN
mouseD1C YTLNL------------------------------------------------------------------RVFWPLVTGLCTALVCLYHALR-SSEDARAESPDGADS-----------------------------------------
ratD1C YTLNL------------------------------------------------------------------RVFWPLVTGLCTALVCLYHALR-SSEGARAEPPDGADS-----------------------------------------
humanD1C YTLNL------------------------------------------------------------------RVFWPLVTGLCTALVCLYHVLR-GSGGARAEPADGVDG-----------------------------------------
frogD1C YSLNL------------------------------------------------------------------KVFWLLIAFLFTGIWCLFVAWRRDGAAQEEGSCPEN---------------------------------------GFNH
zebrafishD1C YTLNL------------------------------------------------------------------RFFWPLVTCILTALLLLHQYIFRGLEDQGSDCADQG-----------------------------------------PG
mouseD4 ----------EKPRAPG------------------------------------------------------------------PRVHPARATELTKDAQPS-AMDAAG------------------------ATARPAVR----------
humanD4 ----------ERPPVRA------------------------------------------------------------------TGARAKKAPQRAQDTQPSDATSAPGAEG----------------------LEPPAAR----------
chickenD4 ----------GVPPERR------------------------------------------------------------------CRVAAER--------------------------------------------PLAAAR----------
zebrafishD4 GSVHKKTGENDRQDSAK------------------------------------------------------------------DKLKSKRKTAPLDCESEGKNQQTANGKS-----------------GEKTALKRPSKKEK--------
DmD4 ------------------------------------------------------------------------------------------------------------------------------------------------------
mouseD5 ------------------------------------------------------------------------------------------------------------------------------------------------------
ratD5 ------------------------------------------------------------------------------------------------------------------------------------------------------
humanD5 ------------------------------------------------------------------------------------------------------------------------------------------------------
chickenD5 ------------------------------------------------------------------------------------------------------------------------------------------------------
zebrafishD5 ------------------------------------------------------------------------------------------------------------------------------------------------------
mouseMAB21L2 ------------------------------------------------------------------------------------------------------------------------------------------------------
ratMAB21L2 ------------------------------------------------------------------------------------------------------------------------------------------------------
humanMAB21L2 ------------------------------------------------------------------------------------------------------------------------------------------------------
frogMAB21L2 ------------------------------------------------------------------------------------------------------------------------------------------------------
zebrafishMAB21L2 ------------------------------------------------------------------------------------------------------------------------------------------------------
mouseMAB21L1 ------------------------------------------------------------------------------------------------------------------------------------------------------
ratMAB21L1 ------------------------------------------------------------------------------------------------------------------------------------------------------
humanMAB21L1 ------------------------------------------------------------------------------------------------------------------------------------------------------
frogMAB21L1 ------------------------------------------------------------------------------------------------------------------------------------------------------
zebrafishMAB21L1 ------------------------------------------------------------------------------------------------------------------------------------------------------
urchin ------------------------------------------------------------------------------------------------------------------------------------------------------
ciona ------------------------------------------------------------------------------------------------------------------------------------------------------
fly1 ------------------------------------------------------------------------------------------------------------------------------------------------------
fly2 ------------------------------------------------------------------------------------------------------------------------------------------------------
C.elegans ------------------------------------------------------------------------------------------------------------------------------------------------------
C.briggsae ------------------------------------------------------------------------------------------------------------------------------------------------------
Nv67_67_1 ------------------------------------------------------------------------------------------------------------------------------------------------------
Nv094 ------------------------------------------------------------------------------------------------------------------------------------------------------
Nv095 WVFVPN---------------------------------------------------------------IGVRHSRLELLTALNNYSDNVVCLRRLYFQQAMNQIEVMVN----------------------------------------
Nv097 KDLKLEKVNDFPGWVKLKATD-----------------------------------------------GSPFEKYANDDRYIIPEKILKSWMFSSVQKAVNKFSKEKPDLFGTLKISLHGPAVQLDINAGLSVDLVLTFEFDDEHYVAKP
Nv067 ------------------------------------------------------------------------------MEIDLATGLYWKDISSLLNEAFSDFKKDFS------------------------------------------
Mo ------------------------------------------------------------------------------------------------------------------------------------------------------
ruler .......160.......170.......180.......190.......200.......210.......220.......230.......240.......250.......260.......270.......280.......290.......300
```

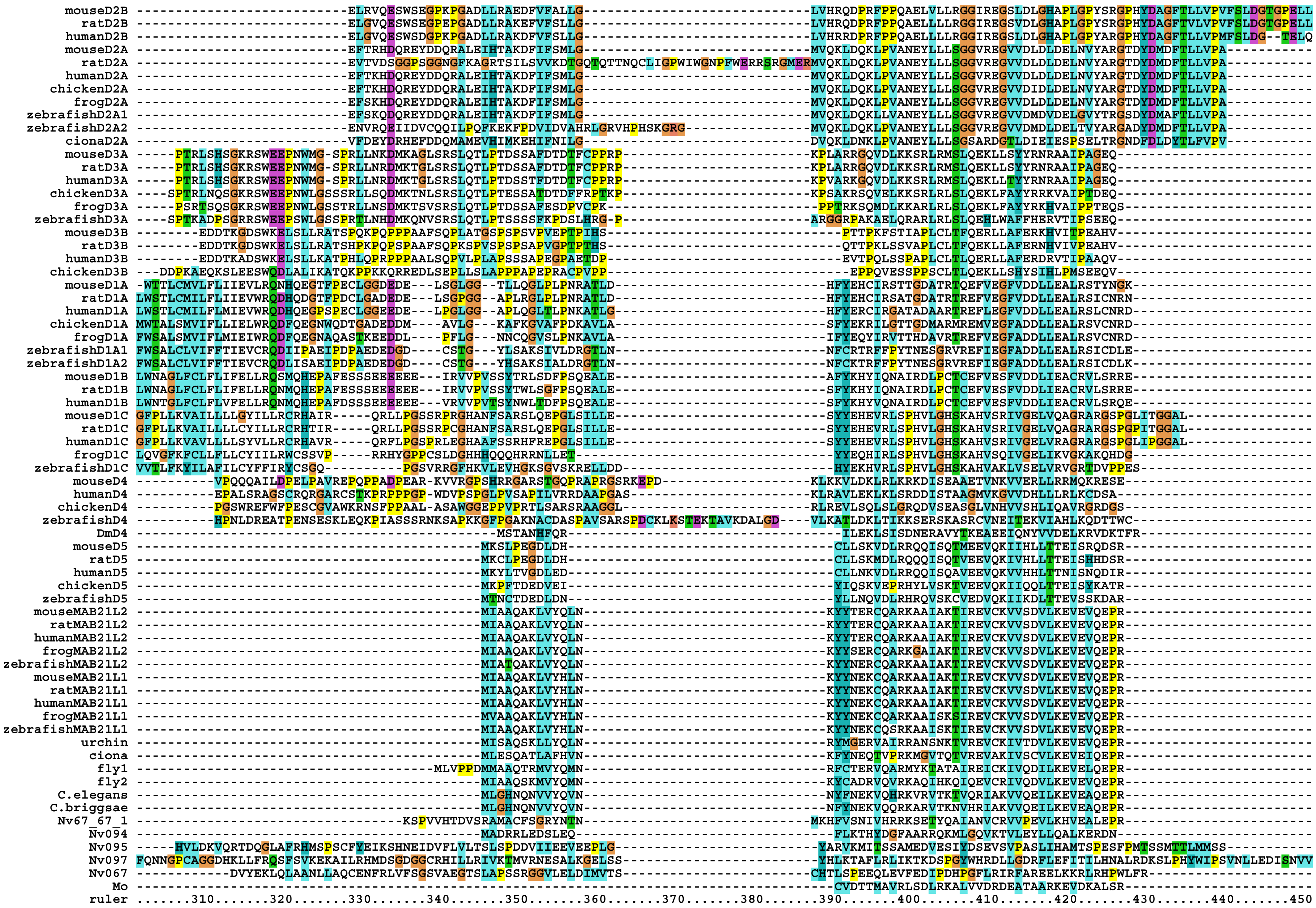

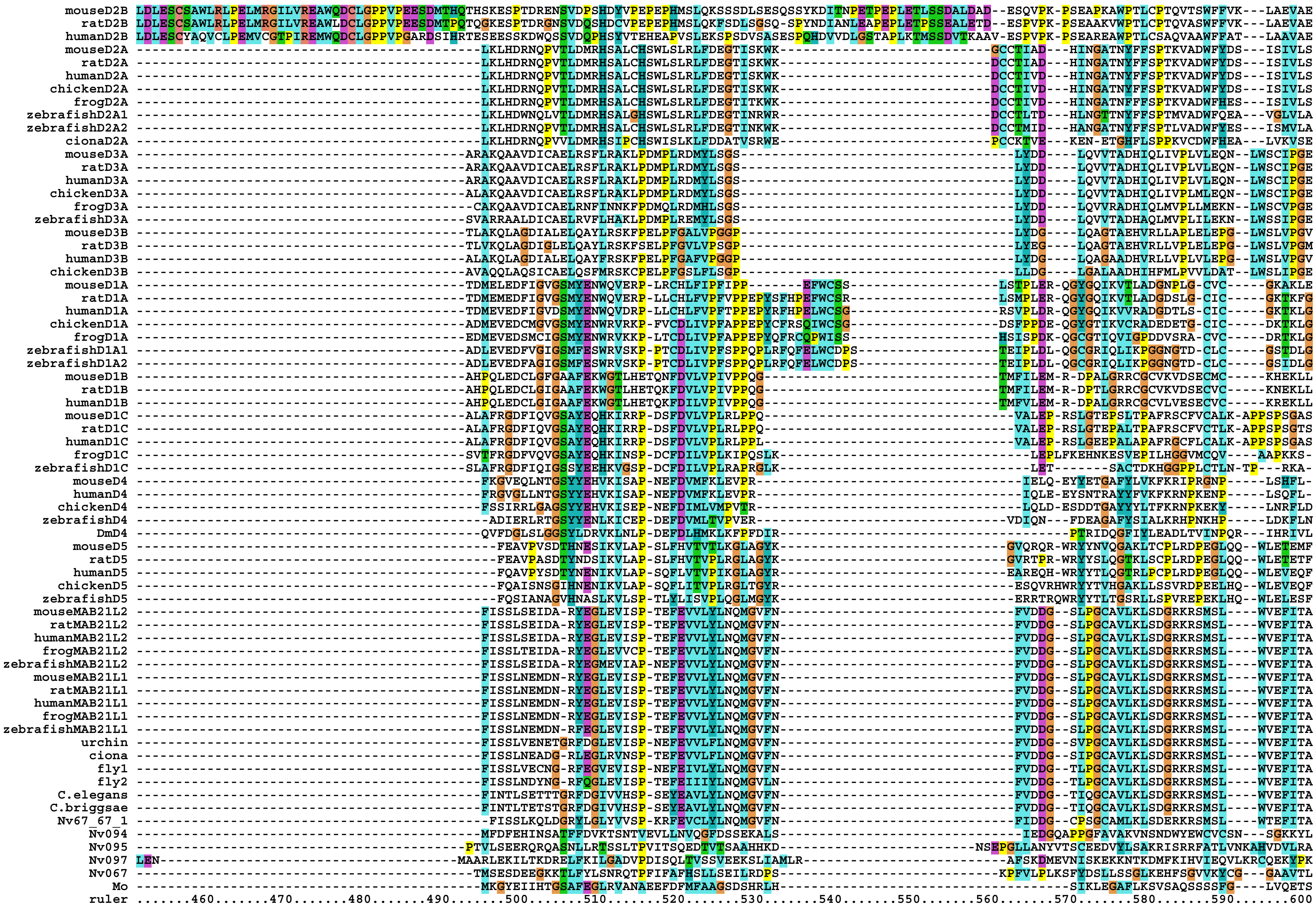
mouseD2B
ratD2B
humanD2B
mouseD2A
ratD2A
humanD2A
chickenD2A
frogD2A
zebrafishD2A1
zebrafishD2A2
cionaD2A
mouseD3A
ratD3A
humanD3A
chickenD3A
frogD3A
zebrafishD3A
mouseD3B
ratD3B
humanD3B
chickenD3B
mouseD1A
ratD1A
humanD1A
chickenD1A
frogD1A
zebrafishD1A1
zebrafishD1A2
mouseD1B
ratD1B
humanD1B
mouseD1C
ratD1C
humanD1C
frogD1C
zebrafishD1C
mouseD4
humanD4
chickenD4
zebrafishD4
DmD4
mouseD5
ratD5
humanD5
chickenD5
zebrafishD5
mouseMAB21L2
ratMAB21L2
humanMAB21L2
frogMAB21L2
zebrafishMAB21L2
mouseMAB21L1
ratMAB21L1
humanMAB21L1
frogMAB21L1
zebrafishMAB21L1
urchin
ciona
fly1
fly2
C.elegans
C.briggsae
Nv67_67_1
Nv094
Nv095
Nv097
Nv067
Mo
ruler .......460.......470.......480.......490.......500.......510.......520.......530.......540.......550.......560.......570.......580.......590.......600

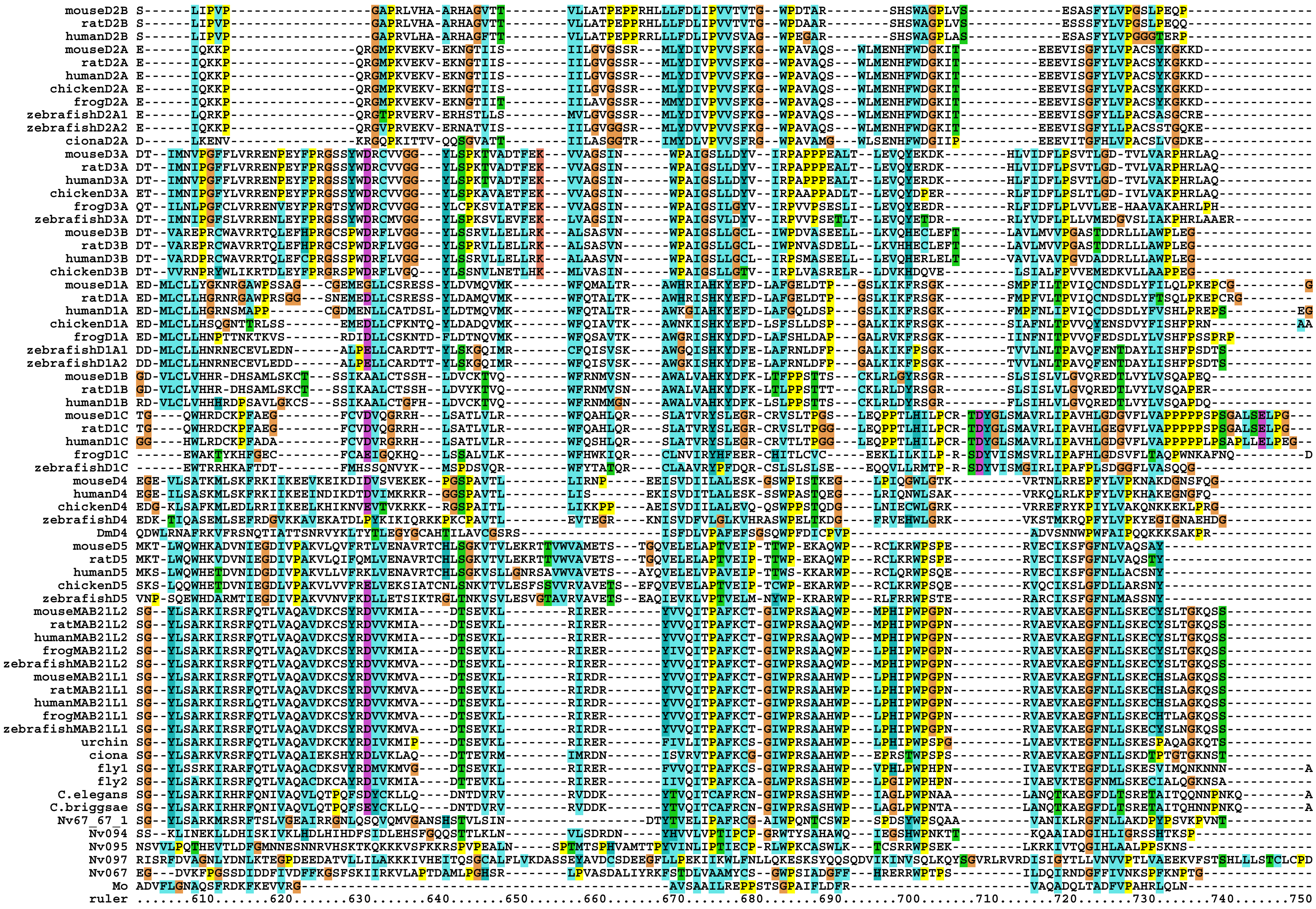
mouseD2B
ratD2B
humanD2B
mouseD2A
ratD2A
humanD2A
chickenD2A
frogD2A
zebrafishD2A1
zebrafishD2A2
cionaD2A
mouseD3A
ratD3A
humanD3A
chickenD3A
frogD3A
zebrafishD3A
mouseD3B
ratD3B
humanD3B
chickenD3B
mouseD1A
ratD1A
humanD1A
chickenD1A
frogD1A
zebrafishD1A1
zebrafishD1A2
mouseD1B
ratD1B
humanD1B
mouseD1C
ratD1C
humanD1C
frogD1C
zebrafishD1C
mouseD4
humanD4
chickenD4
zebrafishD4
DmD4
mouseD5
ratD5
humanD5
chickenD5
zebrafishD5
mouseMAB21L2
ratMAB21L2
humanMAB21L2
frogMAB21L2
zebrafishMAB21L2
mouseMAB21L1
ratMAB21L1
humanMAB21L1
frogMAB21L1
zebrafishMAB21L1
urchin
ciona
fly1
fly2
C.elegans
C.briggsae
Nv67_67_1
Nv094
Nv095
Nv097
Nv067
Mo
ruler .......610.......620.......630.......640.......650.......660.......670.......680.......690.......700.......710.......720.......730.......740.......750

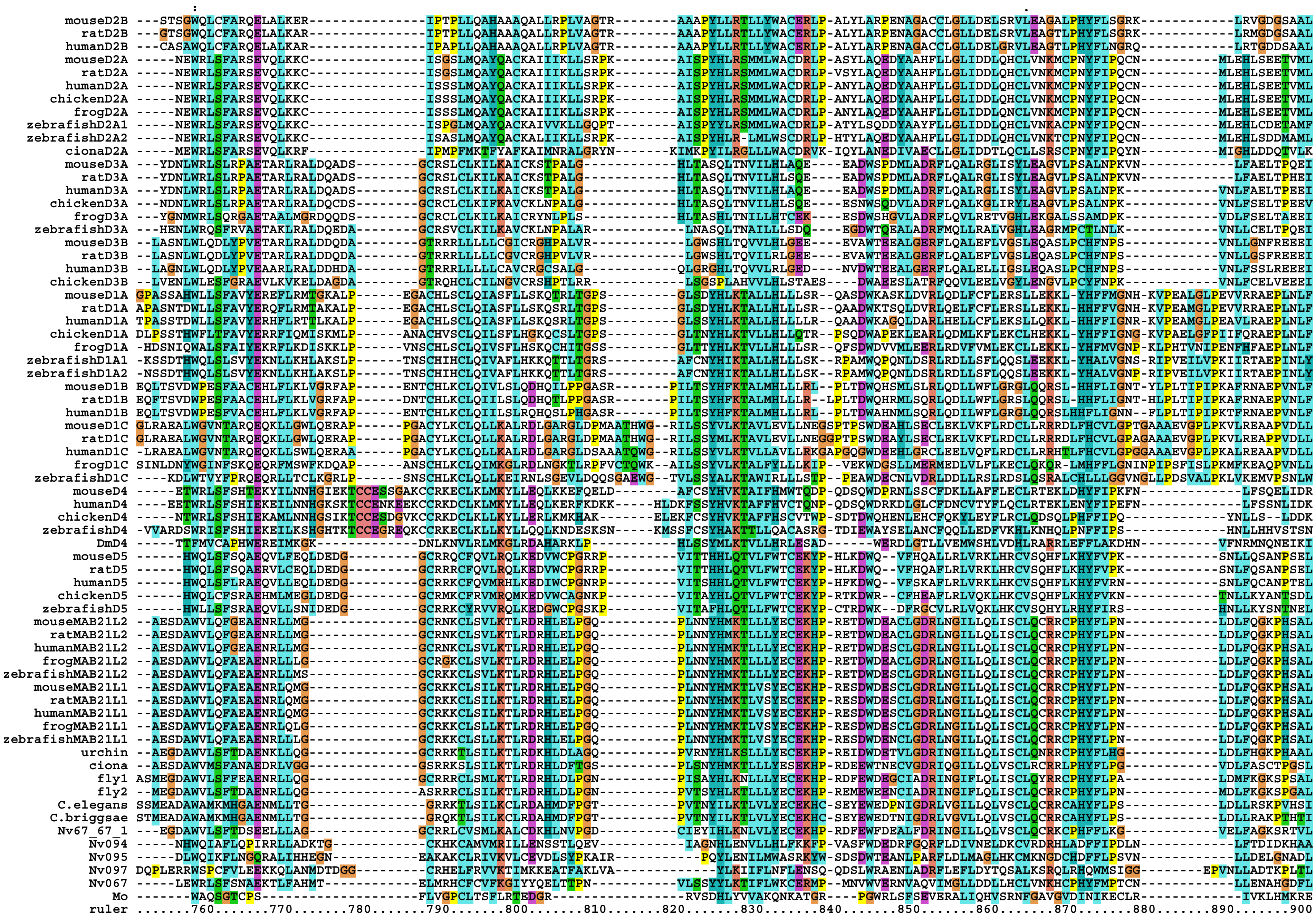
mouseD2B
ratD2B
humanD2B
mouseD2A
ratD2A
humanD2A
chickenD2A
frogD2A
zebrafishD2A1
zebrafishD2A2
cionaD2A
mouseD3A
ratD3A
humanD3A
chickenD3A
frogD3A
zebrafishD3A
mouseD3B
ratD3B
humanD3B
chickenD3B
mouseD1A
ratD1A
humanD1A
chickenD1A
frogD1A
zebrafishD1A1
zebrafishD1A2
mouseD1B
ratD1B
humanD1B
mouseD1C
ratD1C
humanD1C
frogD1C
zebrafishD1C
mouseD4
humanD4
chickenD4
zebrafishD4
DmD4
mouseD5
ratD5
humanD5
chickenD5
zebrafishD5
mouseMAB21L2
ratMAB21L2
humanMAB21L2
frogMAB21L2
zebrafishMAB21L2
mouseMAB21L1
ratMAB21L1
humanMAB21L1
frogMAB21L1
zebrafishMAB21L1
urchin
ciona
fly1
fly2
C.elegans
C.briggsae
Nv67_67_1
Nv094
Nv095
Nv097
Nv067
Mo
ruler

```
         mouseD2B RGALAQL---RGDPAQALREAVEEAKVARKGGGLAGVGGGTH---------------------------------------------------------------
           ratD2B RGALAQL---RGDPAQALREAVEEAKVARKGGGLAGVGGGTH---------------------------------------------------------------
         humanD2B LGELARL---RGDPARALRAAVEEAKVARKGGGLAGVGGGAH---------------------------------------------------------------
         mouseD2A HARKLSS--VRSDPAEHLRTAIEHVKAANRLTLKLQRRGSTTSIPSP-------QSDGGDPNQPDDRLAKKLQQLVTENPGKSISVFINPDDVTRPHFRIDDKFF
           ratD2A HARKLSS--VRSDPAEHLRTAIEHVKAANRLTLKLQRRGSTTSIPSP-------QSDGGDPNQPDDRLAKKLQQLVTENPGKSISVFINPDDVTRPHFRIDDKFF
         humanD2A HARKLSS--VRSDPAEHLRTAIEHVKAANRLTLELQRRGSTTSIPSP-------QSDGGDPNQPDDRLAKKLQQLVTENPGKSISVFINPDDVTRPHFRIDDKFF
       chickenD2A HARKLSS--VRSDPAEHLRTAIEHVKAANRLTLELQRRGSTTSIPSP-------QSDGGDTNQPDDRLAKKLQQLVTENPGKSISVFINPDDVTRPHFRIDDKFF
          frogD2A HARKLSS--VRSDPAEHLRTAIEHVKAANRLTLDLQRRGSTTSIPSP-------QSDGGDNNQPDDRLAKKLQQLVTENPGKSISVFINPDDVTRPHFRIDDKFF
    zebrafishD2A1 HARKLAL--VRSDPAEHLRSIIEQAKASNRLTQEMQRCGSSSGLPSP-----LADSSLADHQQPDDRLAKKLQQLVTENPGKSISVFINPDDVTRPHFRIDDKFF
    zebrafishD2A2 HARKLSS--VRSDPAEHLRSAIEHAKAASRLTVELQWRSGSSNLPSP-------LSETGADQQPDDRLAKKLQQLVTENPGKSISVFINPDDVTRPHFRIDDKFF
         cionaD2A LCQVVLM--VRSDPAEHVRNAVDQVKAARELTEAYHNQQMERKSTKSSSENGMSQSNVEMNSRQELDLAEKMRRLVDSNPGKSISVFLNPDDNSQPHFRIDGKFF
         mouseD3A DELGYTLYCSLSEP-EVLLQT------------------------------------------------------------------------------------
           ratD3A DELGYTLYCSLSEP-EVLLQT------------------------------------------------------------------------------------
         humanD3A DELGYTLYCSLSEP-EVLLQT------------------------------------------------------------------------------------
       chickenD3A DELGYTLYSSLSEP-EVLLQTHKPAPGRQKLPSYGQPPPRAAAALAVAVSAKAKDLGALCSCLENNQWRLSAHVNTTPPANQRAERYGFVGGQEQDPHPPKSP--
          frogD3A DEMGYFLYCSLSEP-EVLLRTED----------------------------------------------------------------------------------
     zebrafishD3A DELGYTLYCALSDP-ESLLRTV-----------------------------------------------------------------------------------
         mouseD3B DDIGYVLYSGLQVP-ESLF--------------------------------------------------------------------------------------
           ratD3B DDIGYVLYSGLQVP-ESLF--------------------------------------------------------------------------------------
         humanD3B DDIGYALYSGLQEP-EGLL--------------------------------------------------------------------------------------
       chickenD3B EEMGFLLYRAVSEP-GLLLTEK-----------------------------------------------------------------------------------
         mouseD1A RPFVLQRTLYRNTV-DSFYEMLKNAPALISEY----SLHVPSVRASPPPKAVVS---------------------------------------------------
           ratD1A RSFVLQRTLYRHTV-DSFYEMLKNAPTLISEY----SLHVPSDRASPPPKAVAL---------------------------------------------------
         humanD1A RPFVLQRSLYRKTL-DSFYEMLKNAPALISEY----SLHVPSDQPTPKS--------------------------------------------------------
       chickenD1A RPFVLHRDVYRKTI-DTFHEMLRNTSALISEY----MVHIPHVQTNGIHKESV----------------------------------------------------
          frogD1A RSFVLDRDLYQKTL-VMFYEMLKNATVLINEY----SLRLPASSLNNRYDLRELSRLFRSRYDLRELSQRNGYEKVATIRTSSNGYKKVVTFTKKSQQFT-----
    zebrafishD1A1 RPLVLQRHVYAKME-EHFEEMVRNTSVLVQEY----TPHFSNGHVRHEFSSAEQI--------------------------------------------------
    zebrafishD1A2 RPLVLQRHVYAKME-EHFEEMVRNTSVLVQEY----TPHFSNGHVRHEFSSAEQI--------------------------------------------------
         mouseD1B QHLVLNPVAHSQAV-EEFHNLLAQVKTLPCSP----VAGGL----------------------------------------------------------------
           ratD1B QHLVLNPVAHSQAV-EEFHNLLAQVKTLPCSP----LAGGL----------------------------------------------------------------
         humanD1B QHLVLNPKAHSQAV-EEFQNLLTQVKTLPHAP----LAAAP----------------------------------------------------------------
         mouseD1C APFDPHSRELAAAR-LLSTWRRLPQLLRVYG------RYLARCPAPRSQRIQGSPEDEP----------------------------------------------
           ratD1C APFDQHARELAAAR-LLSTWRRLPQLLQVYGP-----RYLARCPAPRSQRIQGFPEDEP----------------------------------------------
         humanD1C AAFDGHARELAAAR-LLSTWQRLPQLLRAYGG----PRYLARCPPPRSQRTQGFLEGEP----------------------------------------------
          frogD1C EGYGPDILDHTSFQ-LLNLWIQMPHILRINPS----PRNIPRELPRCKHAVFN----------------------------------------------------
     zebrafishD1C AEFSDVTLDMVSAR-LTYSWNHLPRLIRLGRP----QRTSLGRVVRCKYVDAE----------------------------------------------------
          mouseD4 KSKEFLSKKIEYER-NNGFPIFDKL--------------------------------------------------------------------------------
          humanD4 RSKEFLTKQIEYER-NNEFPVFDEF--------------------------------------------------------------------------------
        chickenD4 ASNDFLLGQINYQL-NNGFPVFHQV--------------------------------------------------------------------------------
      zebrafishD4 --CDFLAKEIEFQL-NNTFPIFS----------------------------------------------------------------------------------
             DmD4 CLENASTLLRKLCIAQTCGGSYHHVAQLFNIPN------------------------------------------------------------------------
          mouseD5 DAVAQKVAFFLKNP-QVELP-------------------------------------------------------------------------------------
            ratD5 DAVAQKVAFFLKNP-QLELP-------------------------------------------------------------------------------------
          humanD5 DTVAQKLATFLKNP-QIGPP-------------------------------------------------------------------------------------
        chickenD5 DMVASKLAVFLENP-VFCLD-------------------------------------------------------------------------------------
      zebrafishD5 DEVAKKINHFLENP-GTYIH-------------------------------------------------------------------------------------
      mouseMAB21L2 ESAAKQTWRLAREI-LTNPKSLDKL--------------------------------------------------------------------------------
        ratMAB21L2 ESAAKQTWRLAREI-LTNPKSLDKL--------------------------------------------------------------------------------
      humanMAB21L2 ESAAKQTWRLAREI-LTNPKSLDKL--------------------------------------------------------------------------------
       frogMAB21L2 ESAAKQTWRLAREI-LTNPKSLDKL--------------------------------------------------------------------------------
  zebrafishMAB21L2 ETAAKQTWRLAREI-LTNAKSLDKL--------------------------------------------------------------------------------
      mouseMAB21L1 ENAAKQTWRLAREI-LTNPKSLEKL--------------------------------------------------------------------------------
        ratMAB21L1 ENAAKQTWRLAREI-LTNPKSLEKL--------------------------------------------------------------------------------
      humanMAB21L1 ENAAKQTWRLAREI-LTNPKSLEKL--------------------------------------------------------------------------------
       frogMAB21L1 ENAAKQTWRLAREI-LTNPKSLEKL--------------------------------------------------------------------------------
  zebrafishMAB21L1 ENAAKQTWRLAREI-LTNPKSLEKL--------------------------------------------------------------------------------
           urchin DQAAKQAWRITREI-LTNPKSLEKL--------------------------------------------------------------------------------
            ciona DMAARQVWCLAREI-ITNSKSFDHL--------------------------------------------------------------------------------
             fly1 EQAAKQVWRLTREL-LTNANAFEKL--------------------------------------------------------------------------------
             fly2 ENAAKQVWRLTRIM-LTNVRCLEEL--------------------------------------------------------------------------------
        C.elegans EHSAQLAWHLVRKL-MIDPNALQSL--------------------------------------------------------------------------------
       C.briggsae EHSAKLTWHLVRKL-MIDPNALQTL--------------------------------------------------------------------------------
       Nv67_67_1 DQAAKQVWNLARDL-ATNPSSFE----------------------------------------------------------------------------------
           Nv094 QLARVELQKLTQNPVEFLKLSGNSQSTAF----------------------------------------------------------------------------
           Nv095 SVLSAKIMDVLVQPLKYLEI-------------------------------------------------------------------------------------
           Nv097 QNMESRVARILSSKSELRE--------------------------------------------------------------------------------------
           Nv067 LHLARTVLQIRRDPKRFFNRSRRDALLNPYARDHNKYKDKAASSAS-----------------------------------------------------------
              Mo AGGSPVKSYVLKTLWLHFVEAMEST--------------------------------------------------------------------------------
           ruler .......910.......920.......930.......940.......950.......960.......970.......980.......990......1000.....
```
