# Supplementary material for: Ancient Origin of the New Developmental Superfamily DANGER
Source: PLoS One. 2007 Feb 14;2(2):e204. doi: 10.1371/journal.pone.0000204 (PMC1784063; doi:10.1371/journal.pone.0000204)
Supplement: Figure S9 — The region 230–258 of the block formatted pairwise alignments shown in Figure 3 is magnified to demonstrate the positional conservation of insertions (magenta fonts) among the DANGER proteins. The amino acids in yellow bold fonts correspond to exon boundaries at the nucleotide level. Hs, H. sapiens; Dm, D. melanogaster; Ce, C. elegans; Nv, N. vectensis. (0.13 MB PDF) [file pone.0000204.s009.pdf]

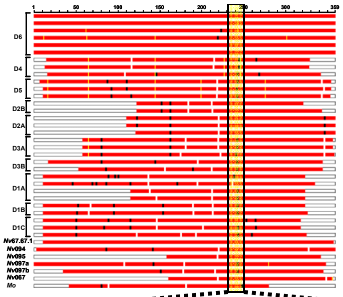

```
HsD2B vs HsL1    363 WQLCFARQELALKARIPAPLLQAHAAAQAL 393
                     W L FA  E  L+  +     +  +  + L
                 230 WVLQFAEAENRLQ--MGGCRKKCLSILKTL 258

HsD2A vs HsL1    285 WRLSFARSEVQLKKCISSSLMQAYQACKAI 313
                     W L FA +E +L+  +     +     K +
                 230 WVLQFAEAENRLQ--MGGCRKKCLSILKTL 258

HsD3A vs HsL1    337 WRLSLRPAETARLRALDQADSGCRSLCLKILKAI 371
                     W L    AE            GCR  CL ILK +
                 230 WVLQFAEAENRLQ------MGGCRKKCLSILKTL 258

HsD3B vs HsL1    329 WLQDLYPVEAARLRALDDHDAGTRRRLLLLLCAV 362
                     W+      E            G R++ L +L  +
                 230 WVLQFAEAENRLQ------MGGCRKKCLSILKTL 258

HsD1A vs HsL1    380 WLLSFAVYERHFLRTTLKALPEGACHLSCLQIASFL 416
                     W+L FA  E          L  G C   CL I   L
                 230 WVLQFAEAE--------NRLQMGGCRKKCLSILKTL 258

HsD1B vs HsL1    400 WPESFVACEHLFLKLVGRFAPENTCHLKCLQIILSL 436
                     W   F   E+              C  KCL I+ +L
                 230 WVLQFAEAENRLQ--------MGGCRKKCLSILKTL 258

HsD1C vs HsL1    344 WGVNTARQEQKLLSWLQERAAPGACYLKCLQLLKAL 379
                     W +  A  E +L          G C  KCL +LK L
                 230 WVLQFAEAENRLQM--------GGCRKKCLSILKTL 258

HsD4 vs HsL1     374 WRLSFSHIEKEILNNHGKSKTCCENKEEKCCRKDCLKLMKYL 416
                     W L F+  E  +                  CRK CL ++K L
                 230 WVLQFAEAENRLQMG--------------GCRKKCLSILKTL 258

HsD5 vs HsL1     234 WQLSFLRAEQVLLEQLDEDGGCRRKCFQVMRHL 266
                     W L F  AE  L       GGCR+KC  +++ L
                 230 WVLQFAEAENRLQ-----MGGCRKKCLSILKTL 258

DmD6a vs HsL1    230 WVLSFTDAENRLLQGASRRRCLSILKTL 258
                     WVL F +AENRL  G  R++CLSILKTL
                 230 WVLQFAEAENRLQMGGCRKKCLSILKTL 258

DmD6b vs HsL1    230 WVLSFFEAENRLLQGGCRRRCLSMLKTL 258
                     WVL F EAENRL  GGCR++CLS+LKTL
                 230 WVLQFAEAENRLQMGGCRKKCLSILKTL 258

CeD6 vs HsL1     236 WAMKMHGAENMLLTGG-RRKTLSILKCL 262
                     W ++   AEN L  GG R+K LSILK L
                 230 WVLQFAEAENRLQMGGCRKKCLSILKTL 258
```

Nikolaidis et al. fig. S9

```
Nv233 vs HsL1     280 WRMSFSLAENRLAQSLTPVQRHTLVLLK-I 310
                      W + F+ AENRL       ++  L +LK +
                  230 WVLQFAEAENRLQMG--GCRKKCLSILKTL 258

Nv051 vs HsL1     282 WRLSFSVAENILGQNISPLQRYLIVLMKM 310
                      W L F+ AEN L       ++ L +L  +
                  230 WVLQFAEAENRLQMG-GCRKKCLSILKTL 258

Nv094 vs HsL1     228 WQIAFLQPIRRLLADKTGCKHKCAMVMRIL 257
                      W + F +   RL     GC+ KC  +++ L
                  230 WVLQFAEAENRLQM--GGCRKKCLSILKTL 258

Nv095 vs HsL1     437 WQIKFLNGQRALIHHEGNEAKAKCLRIVKVL 468
                      W ++F   +  L   +    + KCL I+K L
                  230 WVLQFAEAENRL---QMGGCRKKCLSILKTL 258

Nv137 vs HsL1     208 WRYSFSTAEKKLFQKGFSGDAGTCMKQVLRLLKSL 242
                      W   F+ AE +L   G       C K+ L +LK+L
                  230 WVLQFAEAENRLQMGG-------CRKKCLSILKTL 258

Nv097A vs HsL1    204 FRQSFSVKEKAILRHMDSGDGGCRHILLRIVKTM 210
                      +   F+  E  +        GGCR   L I+KT+
                  230 WVLQFAEAENRLQ------MGGCRKKCLSILKTL 258

Nv097B vs HsL1    557 WSPCFVLEEKKQLANMDTDGGCRHELFRVVKTI 588
                      W   F   E +        GGCR +   ++KT+
                  230 WVLQFAEAENRLQ-----MGGCRKKCLSILKTL 258

Nv067 vs HsL1     312 WRLSFSNAEKTLFAHMTELMRHCFCVFKGI 341
                      W L F+ AE  L        + C  + K +
                  230 WVLQFAEAENRLQMGGCR--KKCLSILKTL 258

Nv037 vs HsL1     311 WRISFSKAEAALFNSMSPPMMHCYRIFKTI 340
                      W + F++AE  L          C  I KT+
                  230 WVLQFAEAENRLQMGGC--RKKCLSILKTL 258

Nv039 vs HsL1     371 WRNSFSLDEKRKLYNIDKKDKGCRHEVIRIAKSI 404
                      W   F+  E R          GCR + + I K++
                  230 WVLQFAEAENRLQ------MGGCRKKCLSILKTL 258

Nv008 vs HsL1     328 -RISYSLHEQQIMSEFPLDTG-QNQCLRLLKYF 358
                        + ++  E ++        G + +CL +LK
                  258 WVLQFAEAENRL-----QMGGCRKKCLSILKTL 258

Nv010 vs HsL1     373 WRISFSKAEAALFYSMSPPMMHCYRIFKAI 403
                      W + F++AE  L   M      C  I K +
                  230 WVLQFAEAENRLQ--MGGCRKKCLSILKTL 258

Nv155 vs HsL1     426 FRQSFSLKEKEIFNRLDGQGNGCKKEVLKIMKTI 459
                      +   F+  E  +      Q  GC+K+ L I+KT+
                  230 WVLQFAEAENRL------QMGGCRKKCLSILKTL 258

Nv016 vs HsL1     293 WRLSFYVGEKNKLRAIGTEGCRIPLLRILTEI 324
                      W L F   E      +   GCR   L IL  +
                  230 WVLQFAEAEN----RLQMGGCRKKCLSILKTL 258
```

Nikolaidis et al. fig. S9
