# Supplementary material for: Ancient Origin of the New Developmental Superfamily DANGER
Source: PLoS One. 2007 Feb 14;2(2):e204. doi: 10.1371/journal.pone.0000204 (PMC1784063; doi:10.1371/journal.pone.0000204)
Supplement: Figure S11 — zPicture visualization of representative alignments among DANGER genomic sequences. zPicture uses the local alignments tool BLASTZ to generate sequence alignments between a reference sequence (the first one) and one or more sequences. BLASTZ identifies matches independent of their linear organization in the input sequences and zPicture maps these alignments onto the reference (first) sequence. Local alignments are visualized as standard percent identity plots. (A) Visualizations of pairwise alignments among H. sapiens (h) DANGER paralogs. Note: When all sequences are masked for transposable elements (TEs) no similarity is found, except between HsD1A–HsD1B coding sequences. (B) Visualizations of pairwise alignments among M. musculus (m) DANGER paralogs. Note: When all sequences are masked for TEs no similarity is found, except between MmD1A–MmD1B coding sequences. (C) Visualizations of pairwise alignments among D. rerio (z) DANGER paralogs. Note: When all sequences are masked for TEs no similarity is found, except between DrD1A1–DrD1A2 coding sequences. (D) Upper: visualizations of pairwise alignments among D1A orthologous sequences from H. sapiens, M. musculus, and D. rerio. Lower: visualizations of pairwise alignments among D1A orthologous sequences from H. sapiens, M. musculus, and D. rerio after masking of TEs. (E) Upper: visualizations of pairwise alignments among MAB21L1 (L1) orthologous sequences from H. sapiens, M. musculus, and D. rerio. Lower: visualizations of pairwise alignments among L1 orthologous sequences from H. sapiens, M. musculus, and D. rerio after masking of TEs. (F) Upper: visualization of pairwise alignments between L1 and L2 paralogous sequences. Lower: Visualizations of pairwise alignments between L1 and L2 paralogous sequences, after masking of TEs. (G) Visualizations of pairwise alignments between HsD2B and HsD2A genomic regions. (H) Visualizations of pairwise alignments between HsD3A and HsBCR genomic regions. (I) Visualizations of pairwi [file pone.0000204.s011.pdf]

A

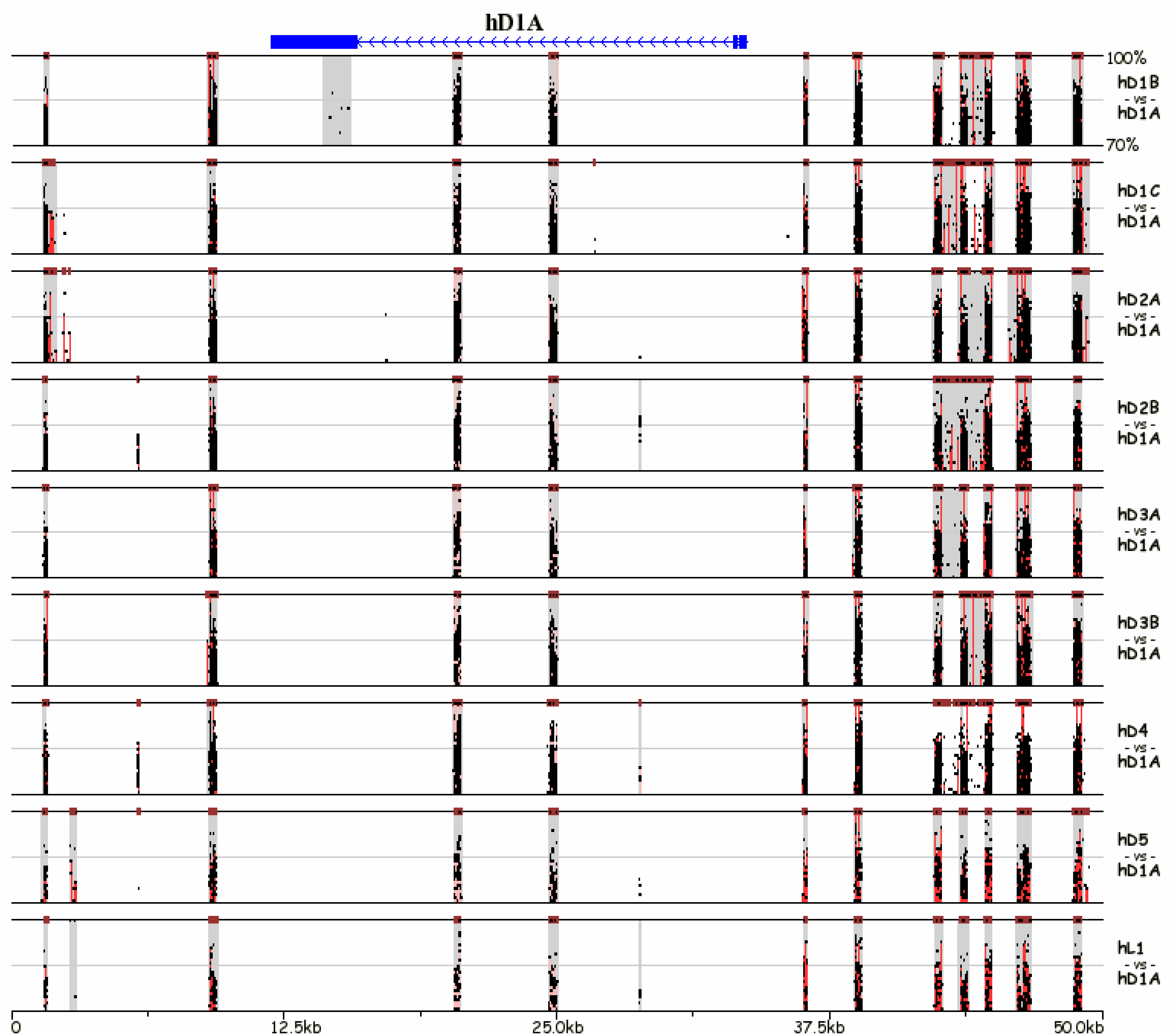

Nikolaidis et al. fig. S11

B

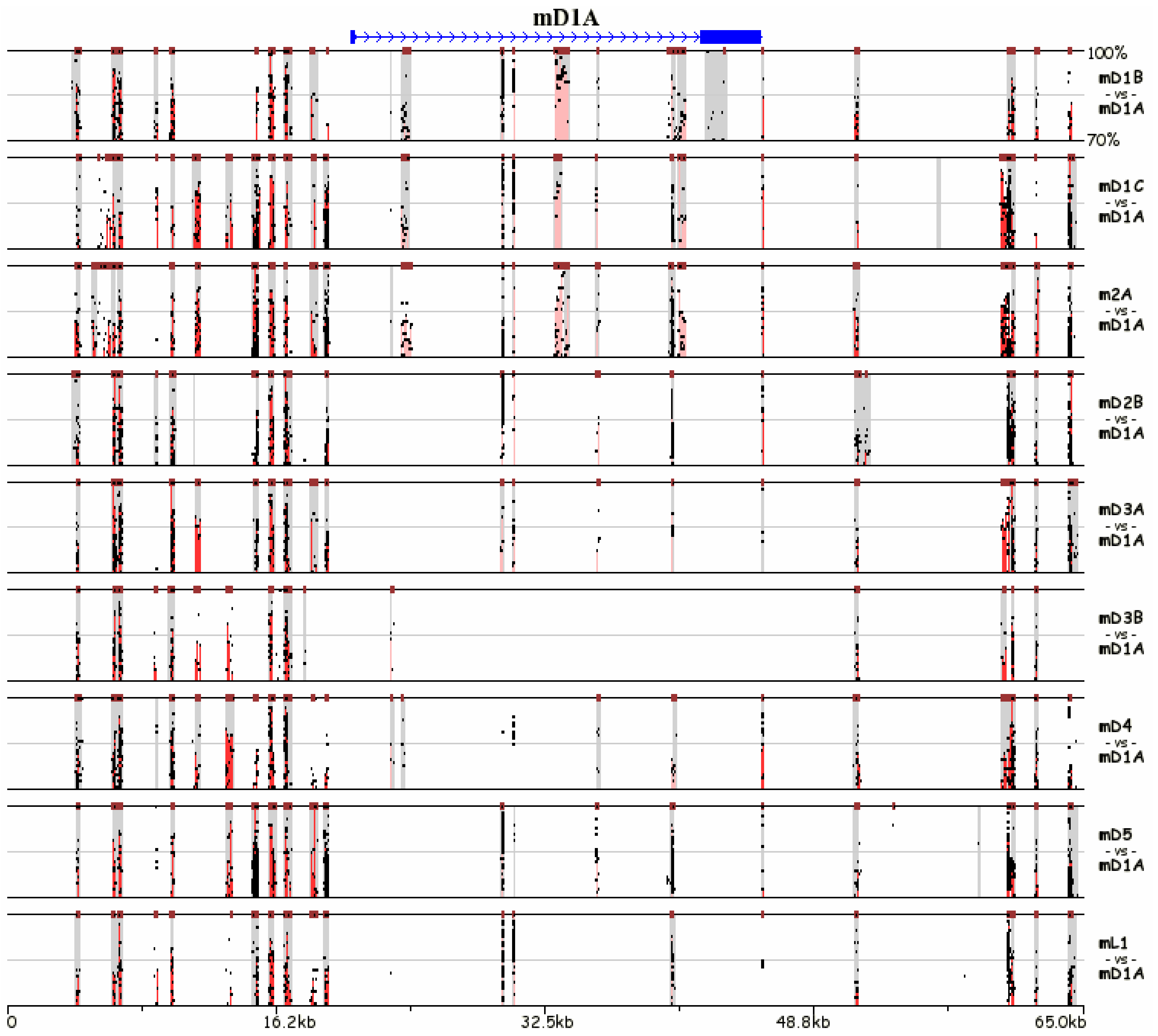

Nikolaidis et al. fig. S11

C

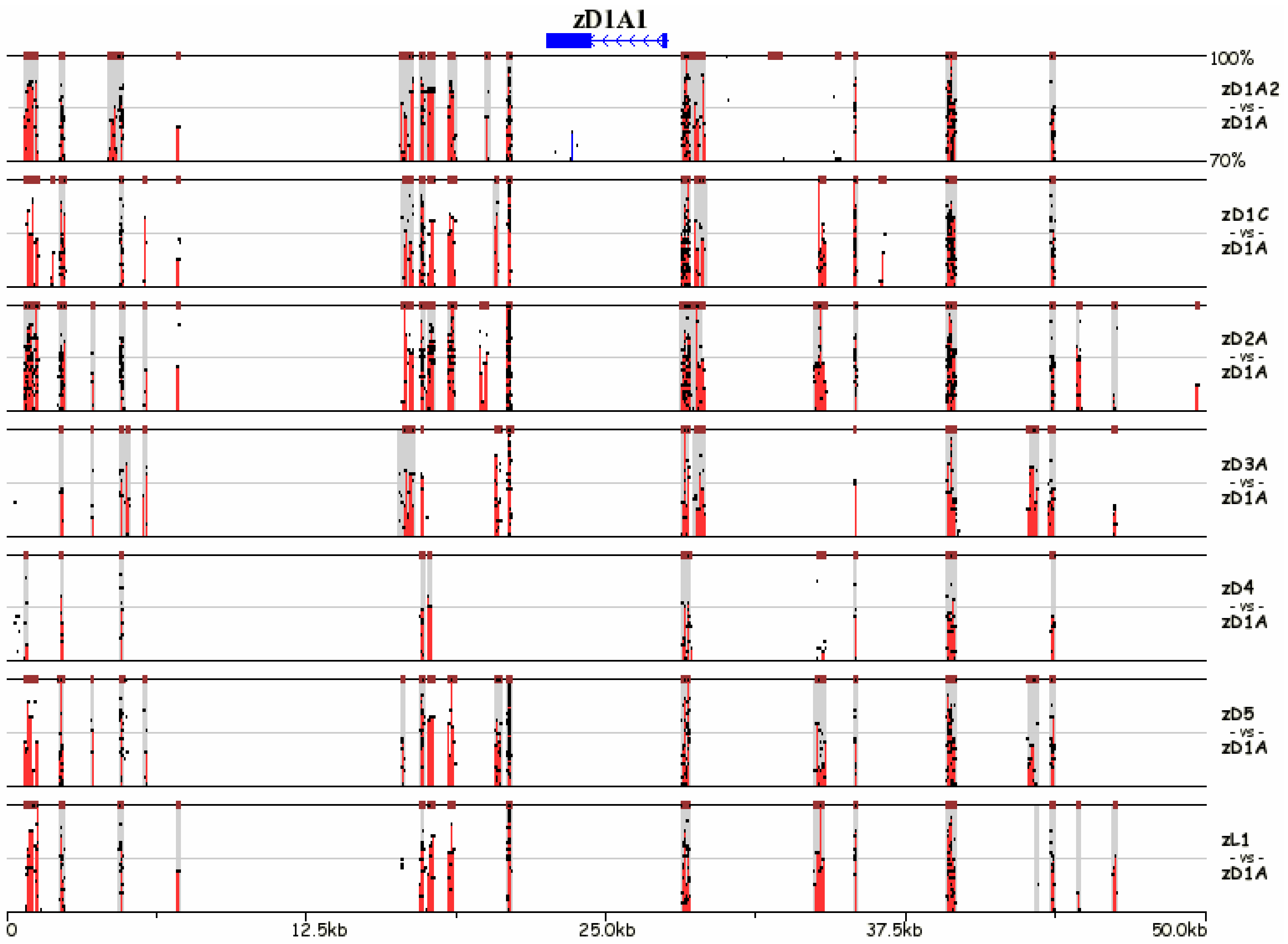

Nikolaidis et al. fig. S11

D

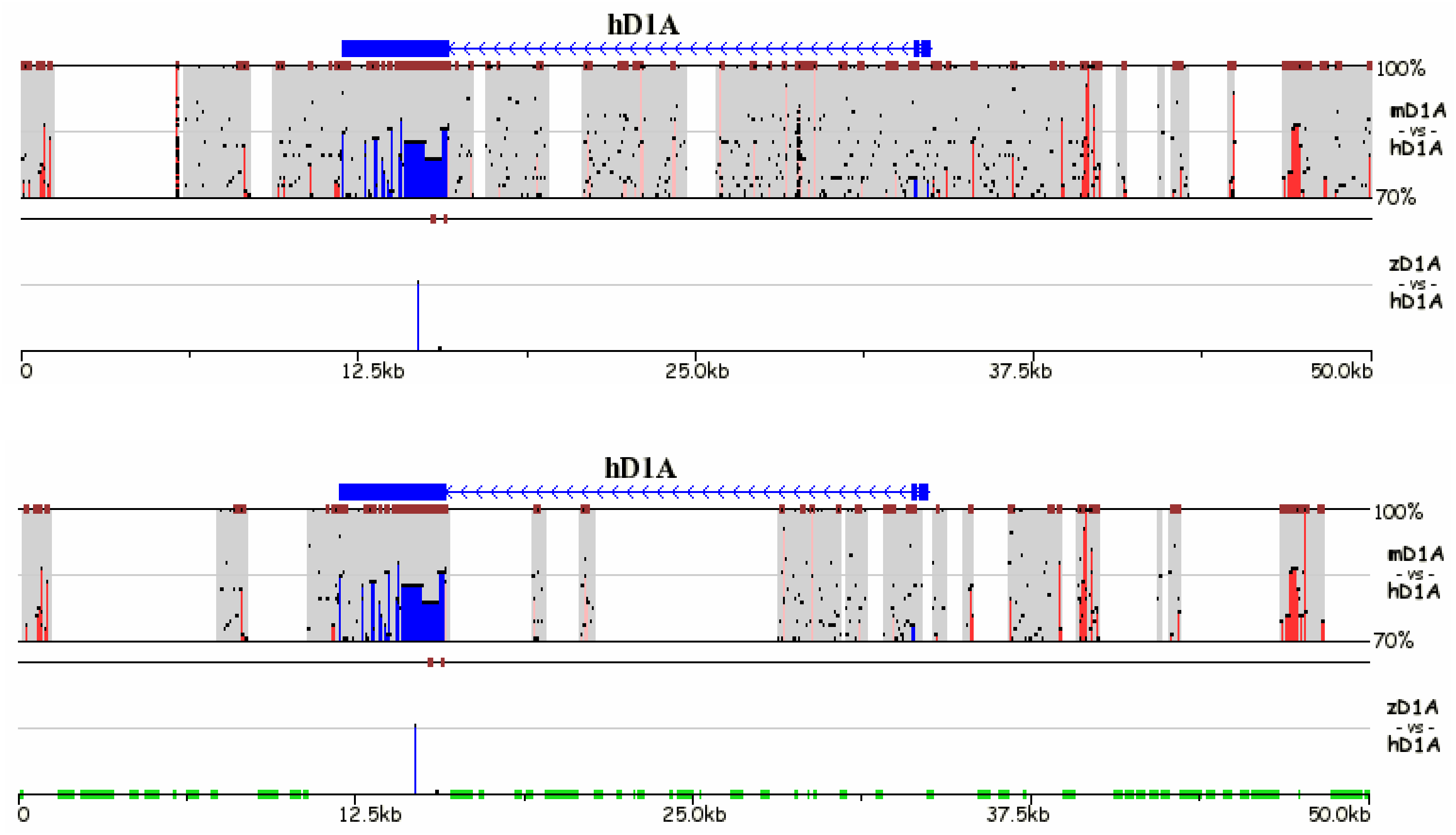

Nikolaidis et al. fig. S11

E

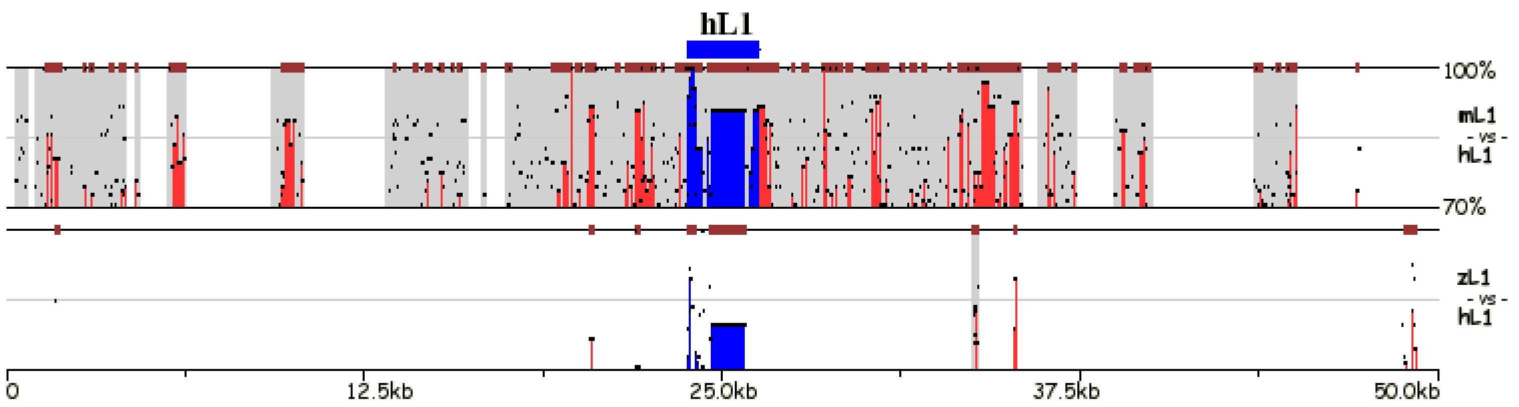

When all sequences are masked

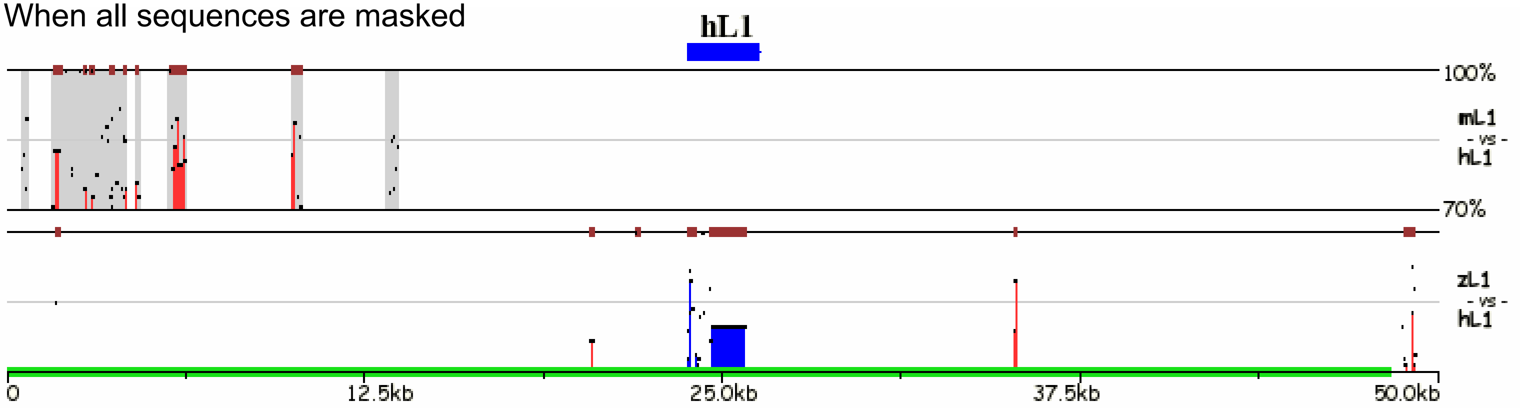

Nikolaidis et al. fig. S11

F

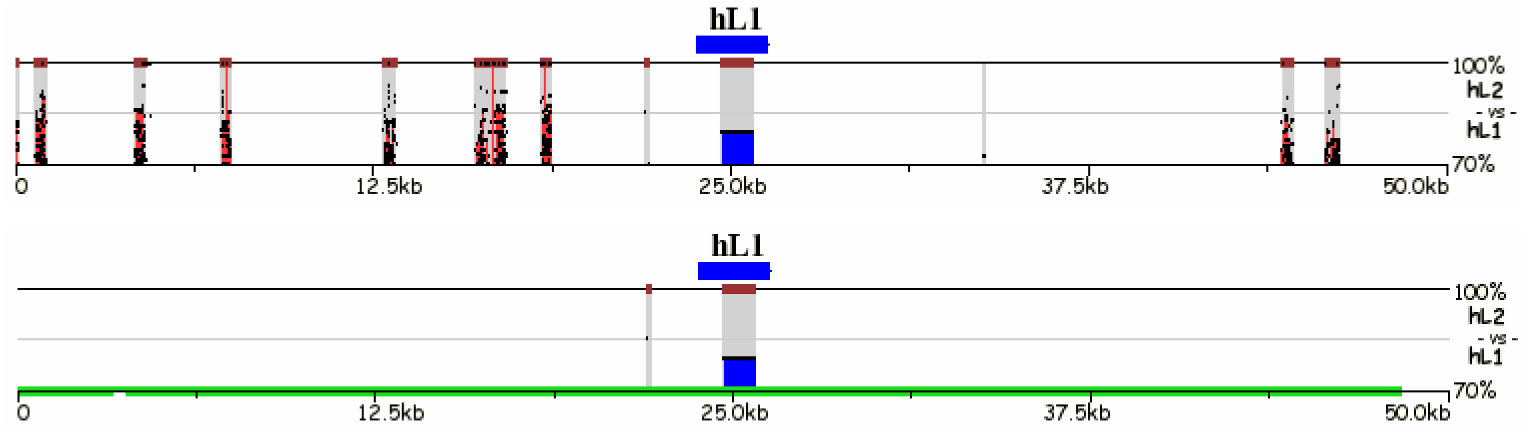

G

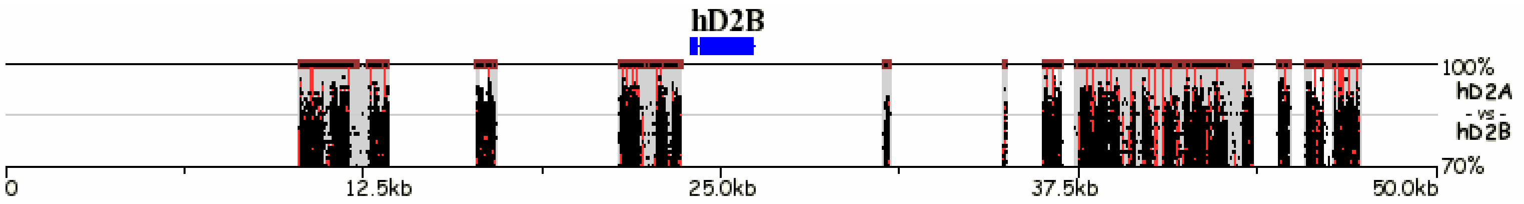

When both sequences are masked no similarity is found

H

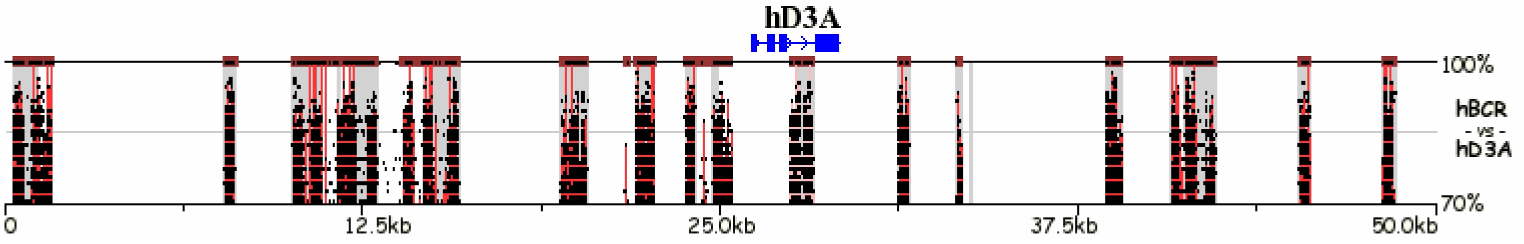

When both sequences are masked no similarity is found

I

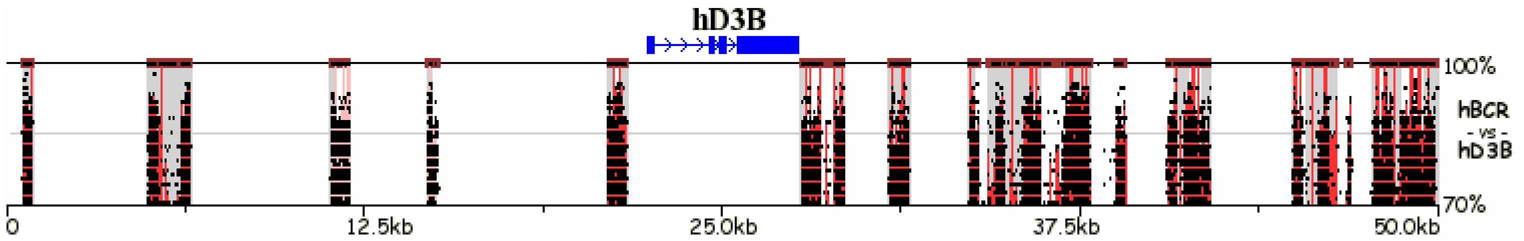

When both sequences are masked no similarity is found

Nikolaidis et al. fig. S11

J

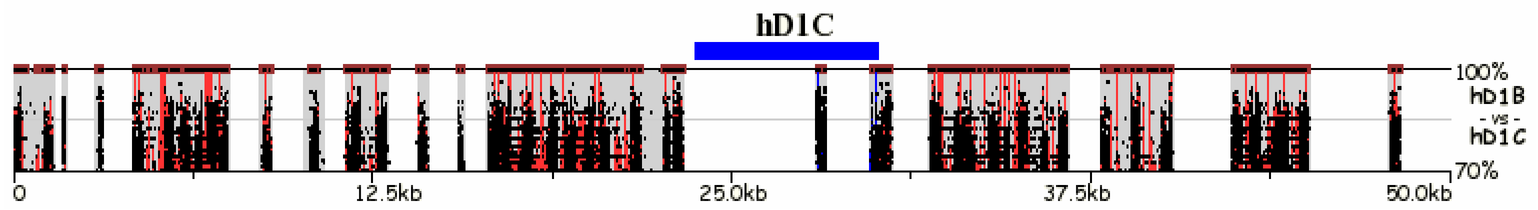

When TEs are masked, no similarity is found

K

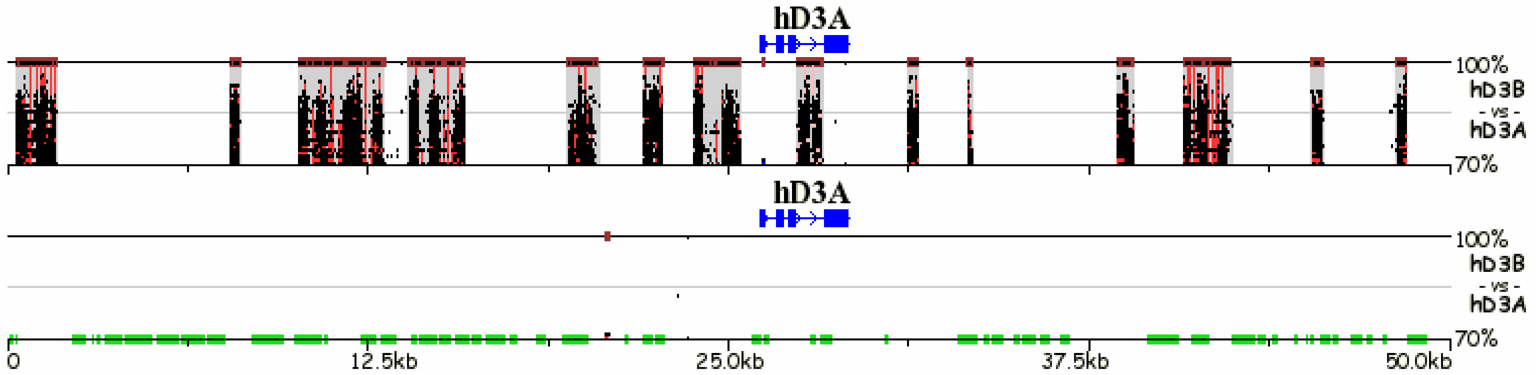

L

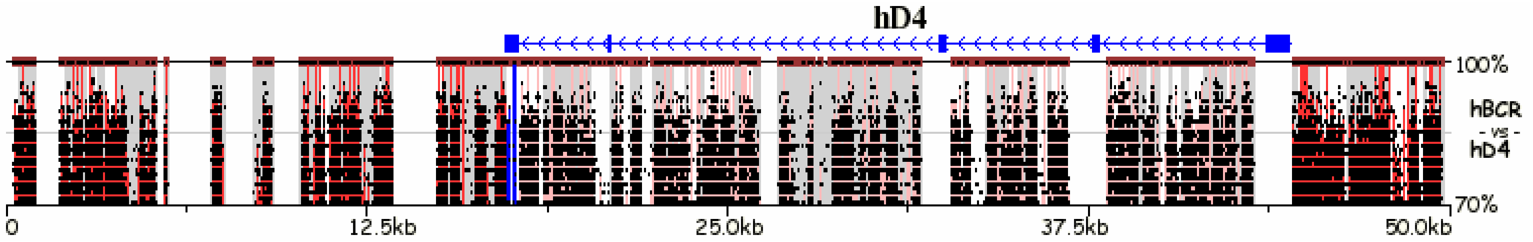

When TEs are masked, no similarity is found

Nikolaidis et al. fig. S11
