# Supplementary material for: Ancient Origin of the New Developmental Superfamily DANGER
Source: PLoS One. 2007 Feb 14;2(2):e204. doi: 10.1371/journal.pone.0000204 (PMC1784063; doi:10.1371/journal.pone.0000204)
Supplement: Table S1 — List of sequences used in this study (0.01 MB PDF) [file pone.0000204.s016.pdf]

Table S1. List of sequences used in this study

Species	# per species	Accesion number	Database	Group	Family	Chromosome
Caenorhabditis briggsae	1	AAG36973	NCBI	MAB-21	D6	Unknown
Caenorhabditis elegans	1	NP_497940	NCBI	MAB-21	D6	III
Ciona intestinalis	1	ENSCING00000001067	Ensembl (assembly JGI2)	D2A	D2	Unknown
Ciona intestinalis	2	ENSCING00000008580	Ensembl (assembly JGI2)	MAB-21	D6	2
Danio rerio	1	XP_701813	NCBI	D1A	D1	13
Danio rerio	2	ENSDARP00000052997	Ensembl (assembly Zv 6)	D1A	D1	1
Danio rerio	3	XP_692664	NCBI	D2A	D2	Unknown
Danio rerio	4	XP_684668	NCBI	D2A	D2	1
Danio rerio	5	XP_690094	NCBI	D3A	D3	3
Danio rerio	6	XP_685111	NCBI	D4	D4	13
Danio rerio	7	XP_686443	NCBI	D5	D5	9
Danio rerio	8	ENSDARG00000015266	Ensembl (assembly Zv 6)	MAB21L2	D6	1
Danio rerio	9	ENSDARG00000055089	Ensembl (assembly Zv 6)	MAB21L1	D6	15
*Danio rerio**	10	NC_007127:c24759168-24675421*	NCBI	D1C	D1	16
Drosophila melanogaster	1	CG15865	NCBI	?	?	X
Drosophila melanogaster	2	CG7194	NCBI	D4	D4	3L
Drosophila melanogaster	3	CG4746	NCBI	MAB-21	D6	X
Drosophila melanogaster	4	CG4766	NCBI	MAB-21	D6	X
Gallus gallus	1	XP_426536	NCBI	D1A	D1	6
Gallus gallus	2	XP_422716	NCBI	D2A	D2	9

Gallus gallus	3	XP_416248	NCBI	D3A	D3	1
Gallus gallus	4	XP_414820	NCBI	D3B	D3	14
Gallus gallus	5	XP_419881	NCBI	D4	D4	3
*Gallus gallus**	6	NC_006088:c2189076-2251130*	NCBI	D5	D5	1
Gallus gallus	7	NP_989864	NCBI	MAB21L1	D6	1
*Gallus gallus***	8	NP_989521**	NCBI	MAB21L2	D6	4
Homo sapiens	1	NP_203755	NCBI	D1A	D1	10
Homo sapiens	2	NP_848590	NCBI	D1B	D1	2
Homo sapiens	3	XP_091331	NCBI	D1C	D1	16
Homo sapiens	4	NP_848591	NCBI	D2A	D2	3
Homo sapiens	5	NP_848613	NCBI	D2B	D2	17
Homo sapiens	6	NP_061881	NCBI	D3A	D3	22
Homo sapiens	7	NP_683684	NCBI	D3B	D3	17
Homo sapiens	8	CAI14878	NCBI	D4	D4	6
Homo sapiens	9	NP_689580	NCBI	D5	D5	1
Homo sapiens	10	NP_006430	NCBI	MAB21L2	D6	4
Homo sapiens	11	CAG33701	NCBI	MAB21L1	D6	13
Monosiga ovata	1	MNL00001870	TBestDB	Mo_DANGER	?	Unknown
Mus musculus	1	AAH63749	NCBI	D1A	D1	19
Mus musculus	2	XP_485065	NCBI	D1B	D1	2
Mus musculus	3	XP_485992	NCBI	D1C	D1	7
Mus musculus	4	NP_808386	NCBI	D2A	D2	16
Mus musculus	5	BAE25328	NCBI	D2B	D2	11

Mus musculus	6	NP_848834	NCBI	D3A	D3	15
Mus musculus	7	BAE33897	NCBI	D3B	D3	11
Mus musculus	8	BAC35733	NCBI	D4	D4	9
Mus musculus	9	NP_758499	NCBI	D5	D5	3
Mus musculus	10	NP_035969	NCBI	MAB21L2	D6	3
Mus musculus	11	NP_034880	NCBI	MAB21L1	D6	3
Nematostella vectensis	1	gw.67.67.1	DOE JGI (version 1.0)	Nv 67_67_1	D6	Unknown
Nematostella vectensis	2	fgenesh1_pg.scaffold_145000008	DOE JGI (version 1.0)	Nv08	D4 ?	Unknown
Nematostella vectensis	3	fgenesh1_pg.scaffold_375000010	DOE JGI (version 1.0)	Nv10	D2 ?	Unknown
Nematostella vectensis	4	fgenesh1_pg.scaffold_55000137	DOE JGI (version 1.0)	Nv137	D4 ?	Unknown
Nematostella vectensis	5	estExt_fgenesh1_pg.C_10155	DOE JGI (version 1.0)	Nv155	?	Unknown
Nematostella vectensis	6	estExt_fgenesh1_pg.C_1950016	DOE JGI (version 1.0)	Nv16	D3 ?	Unknown
Nematostella vectensis	7	fgenesh1_pg.scaffold_2000233	DOE JGI (version 1.0)	Nv233	?	Unknown
Nematostella vectensis	8	fgenesh1_pg.scaffold_81000037	DOE JGI (version 1.0)	Nv37	D2 ?	Unknown
Nematostella vectensis	9	fgenesh1_pg.scaffold_134000039	DOE JGI (version 1.0)	Nv39	D1 ?	Unknown
Nematostella vectensis	10	fgenesh1_pg.scaffold_18000051	DOE JGI (version 1.0)	Nv51	?	Unknown
Nematostella vectensis	11	fgenesh1_pg.scaffold_65000067	DOE JGI (version 1.0)	Nv67	D2 ?	Unknown
Nematostella vectensis	12	fgenesh1_pg.scaffold_55000094	DOE JGI (version 1.0)	Nv94	D5 ?	Unknown
Nematostella vectensis	13	fgenesh1_pg.scaffold_55000095	DOE JGI (version 1.0)	Nv95	?	Unknown
Nematostella vectensis	14	fgenesh1_pg.scaffold_61000097	DOE JGI (version 1.0)	Nv97a	D3 ?	Unknown
Nematostella vectensis	15	fgenesh1_pg.scaffold_61000097	DOE JGI (version 1.0)	Nv97b	D3 ?	Unknown
Rattus norvegicus	1	XP_575230	NCBI	D1B	D1	3
Rattus norvegicus	2	XP_574546	NCBI	D1C	D1	1

Rattus norvegicus	3	XP_573305	NCBI	D2A	D2	11
Rattus norvegicus	4	ENSRNOG00000015057	Ensembl (assembly RGSC 3.4)	D2B	D2	10
Rattus norvegicus	5	NP_001007710	NCBI	D3A	D3	7
Rattus norvegicus	6	XP_573103	NCBI	D3B	D3	10
Rattus norvegicus	7	XP_227522	NCBI	D5	D5	2
Rattus norvegicus	8	XP_227492	NCBI	MAB21L2	D6	2
Rattus norvegicus	9	ENSRNOG00000032941	Ensembl (assembly RGSC 3.4)	MAB21L1	D6	2
*Rattus norvegicus**	10	AC094902, clone CH230-6J13*	NCBI	D1A	D1	Unknown
Strongylocentrotus purpuratus	1	XP_794693	NCBI	?	?	Unknown
Strongylocentrotus purpuratus	2	XP_779896	NCBI	D6	D6	Unknown
Xenopus laevis	1	AAF67175	NCBI	MAB21L2	D6	Unknown
Xenopus laevis	2	AAH72806	NCBI	MAB21L1	D6	Unknown
Xenopus tropicalis	3	estExt_fgenesh1_pg.C_210027	DOE JGI (v 4.1)	D1C	D1	Unknown
Xenopus tropicalis	4	NP_001025698	NCBI	D3A	D3	Unknown
*Xenopus tropicalis**	5	scaffold_1143*	DOE JGI (v 4.1)	D1A	D1	Unknown
*Xenopus tropicalis**	6	scaffold_81*	DOE JGI (v 4.1)	D2A	D2	Unknown

Note: * denotes that the gene was predicted; ** denotes a pseudogene
