# Supplementary material for: Ancient Origin of the New Developmental Superfamily DANGER
Source: PLoS One. 2007 Feb 14;2(2):e204. doi: 10.1371/journal.pone.0000204 (PMC1784063; doi:10.1371/journal.pone.0000204)
Supplement: Table S2 — Repetitive elements found in DANGER mRNA sequences. Numbers correspond to the mRNA coordinates. (0.01 MB PDF) [file pone.0000204.s017.pdf]

Table S2. Repetitive elements found in DANGER mRNA sequences. Numbers correspond to the mRNA coordinates

Query	query begin	query end	repeat	class/family
**HsL1**	-	-	-	-
**HsL2**	-	-	-	-
**HsD1A**	-	-	-	-
**HsD1B**	-	-	-	-
**HsD1C**	4199	4497	AluSx	SINE/Alu
**HsD1C**	5478	5610	Charlie8	DNA/MER1_type
**HsD1C**	6098	6309	AluJo	SINE/Alu
**HsD2A**	-	-	-	-
**HsD2B**	-	-	-	-
**HsD3A**	3990	4282	AluSx	SINE/Alu
**HsD3B**	-	-	-	-
**HsD4**	1724	1887	AluSg/x	SINE/Alu
**HsD5**	1760	1818	MIRb	SINE/MIR
**HsD5**	2298	2439	L2	LINE/L2
**HsD5**	2559	2691	L2	LINE/L2
**HsD5**	3011	3229	L1P1	LINE/L1
**MmL1**	-	-	-	-
**MmL2**	-	-	-	-
**MmD1A**	-	-	-	-
**MmD1B**	-	-	-	-
**MmD1C**	2575	2649	ID4_	SINE/ID
**MmD1C**	5145	5248	MER102b	DNA/MER1_type
**MmD2A**	-	-	-	-
**MmD2B**	-	-	-	-
**MmD3A**	-	-	-	-
**MmD3B**	-	-	-	-
**MmD4**	1738	1865	B1_Mus2	SINE/Alu
**MmD4**	1890	2075	B4	SINE/B4
**MmD4**	2125	2152	ID_B1	SINE/B4
**MmD4**	2153	2504	ORR1A4	LTR/MaLR
**MmD4**	2505	2586	ID_B1	SINE/B4
**MmD4**	2587	2733	B3	SINE/B2
**MmD4**	2792	2904	PB1D7	SINE/Alu
**MmD5**	2761	2820	RMER15	LTR/ERVL
**MmD5**	3079	3197	B1F	SINE/Alu
